# Discovery of potential prognostic long non-coding RNA biomarkers for predicting the risk of tumor recurrence of breast cancer patients

**DOI:** 10.1038/srep31038

**Published:** 2016-08-09

**Authors:** Meng Zhou, Lei Zhong, Wanying Xu, Yifan Sun, Zhaoyue Zhang, Hengqiang Zhao, Lei Yang, Jie Sun

**Affiliations:** 1 College of Bioinformatics Science and Technology, Harbin Medical University, Harbin 150081, PR China; 2 Department of General Surgery, Second Affiliated Hospital of Harbin Medical University, Harbin Medical University, Harbin 150086, PR China

## Abstract

Deregulation of long non-coding RNAs (lncRNAs) expression has been proven to be involved in the development and progression of cancer. However, expression pattern and prognostic value of lncRNAs in breast cancer recurrence remain unclear. Here, we analyzed lncRNA expression profiles of breast cancer patients who did or did not develop recurrence by repurposing existing microarray datasets from the Gene Expression Omnibus database, and identified 12 differentially expressed lncRNAs that were closely associated with tumor recurrence of breast cancer patients. We constructed a lncRNA-focus molecular signature by the risk scoring method based on the expression levels of 12 relapse-related lncRNAs from the discovery cohort, which classified patients into high-risk and low-risk groups with significantly different recurrence-free survival (HR = 2.72, 95% confidence interval 2.07–3.57; p = 4.8e-13). The 12-lncRNA signature also represented similar prognostic value in two out of three independent validation cohorts. Furthermore, the prognostic power of the 12-lncRNA signature was independent of known clinical prognostic factors in at least two cohorts. Functional analysis suggested that the predicted relapse-related lncRNAs may be involved in known breast cancer-related biological processes and pathways. Our results highlighted the potential of lncRNAs as novel candidate biomarkers to identify breast cancer patients at high risk of tumor recurrence.

The increasing attention to various types of non-coding RNAs (ncRNAs) has highlighted their well-adapted and specialized biological roles during past years^1^,^2^. Long non-coding RNAs (lncRNAs), a newly discovered class of ncRNAs, were defined as RNA molecules longer than 200 nucleotides in length that do not belong to known categories of small RNAs and structural RNAs^3^. Transcriptome analysis showed that the majority of lncRNAs were expressed at lower levels and in a more cell type-, tissue- and developmental stage-specific manners compared to protein-coding genes^4^,^5^. Though most of lncRNAs have not been functionally characterized yet, there is growing evidence that lncRNAs are involved in a spectrum of biological processes, such as development, maintenance of pluripotency^6^, nuclear organization^7^, genomic imprinting^8^, RNA splicing and translational control^9^. Differences in expression patterns of lncRNAs have been observed between normal human tissues and cancers^10^, and many differentially expressed lncRNAs were identified in various cancers by cancer transcriptome profiling analysis. A growing number of dysregulated lncRNAs were better characterized as oncogenes or tumor suppressor genes contributing to cancer development, progression and metastasis^11^. For example, lncRNA *MALAT1* function as an oncogene whose high expression was associated with high metastatic potential and poor patient prognosis of lung cancer^12^. Zhou and colleagues observed the loss of lncRNA *MEG3* expression in many primary human tumors and tumor cell lines, providing substantial evidence that supported lncRNA *MEG3* as a tumor suppressor^13^. An increasing amount of clinical investigations and studies about lncRNAs has highlighted the potential and importance of lncRNAs as novel biomarkers and/or therapeutic targets for cancer diagnosis and therapy^14^,^15^,^16^,^17^,^18^,^19^,^20^,^21^,^22^,^23^,^24^.

Breast cancer is a frequent malignant gynecologic cancer accounting for 29% of all newly diagnosed cancers among women in 2014, and is one of the major causes of cancer death among women^25^. Although improvements in early diagnosis by mammographic screening and biomarker detection, tumor recurrence (local recurrence or distant recurrence) following conventional therapies is a major cause of morbidity and mortality in patients with breast cancer. It is a critical need to identify biomarkers that could predict breast cancer recurrence or recurrence-free survival. Several prognostic biomarkers for breast cancer recurrence have been established at the protein-coding gene and miRNA levels, such as 70-gene MammaPrit panel^26^, 21-gene Oncotype DX assay panel^27^, 36-gene signature^28^ and 5-miRNA signature^29^. As breast cancer is a highly clinically and molecularly heterogeneous disease, our understanding of the molecular mechanisms underlying breast cancer recurrence is far from clear. Recently, some studies have found that altered lncRNA expression is associated with tumor recurrence in hepatocellular carcinoma^30^, gliomas^31^, bladder cancer^32^ and colorectal cancer^33^, highlighting the potential function for lncRNAs as novel biomarkers to predict tumor recurrence. In breast cancer, several lncRNAs have been found to be involved in patient’s survival and metastasis^34^,^35^,^36^. However, the expression pattern and prognostic value of lncRNAs in breast cancer recurrence have not been investigated.

In this work, we performed a comprehensive analysis of lncRNA expression profiles across 473 breast cancer patients who did or did not develop recurrence by repurposing the publicly available microarray expression profiles from the Gene Expression Omnibus (GEO) database. A comparison between the groups of patients who did and did not develop recurrence identified a set of 12 lncRNAs overexpressed or downregulated in relapsed patients. Finally, we defined a 12-lncRNA signature by risk scoring method that is highly predictive of tumor recurrence and recurrence-free survival of breast cancer patients.

## Results

### Identification of lncRNAs associated with tumor recurrence of breast cancer patients

In this study, we included four breast cancer patient cohorts with recurrence information for biomarker discovery and validation (see Supplementary Table S1). The patient cohort from Clarke’s study, including 48 relapsed patients and 56 non-relapsed patients, was selected as a discovery cohort to identify lncRNA biomarkers associated with tumor recurrence of breast cancer patients (hereafter inferred as discovery cohort), and the patient cohorts from Bos’s study, Loi’s study and Dedeurwaerder’s study were used as additional independent test cohorts for validation purpose (hereafter referred as test cohort-1, test cohort-2 and test cohort-3).

**Figure 1:**
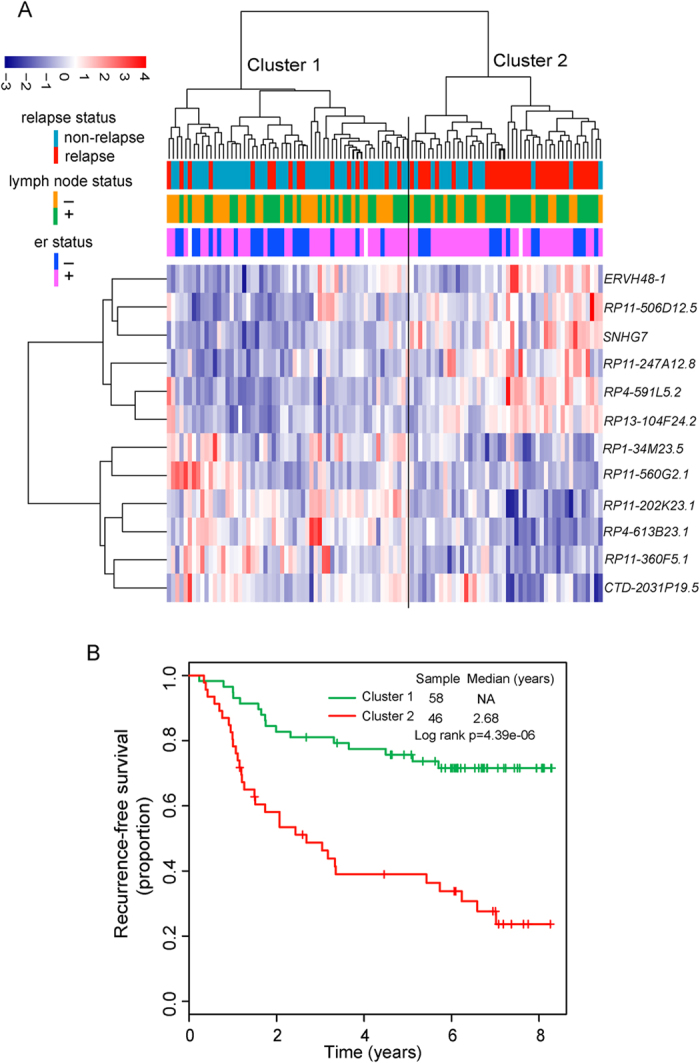
The heatmap and survival analysis of hierarchical clustering. (**A**) The hierarchical clustering heatmap of differentially expressed lncRNAs between relapsed and non-relapsed patients in the discovery cohort. (**B**) Kaplan-Meier survival curve for recurrence-free survival between two clusters.

To identify potential lncRNA biomarkers associated with tumor recurrence of breast cancer patients, the patients from discovery cohort were classified into two groups according to recurrence status. We first compared the lncRNA expression profiles of breast cancer patients who did and did not develop recurrence, and identified 12 differentially expressed lncRNAs between the two patient groups (p-value < 0.001 and FDR < 0.15) (see Supplementary Table S2). Among those lncRNAs, six lncRNAs were overexpressed and six were underexpressed in relapsed patients (see Supplementary Figure S1). Then we performed hierarchical clustering in 104 patients from discovery cohort based on the expression patterns of these 12 differentially expressed lncRNAs. As shown in Fig. 1A, the resulting dendrogram showed two main patient clusters (58 patients in cluster 1 vs. 46 patients in cluster 2), which were highly correlated with tumor recurrence status (p = 4.76e-05, Chi-square test) and lymph node status (p = 0.03, Chi-square test). Cluster 1 included 75% of non-relapsed patients while cluster 2 included 66.7% of relapsed patients. Moreover, a significant difference in recurrence-free survival between the two patient clusters was observed (log-rank test p = 4.39E-06; Fig. 1B). We further performed univariate Cox proportional hazard regression to each of 12 differentially expressed lncRNAs for their associations with recurrence-free survival. As shown in Table 1, all of them were closely correlated with patient’s recurrence-free survival in univariate analysis (p-value < 0.001 and FDR < 0.1). The above results demonstrated that these dysregulated lncRNAs were potential prognostic biomarkers for predicting the risk of tumor recurrence of breast cancer patients.

### Determination and analysis of a 12-lncRNA predictive signature in the discovery cohort

**Figure 2:**
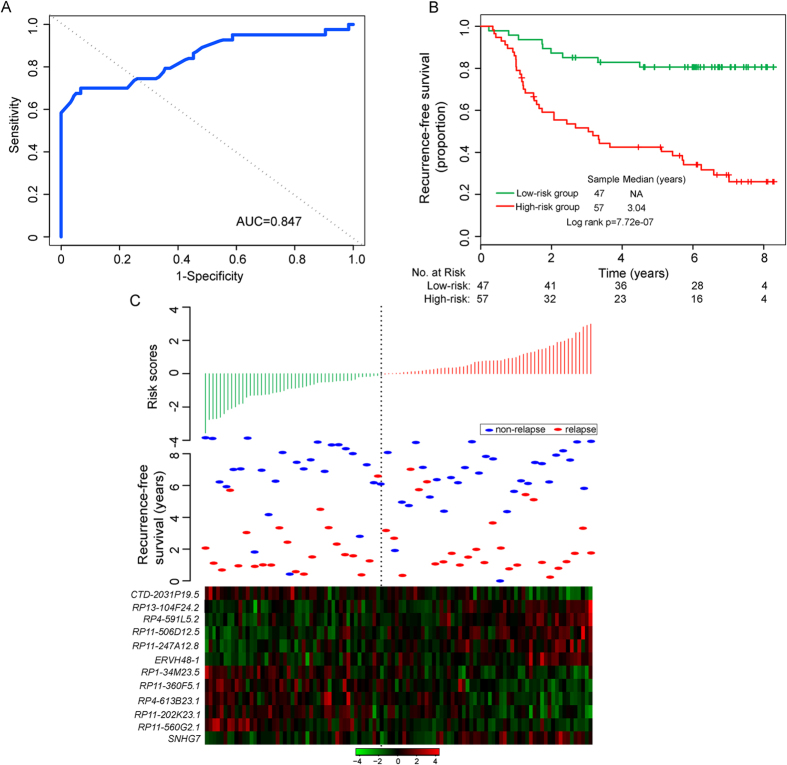
Development and analysis of the 12-lncRNA signature in the discovery cohort. (**A**) ROC analysis of the 12-lncRNA signature for recurrence risk prediction within five years as the defining point. (**B**) Kaplan–Meier survival curves of recurrence-free survival between high-risk and low-risk patients in the discovery cohort. (**C**) The distribution of patients’ risk score and recurrence status, and the expression pattern of prognostic lncRNAs.

Since these 12 differentially expressed lncRNAs exhibited distinct expression patterns in patients who did and did not develop recurrence, these 12 lncRNAs were integrated into a predictive signature by risk scoring method to predict the risk of tumor recurrence of breast cancer patients (hereafter inferred as BCSigLnc-12) (see Methods), as follows: BCSigLnc-12 risk score = (−0.4585 × expression value of *RP1-34M23.5*) + (−0.0009 × expression value of *RP11-202K23.1*) + (−0.3242 × expression value of *RP11-560G2.1*) + (0.1672 × expression value of *RP4-591L5.2*) + (−0.0134 × expression value of *RP13-104F24.2*) + (0.4971 × expression value of *RP11-506D12.5*) + (0.2189 × expression value of *ERVH48-1*) + (0.0137 × expression value of *RP4-613B23.1*) + (−0.3225 × expression value of *RP11-360F5.1*) + (−0.2464 × expression value of *CTD-2031P19.5*) + (0.2166 × expression value of *RP11-247A12.8*) + (−0.1720 × expression value of *SNHG7*). We calculated a BCSigLnc-12 risk score for each patient in the discovery cohort and ranked them according to increased risk score. To obtain the best cutoff value of the risk score, the various cutoff values were evaluated using time-dependent ROC curve^37^. In the discovery cohort, the time-dependent ROC curves analysis for the BCSigLnc-12 achieved an AUC of 0.847 at five years of recurrence-free survival (Fig. 2A), and the risk score value of −0.1, which produced the shortest distance to the point of perfect prediction of the five-year ROC curve, was selected as the cutoff point. According to this cutoff value, patients were classified into high-risk group and low-risk group. The patients with low-risk scores were expected to have better recurrence-free survival outcomes. As a result, the BCSigLnc-12 classified 104 patients of the discovery cohort into the high-risk group (n = 57) or low-risk group (n = 47). As expected, the recurrence-free survival time of patients in the high-risk group was significantly shorter than that of patients in the low-risk group (p = 7.72e-07, log-rank test) (Fig. 2B). The recurrence-free survival was 80.6% for patients with low-risk signatures at five years, which is higher than that (40.4%) for patients with high-risk signatures. Furthermore, different risk groups classified by BCSigLnc-12 were highly correlated with the tumor recurrence status (p = 1.44e-06, Chi-square test). There is a significant association between the BCSigLnc-12 risk score and recurrence-free survival time, in which the hazard ratio of high-risk group versus low-risk group for recurrence-free survival is 2.72 (95% confidence interval (CI) 2.07–3.57; p = 4.8e-13).

Distribution of BCSigLnc-12 risk scores, the relapse status and expression pattern of 12 lncRNA biomarkers of 104 breast patients in the discovery cohort was shown in Fig. 2C. Of these 12 lncRNA biomarkers, six were protective lncRNAs (*RP1-34M23.5*, *RP11-202K23.1*, *RP11-560G2.1*, *RP4-613B23.1*, *RP11-360F5.1* and *CTD-2031P19.5*) whose high expression was associated with good recurrence-free survival, while high expression of the remaining six (*RP4-591L5.2*, *RP13-104F24.2*, *RP11-506D12.5*, *ERVH48-1*, *RP11-247A12.8* and *SNHG7*) was associated with poor recurrence-free survival.

### Validation of BCSigLnc-12 in three additional independent test cohorts

**Figure 3:**
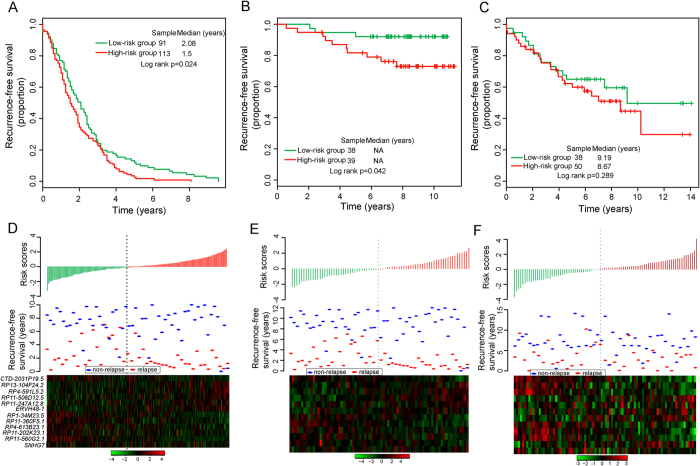
Validation of the prognostic value of the 12-lncRNA signature in the three additional independent cohorts. Kaplan-Meier survival curves of recurrence-free survival between high-risk and low-risk patients in the test cohort-1 (**A**), test cohort-2 (**B**) and test cohort-3 (**C**). The distribution of patients’ risk score and recurrence status, and the expression pattern of prognostic lncRNAs in the test cohort-1 (**D**), test cohort-2 (**E**) and test cohort-3 (**F**).

To evaluate the robustness of BCSigLnc-12 in predicting the risk of tumor recurrence of breast cancer patients, the BCSigLnc-12 was then tested for its predictive power in the test cohort-1 of 204 patients. With the same model and cutoff point as those derived from the discovery cohort, 204 patients of the test cohort-1 were classified into the high-risk group (n = 113) and low-risk group (n = 91). As in the discovery cohort, Kaplan-Meier recurrence-free survival curves based on BCSigLnc-12 prediction were significantly different. Patients in the high-risk group had significantly shorter recurrence-free survival than those in the low-risk group (median recurrence-free survival 1.5 years vs. 2.08 years, p = 0.024, log-rank test) (Fig. 3A). The five-year recurrence-free survival rate of the high-risk group was 2.7%, while the corresponding rate in the low-risk group was 12.1%. The hazard ratio of high-risk scores versus low-risk scores for recurrence-free survival was 1.20 (95% CI 1.03–1.39; p = 0.019). In the test cohort-1, the area under the time-dependent ROC curves was 0.7 at five years of recurrence-free survival.

Another validation of the predictive power of BCSigLnc-12 was conducted using independent test cohort from Loi’s study (termed test cohort-2). Similar to the findings from the discovery cohort and test cohort-1, the BCSigLnc-12 was again shown capable of predicting the risk of tumor recurrence in the test cohort-2. Using the risk score formula, the BCSigLnc-12 was able to stratify 77 patients of the test cohort-2 into the high-risk group (n = 39) and low-risk group (n = 38) with significantly different recurrence-free survival (log-rank p = 0.042) and relapse status (p = 0.046, Chi-square test) using the same cutoff as in the discovery cohort. The recurrence-free survival was significantly worse in the high-risk group compared with the low-risk group (Fig. 3B). At five years, the respective absolute difference in recurrence-free survival between the low-risk group and high-risk group was 10.4% (92.1% versus 81.7%). The hazard ratio of high-risk scores versus low-risk scores for recurrence-free survival was 1.93 (95% CI 1.14–3.29; p = 0.015). The AUC of time-dependent ROC curves for the BCSigLnc-12 in the test cohort-2 was 0.627 at five years.

Further validation of the BCSigLnc-12 in the test cohort-3 from Dedeurwaerder’s study showed that recurrence-free survival was different between the high-risk and low-risk groups (median recurrence-free survival 8.67 years vs. 9.19 years) and the proportions of recurrence-free survival in the high-risk and low-risk groups were 59.9% and 65% after five years. However, the p-value of the log-rank test is 0.289 indicating that the BCSigLnc-12 is not significantly associated with recurrence-free survival in the test cohort-3 (Fig. 3C).

The distribution of BCSigLnc-12 risk scores, relapse status and expression pattern of lncRNA biomarkers from breast cancer patients in the test cohort-1,test cohort-2 and test cohort-3 have been shown in Fig. 3D–F (ranked according to increasing risk scores), which were consistent with the results obtained from the discovery cohort except for test cohort-3.

### Independence of predictive capacity of BCSigLnc-12 from clinicopathological factors

We used multivariate Cox regression analysis to determine whether predictive capacity of BCSigLnc-12 was independent of other clinicopathological factors of breast cancer patients in the discovery cohort, test cohort-2 and test cohort-3 (no available clinicopathological information in test cohort-1), such as age, tumor size, tumor grade, estrogen receptor (ER) status and lymph node status. The results from the discovery cohort showed that the BCSigLnc-12 (HR = 2.81, 95% CI 1.97–4.01, p = 1.3e-08), ER status (HR = 0.3, 95% CI 0.15–0.6, p = 5.69e-04) and lymph node status (HR = 3.95, 95% CI 1.72–9.06, p = 0.001) were independent prognostic factors for patients with breast cancer (Table 2). In the test cohort-2, only the BCSigLnc-12 was significantly correlated with recurrence-free survival of patients with breast cancer in the multivariate analysis (HR = 1.85, 95% CI 1–3.42, p = 0.0498). However, the independence of predictive capacity of BCSigLnc-12 was not observed in the test cohort-3.

**Figure 4:**
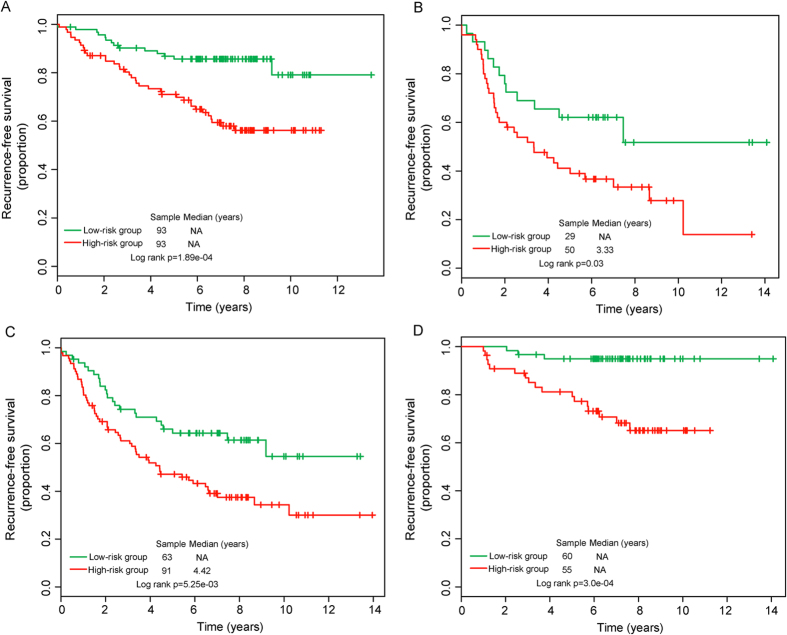
Survival analysis of patients with different ER and lymph node status classified into high-risk and low-risk groups based on the 12-lncRNA signature. Kaplan-Meier survival curves for ER+ patients (**A**) and ER− patients (**B**). Kaplan-Meier survival curves for patients with lymph node (**C**) and without lymph node (**D**).

We further performed data stratification analysis for breast cancer patients according to ER status and lymph node status. Patients with ER status information in four patient cohorts were first stratified into either the ER-positive group or the ER-negative group. Log-rank test of ER-positive patients showed that the BCSigLnc-12 could further classify ER-positive patients into the high-risk group (n = 93) and low-risk group (n = 93) with significantly different recurrence-free survival (p = 1.89e-04, log-rank test) (Fig. 4A). For ER-negative patients, the BCSigLnc-12 showed similar predictive value (p = 0.03, log-rank test) (Fig. 4B). Next, the stratified analysis was carried out in lymph node status, which stratified patients into lymph node-positive patient stratum and lymph node-negative patient stratum. Survival analysis showed that within each lymph node status stratum, the BCSigLnc-12 could subdivide patients into those likely to have poor recurrence-free survival and those likely to have good recurrence-free survival (p = 5.25e-03 for lymph node-positive patients and p = 3.0e-04 for lymph node-negative patients; log-rank test) (Fig. 4C,D). Taken together, the results of multivariate Cox regression analyses and stratification analysis suggested that the BCSigLnc-12 may be a risk predictor of tumor recurrence of breast cancer patients independent of clinicopathological factors which needed to be further validated.

### Functional prediction of prognostic lncRNA biomarkers

We performed functional enrichment analysis for GO terms and KEGG pathways to predict potential biological processes and pathways involved in relapsed-related lncRNA biomarkers. For this purpose, we first measured the co-expressed relationships between 12 relapse-related lncRNA biomarkers and protein-coding genes (PCGs) by calculating the Pearson correlation coefficient of paired lncRNA and PCG expression profiles, and identified highly positively or negatively correlated PCGs (ranked top 1%) with at least one of 12 relapse-related lncRNAs. The functional enrichment analysis of GO and KEGG pathway revealed that PCGs positively correlated with lncRNAs were involved in RNA metabolic process and spliceosome pathway, while PCGs negatively correlated with lncRNAs were enriched in five GO function clusters (including cell cycle, protein localization and protein catabolic process) and six pathways (including NOD-like receptor signaling pathway, glycosphingolipid biosynthesis, epithelial cell signaling in Helicobacter pylori infection, endocytosis, apoptosis and cell cycle). The BCSigLnc-12 associated biological processes and pathways can be found as Supplementary Table S3.

## Discussion

Although great efforts have been made to improve clinical management of breast cancer leading to a reduction in mortality rate, a substantial number of patients still faced the risk of tumor recurrence span 20 years^38^,^39^. As breast cancer is a heterogeneous disease at the clinicopathological and molecular levels, traditional prognostic factors, including stage, lymph node status, tumor size, tumor grade, lymphatic and vascular invasion, seem not to be sufficient for predicting the risk of tumor recurrence in breast cancer patients. With the development of microarray technology, gene expression profile-based multi-gene molecular signatures focusing on protein-coding genes or miRNAs have been identified and used to predict survival, metastasis and recurrence of tumor patients. LncRNAs, a recently discovered class of ncRNAs, have been implicated in the development and progression of cancer^40^,^41^,^42^. Although several lncRNAs have been linked to cancer recurrence, the expression pattern and prognostic value of lncRNAs in breast cancer recurrence have not been systematically investigated due to the lack of available lncRNA expression profiles. Recent studies found that some of microarray probes on the commonly used arrays are likely to map to lncRNAs^41^,^42^, representing a cost-effective way to obtain lncRNA expression profiles by repurposing microarray probes.

In this study, we analyzed and mined lncRNA expression profiles of breast cancer patients with recurrence information by repurposing the publicly available microarray expression profiles from GEO database to determine whether there are significantly different lncRNA expression patterns in breast patients with and without tumor recurrence. By first separating breast cancer patients into two groups according to relapse status, we identified 12 lncRNAs whose expression levels were significantly different between the two patient groups with and without recurrence. Hierarchical clustering and Cox regression analysis revealed that 12 differentially expressed lncRNAs were significantly correlated with tumor recurrence and recurrence-free survival of breast cancer patients. By focusing on these differentially expressed lncRNAs, we constructed a lncRNA expression-based molecular signature, termed BCSigLnc-12, which was able to accurately predict recurrence-free survival in breast cancer patient. Patients with high-risk signature scores have a 2.7-fold hazard ratio (95% CI 2.07–3.57; p < 0.001) for recurrence compared to those with low-risk signature scores. The predictive value and robustness of the BCSigLnc-12 were successfully validated in two out of three additional independent breast cancer patient cohorts. Notably, the BCSigLnc-12 clearly distinguished breast cancer patients with good recurrence-free survival for those with poor recurrence-free survival. Furthermore, when tested together with other clinical factors in multivariate Cox regression analysis, a certain degree of independence of predictive capacity of BCSigLnc-12 from clinicopathological factors was found, including age, tumor size, tumor grade, ER status and lymph node status. These results suggested that the BCSigLnc-12 may be candidate biomarkers for recurrence-free survival prediction in breast cancer.

More than ten thousand lncRNAs have been discovered in human during the past years. In contrast, the number of well-studied lncRNAs appears to be quite rare. LncRNA *SNHG7*, one of 12 relapse-related lncRNAs, is a known lincRNA belonging to endogenous retrovirus families and may play a role in trophoblasts syncytialization^43^. As studies on the mechanisms and function of lncRNAs are in their infancy, the computational prediction has been found to be effective in annotating lncRNA through the functional interpretation of their co-expressed mRNAs^44^. To gain the functional insight into these 12 prognostic lncRNAs, we performed GO and KEGG enrichment analysis for mRNAs co-expressed with lncRNAs, and found that the possible functions of 12 lncRNAs associated with tumor recurrence of breast cancer patients may be involved in cell cycle, spliceosome pathway apoptosis, NOD-like receptor signaling pathway, glycosphingolipid biosynthesis, epithelial cell signaling in Helicobacter pylori infection and endocytosis. Aberrant NOD-like receptor signal is shown to be a powerful driver of carcinogenesis^45^. Glycosphingolipids are involved in cell growth and motility, and their changes in expression, structure and organization promote tumor progression^46^. Several studies have suggested that the growth factor PGRN can promote wound healing migration of breast cancer cells^47^,^48^, and knockdown of PGRN has an impact on H. pylori-induced proliferative activity and migration of cancer cells^49^. There is evidence that endocytosis is involved in many physiological processes and plays important roles in human diseases, including cancer^50^. Thus it is a plausible inference that the 12 lncRNAs associated with recurrence may be involved in known breast cancer-related biological processes and pathways, and their dysregulated expression contributed to breast cancer recurrence.

However, some limitations should be aware in our study. First, only a fraction of lncRNAs was analyzed in our study because of limited available lncRNAs expression profiles. Second, only three of four independent cohorts confirmed the predictive value of this lncRNAs signature. Therefore, larger cohorts are needed to validate this signature. Finally, the biological implication of the BCSigLnc-12 was predicted using the bioinformatics analysis in our study and experimental studies need to be carried out to investigate the functional roles of the BCSigLnc-12 in breast cancer recurrence in the further work.

In conclusion, we have shown distinct expression patterns of lncRNAs in breast patients with relapse compared with those with relapse-free, and identified 12 differentially expressed lncRNAs that were closely associated with tumor recurrence of breast cancer patients. We therefore constructed a lncRNA-based molecular signature using the expression levels of these 12 relapsed-related lncRNAs that predicts the risk of tumor recurrence of breast cancer independently of clinicopathological factors, and validated it in two out of three independent breast cancer cohorts derived from different studies. Our study not only indicated the potential of lncRNAs as novel candidate biomarkers to provide additional recurrence risk stratification for breast cancer patients beyond the known clinical prognostic factors, but also improved our understanding of the molecular mechanism underlying breast cancer recurrence with further prospective validation.

## Methods

### Breast cancer patient cohorts

The gene expression profiles data generated by Affymetrix HG-U133 Plus 2.0 platform from four independent cohorts of breast cancer patients with relapse information were obtained from the publicly available GEO database. After removal of the patients without recurrence status, a total of 473 breast cancer patients were analyzed in this study (see Supplementary Table S1), including 104 patients from Clarke’s study (the GEO accession number is GSE42568) (http://www.ncbi.nlm.nih.gov/geo/query/acc.cgi?acc=GSE42568)^51^, 204 patients from Bos’s study (the GEO accession number is GSE12276) (http://www.ncbi.nlm.nih.gov/geo/query/acc.cgi?acc=GSE12276)^52^, 77 patients from Loi’s study (the GEO accession number is GSE9195) (http://www.ncbi.nlm.nih.gov/geo/query/acc.cgi?acc=GSE9195)^53^ and 88 patients from Dedeurwaerder’s study (the GEO accession number is GSE20711) (http://www.ncbi.nlm.nih.gov/geo/query/acc.cgi?acc=GSE20711)^54^.

### Acquisition and analysis of lncRNA expression profiles

The raw microarray data (.CEL files) of breast cancer patients in three cohorts were downloaded from the GEO database, and were background corrected, log2-transformed and normalized using the Robust Multichip Average (RMA) algorithm^55^ and the R package “Affy”^56^. The probe sequences of Affymetrix HG-U133 Plus 2.0 array were downloaded from the Affymetrix website (http://www.affymetrix.com) and re-mapped to the human genome (GRCh38) using SeqMap tool^57^. LncRNA-specific probes were obtained by matching the chromosomal position of probes to the chromosomal position of lncRNA genes based on the annotations from GENCODE (release 23) according to the previous studies^16^,^17^,^42^. After the removal of those probes that were mapped to multiple different lncRNAs, 2876 lncRNA-specific probes corresponding to 1938 lncRNAs were obtained for further analysis. When multiple probes were mapped to the same lncRNA, expression values of these probes were integrated by using the mean value to represent the expression value of the single lncRNAs.

The expression profiles of three cohorts were standardized by the Z-score transformation to avoid systematic error across different experiments^58^. Expression profiles of lncRNAs were analyzed using an unpaired two-tailed Student’s t-test and the p-values were adjusted for the effect of multiple tests using the Benjamini-Hochberg false discovery rate (FDR) control approach^59^ to determine differentially expressed lncRNAs in relapsed patients compared with non-relapsed patients. Hierarchical clustering of both patients and lncRNAs was performed with R software using the metric of euclidean distance and ward’s method.

### Statistical analysis

The Cox regression analysis was used to evaluate the association between expression levels of differentially expressed lncRNAs and patients’ recurrence-free survival. The selected differentially expressed lncRNAs were fitted in a multivariate Cox regression analysis in the discovery dataset. Then the lncRNA-focused molecular signature, termed BCSigLnc-12, was built using the linear combination of expression levels of prognostic lncRNAs with the estimated regression coefficients derived from the above multivariate Cox regression analysis as the weight to calculate the recurrence risk score for each patient as previously described^60^,^61^. The time-dependent receiver operating characteristic (ROC) curves were used to compare the sensitivity and specificity of the risk prediction of the BCSigLnc-12 for recurrence-free survival using the R package “survivalROC”^62^, and to identify the best patient stratification cutoff value in the discovery cohort. Patients were classified into high-risk and low-risk groups according to the above stratification cutoff. Kaplan-Meier survival analysis and log-rank test were used to compare the difference in recurrence-free survival between high-risk group and low-risk group using the R package “survival”. Multivariate analyses with Cox proportional hazards regression were performed to test whether the 12-lncRNA signature is independent of other clinicopathological factors with recurrence-free survival as the dependent variable and risk score and other clinical features as the explanatory variable in each cohort. Hazard ratio and 95% confidence intervals (CI) were estimated by Cox proportional hazards regression model. All statistical analyses were performed with R software.

### Functional enrichment analysis

We calculated the Pearson correlation coefficients between lncRNAs and protein-coding genes, and identified protein-coding genes positively or negatively correlated with prognostic lncRNAs. Functional enrichment analysis for these protein-coding genes was performed using DAVID Bioinformatics Tool (https://david.ncifcrf.gov/, version 6.7)^63^ limited to Gene Ontology (GO) terms in the “Biological Process” (GOTERM-BP-FAT) and Kyoto Encyclopedia of Genes and Genomes (KEGG) pathway categories. GO functional clusters with an enrichment score of >3.0 and KEGG pathway functional annotation with p-value of <0.01 using the whole human genome as background were considered as potential function of prognostic RNAs.

## Additional Information

**How to cite this article**: Zhou, M. *et al*. Discovery of potential prognostic long non-coding RNA biomarkers for predicting the risk of tumor recurrence of breast cancer patients. *Sci. Rep*. **6**, 31038; doi: 10.1038/srep31038 (2016).

## Supplementary Material

Supplementary Information

## Figures and Tables

**Table 1:** lncRNAs associated with tumor recurrence of breast cancer patients in the discovery cohort.

Ensembl ID	Probe ID	Gene name	Chromosome	*P-value*^a^	FDR	Hazard ratio^a^	Coefficient^a^
ENSG00000255811.1	216579_at, 243747_at	RP1-34M23.5	Chr 1: 34,761,426–34,788,097 (−)	4.44e-04	0.048	0.50	−0.55
ENSG00000233359.1	1566142_at, 216858_x_at, 201439_at, 224894_at, 202076_at, 1561543_at, 241072_s_at, 219086_at, 1554549_a_at, 239225_at, 227541_at.227693_at, 230223_at	RP11-202K23.1	Chr 1: 102,199,739–102,389,630 (−)	5.96e-04	0.052	0.62	−0.49
ENSG00000254451.2	224370_s_at	RP11-560G2.1	Chr 12: 75,234,740–75,298,508 (+)	6.47e-04	0.052	0.49	−0.71
ENSG00000231949.1	219781_s_at, 221968_s_at	RP4-591L5.2	Chr 1: 30,415,825–30,421,108 (+)	2.77e-05	0.018	1.75	0.56
ENSG00000215769.8	229747_x_at	RP13-104F24.2	Chr 17: 64,749,663–64,781,707 (−)	2.49e-05	0.018	1.71	0.53
ENSG00000261976.2	1554773_at	RP11-506D12.5	Chr 17: 50,840,057–50,841,626 (−)	5.85e-05	0.019	1.80	0.59
ENSG00000233056.2	232191_at	ERVH48-1	Chr 21: 42,916,803–42,925,646 (−)	1.73e-04	0.033	1.64	0.50
ENSG00000230084.5	231235_at, 202380_s_at, 1557736_at	RP4-613B23.1	Chr 3: 42,601,963–42,654,388 (−)	5.63e-04	0.052	0.57	−0.56
ENSG00000249207.1	226001_at, 232297_at, 233866_at	RP11-360F5.1	Chr 4: 39,112,677–39,126,818 (−)	5.16e-04	0.052	0.58	−0.55
ENSG00000262211.1	204864_s_at, 212195_at	CTD-2031P19.5	Chr 5: 55,936,143–55,941,727 (+)	6.90e-06	0.013	0.50	−0.67
ENSG00000268050.2	226559_at	RP11-247A12.8	Chr 9: 129,175,807–129,177,575 (+)	1.64e-04	0.033	1.72	0.54
ENSG00000233016.6	229002_at, 1552729_at	SNHG7	Chr 9: 136,721,366–136,728,184 (−)	8.67e-04	0.063	1.56	0.44

^a^Derived from the univariate Cox proportional hazard regression analysis in the discovery cohort.

**Table 2:** Multivariate Cox regression analysis of recurrence-free survival in each cohort.

Variables	Multivariate analysis		
	HR	95% CI of HR	***P*****-**value
**Discovery cohort, (n = 104)**			
BCSigLnc-12			
Low	1 (reference)		
High	2.81	1.97–4.01	1.3e-08
Age	1.15	0.60–2.20	0.683
ER			
ER−	1 (reference)		
ER+	0.30	0.15–0.60	5.69e-04
Grade			
G1	1 (reference)		
G2	0.73	0.16–3.46	0.694
G3	0.82	0.17–3.89	0.806
Size			
<=2 cm	1 (reference)		
>2 cm	0.72	0.31–1.69	0.447
Lymph node			
Lymph node−	1 (reference)		
Lymph node+	3.95	1.72–9.06	0.001
**Test cohort-2, (n = 77)**			
BCSigLnc-12			
Low	1 (reference)		
High	1.85	1.0004–3.42	0.0498
Age	0.85	0.26–2.75	0.786
Grade			
G1	1 (reference)		
G2	1.313	0.138–13.45	0.818
G3	1.314	0.137–13.61	0.819
Size			
<=2 cm	1 (reference)		
>2 cm	2.74	0.55–13.74	0.221
**Test cohort-3, (n = 88)**			
BCSigLnc-12			
Low	1 (reference)		
High	1.074	0.531–2.173	0.842
Grade			
G1	1 (reference)		
G2	1.271	0.179–9.046	0.811
G3	1.472	0.401–5.400	0.560
ER			
ER−	1 (reference)		
ER+	0.673	0.313–1.446	0.310
Lymph node			
Lymph node−	1 (reference)		
Lymph node+	2.553	1.104–5.903	0.028
Her2			
Her2−	1 (reference)		
Her2+	1.243	0.612–2.523	0.548

Abbreviations: HR, hazard ratio; CI, confidence interval.
