# Supplementary material for: Discovery of potential prognostic long non-coding RNA biomarkers for predicting the risk of tumor recurrence of breast cancer patients
Source: Sci Rep. 2016 Aug 9;6:31038. doi: 10.1038/srep31038 (PMC4977495; doi:10.1038/srep31038)
Supplement: Supplementary Information [file srep31038-s1.doc]

**Discovery of potential** **prognostic long non-coding RNA biomarkers for predicting the risk of tumor recurrence of breast cancer patients**

Meng Zhou^1, §^, Lei Zhong^2, §^, Wanying Xu^1, §^, Yifan Sun^1^, Zhaoyue Zhang^1^, Hengqiang Zhao^1^, Lei Yang^1^, Jie Sun ^1, *^

^1^College of Bioinformatics Science and Technology, Harbin Medical University, Harbin 150081, PR China

^2^Department of General Surgery, Second Affiliated Hospital of Harbin Medical University, Harbin Medical University, Harbin 150086, PR China

***Corresponding author**

Jie Sun, suncarajie@hotmail.com

College of Bioinformatics Science and Technology, Harbin Medical University, Harbin 150081, PR China

**^§^These authors contributed equally to this work**

**Supplementary Table S1.** Clinical and pathological characteristics of breast cancer patients with relapse information in our study.

| Covariates |  | Discovery cohort (GSE42568, n=104) | Test cohort-1  (GSE12276, n=204) | Test cohort-2  (GSE9195, n=77) | Test cohort-3  (GSE20711, n=88) |
| --- | --- | --- | --- | --- | --- |
| Age, no(%) | <=65 | 68(65.4) |  | 41(53.2) |  |
|  | >65 | 36(34.6) |  | 36(46.8) |  |
| Relapse status, no(%) | Relapse | 48(46.2) | 204(100) | 13(16.9) | 39 (44.3) |
|  | Not relapse | 56(53.8) |  | 64(83.1) | 49(55.7) |
| ER status, no(%) | N | 34(32.7) |  |  | 45 (51.1) |
|  | P | 67(64.4) |  | 77(100) | 42 (47.7) |
|  | NA | 3(2.9) |  |  | 1(1) |
| HER2 status, no(%) | N |  |  |  | 62 (70.5) |
|  | P |  |  |  | 26 (29.5) |
| Grade, no(%) |  |  |  |  |  |
|  | G1 | 11(10.6) |  | 14(18.2) | 13 (14.8) |
|  | G | 40(38.5) |  | 20(25.9) | 5 (5.7) |
|  | G3 | 53(50.9) |  | 24(31.2) | 70 (79.5) |
|  | NA |  |  | 19(24.7) |  |
| lymph node status, no(%) | N | 45(43.3) |  |  | 46 (52.3) |
|  | P | 59(56.7) |  |  | 42 (47.7) |
| Size, no(%) | >2cm | 69(66.3) |  | 43(55.8) |  |
|  | <=2cm | 35(33.7) |  | 34(44.2) |  |

**Supplementary Table S2.** Results of differential expression analysis for all lncRNAs between the two patient groups who did and did not develop recurrence

| lncRNA id | Gene name | Probe ID | logFC | mean exp (replase) | mean exp (nonreplase) | t-statistic | p-value | FDR |
| --- | --- | --- | --- | --- | --- | --- | --- | --- |
| ENSG00000255811.1 | RP1-34M23.5 | 216579_at,243747_at, | -0.06 | 4.05 | 4.21 | -3.64 | 4.34E-04 | 0.12 |
| ENSG00000254451.2 | RP11-560G2.1 | 224370_s_at, | -0.13 | 2.79 | 3.07 | -3.65 | 4.12E-04 | 0.12 |
| ENSG00000231949.1 | RP4-591L5.2 | 219781_s_at,221968_s_at, | 0.07 | 5.61 | 5.36 | 3.89 | 1.99E-04 | 0.12 |
| ENSG00000215769.8 | RP13-104F24.2 | 229747_x_at, | 0.10 | 4.18 | 3.91 | 3.89 | 1.89E-04 | 0.12 |
| ENSG00000261976.2 | RP11-506D12.5 | 1554773_at, | 0.06 | 3.91 | 3.75 | 3.54 | 6.33E-04 | 0.12 |
| ENSG00000233056.2 | ERVH48-1 | 232191_at, | 0.05 | 3.64 | 3.51 | 3.56 | 5.70E-04 | 0.12 |
| ENSG00000230084.5 | RP4-613B23.1 | 231235_at,202380_s_at,1557736_at, | -0.09 | 4.95 | 5.27 | -3.61 | 4.70E-04 | 0.12 |
| ENSG00000249207.1 | RP11-360F5.1 | 226001_at,232297_at,233866_at, | -0.12 | 4.49 | 4.89 | -3.71 | 3.44E-04 | 0.12 |
| ENSG00000262211.1 | CTD-2031P19.5 | 204864_s_at,212195_at, | -0.12 | 6.76 | 7.35 | -3.62 | 4.80E-04 | 0.12 |
| ENSG00000268050.2 | RP11-247A12.8 | 226559_at, | 0.12 | 6.45 | 5.94 | 3.59 | 5.30E-04 | 0.12 |
| ENSG00000233359.1 | RP11-202K23.1 | 1566142_at,216858_x_at,201439_at,224894_at,202076_at,1561543_at,  241072_s_at,219086_at,1554549_a_at,239225_at,227541_at,227693_at,  230223_at, | -0.03 | 5.88 | 5.99 | -3.48 | 7.84E-04 | 0.13 |
| ENSG00000233016.6 | SNHG7 | 229002_at,1552729_at, | 0.10 | 5.85 | 5.47 | 3.49 | 7.65E-04 | 0.13 |
| ENSG00000273637.1 | RP5-864K19.7 | 203359_s_at,214744_s_at,221860_at, | 0.09 | 6.02 | 5.64 | 3.41 | 1.05E-03 | 0.15 |
| ENSG00000254208.1 | RP11-219B4.3 | 231237_x_at, | 0.06 | 4.72 | 4.52 | 3.39 | 1.01E-03 | 0.15 |
| ENSG00000246477.3 | AF131216.6 | 204837_at, | -0.07 | 5.88 | 6.19 | -3.33 | 1.25E-03 | 0.16 |
| ENSG00000236423.5 | LINC01134 | 1569895_at, | 0.04 | 6.20 | 6.03 | 3.26 | 1.51E-03 | 0.16 |
| ENSG00000259943.1 | RP1-39G22.7 | 226457_at, | 0.06 | 4.92 | 4.73 | 3.20 | 1.93E-03 | 0.16 |
| ENSG00000226688.6 | ENTPD1-AS1 | 1560352_at,228585_at,243534_at, | -0.09 | 3.20 | 3.42 | -3.22 | 1.77E-03 | 0.16 |
| ENSG00000224505.2 | AC002117.1 | 202815_s_at,1557244_a_at, | -0.06 | 5.08 | 5.28 | -3.20 | 1.84E-03 | 0.16 |
| ENSG00000224090.1 | AC097468.4 | 239508_x_at,1557879_at, | 0.06 | 3.97 | 3.80 | 3.24 | 1.62E-03 | 0.16 |
| ENSG00000268858.2 | RP4-591C20.9 | 234002_at, | 0.06 | 3.98 | 3.81 | 3.20 | 1.91E-03 | 0.16 |
| ENSG00000246334.2 | PRR7-AS1 | 202848_s_at,210981_s_at, | 0.05 | 6.39 | 6.16 | 3.19 | 1.95E-03 | 0.16 |
| ENSG00000254615.2 | RP11-395G23.3 | 235205_at, | 0.14 | 4.70 | 4.27 | 3.27 | 1.56E-03 | 0.16 |
| ENSG00000232767.1 | RP11-498B4.5 | 214434_at, | -0.10 | 4.44 | 4.76 | -3.15 | 2.17E-03 | 0.18 |
| ENSG00000225302.2 | RP11-539I5.1 | 235800_at, | -0.14 | 4.68 | 5.15 | -3.12 | 2.41E-03 | 0.19 |
| ENSG00000256717.1 | AP000797.3 | 239675_at,239685_at,237935_at, | 0.06 | 3.45 | 3.31 | 3.10 | 2.64E-03 | 0.19 |
| ENSG00000257222.1 | RP11-554E23.4 | 238637_at, | -0.05 | 4.54 | 4.69 | -3.08 | 2.67E-03 | 0.19 |
| ENSG00000227619.1 | RP11-492E3.2 | 1569765_at, | -0.05 | 5.48 | 5.66 | -3.07 | 2.72E-03 | 0.19 |
| ENSG00000230623.2 | NA | 240681_at, | -0.06 | 3.27 | 3.41 | -2.88 | 4.84E-03 | 0.19 |
| ENSG00000234871.1 | LINC01032 | 234962_at, | 0.06 | 4.19 | 4.02 | 3.02 | 3.39E-03 | 0.19 |
| ENSG00000269621.1 | RP11-98D18.15 | 209609_s_at, | 0.07 | 9.32 | 8.90 | 2.89 | 4.85E-03 | 0.19 |
| ENSG00000234184.5 | RP5-887A10.1 | 1563849_at,216820_at,1562455_at,202986_at,1553647_at,227290_at, | -0.06 | 4.68 | 4.89 | -2.94 | 4.10E-03 | 0.19 |
| ENSG00000279696.1 | RP11-178H8.7 | 1565830_at, | 0.11 | 3.07 | 2.84 | 2.91 | 4.67E-03 | 0.19 |
| ENSG00000227589.1 | RP5-1092A11.5 | 1570480_s_at,240144_at,1557189_at,209154_at,1557052_at, | 0.03 | 4.98 | 4.87 | 2.93 | 4.21E-03 | 0.19 |
| ENSG00000255471.1 | RP11-736K20.5 | 243584_at, | -0.15 | 4.25 | 4.72 | -2.98 | 3.61E-03 | 0.19 |
| ENSG00000257449.1 | RP11-603J24.4 | 226759_at,229752_at, | -0.06 | 5.18 | 5.40 | -2.91 | 4.44E-03 | 0.19 |
| ENSG00000236333.3 | TRHDE-AS1 | 1560697_at,1560698_a_at, | -0.05 | 3.40 | 3.53 | -2.96 | 3.80E-03 | 0.19 |
| ENSG00000257342.1 | RP11-571M6.7 | 200714_x_at, | -0.04 | 8.17 | 8.40 | -2.98 | 3.59E-03 | 0.19 |
| ENSG00000231607.8 | DLEU2 | 1569142_at,1569600_at,229943_at,1556820_a_at,240288_at,1556821_x_at,  203659_s_at,239098_at,230192_at,205677_s_at, | -0.06 | 4.44 | 4.63 | -2.92 | 4.34E-03 | 0.19 |
| ENSG00000259735.1 | RP11-356M20.3 | 215408_at, | -0.05 | 3.18 | 3.29 | -2.91 | 4.60E-03 | 0.19 |
| ENSG00000279537.1 | RP11-133K1.8 | 1561320_at, | 0.05 | 4.15 | 3.99 | 3.03 | 3.16E-03 | 0.19 |
| ENSG00000260107.1 | AC005606.15 | 225104_at, | 0.08 | 5.73 | 5.41 | 3.03 | 3.17E-03 | 0.19 |
| ENSG00000267745.1 | RP11-686D22.8 | 236692_at, | -0.08 | 4.39 | 4.65 | -2.91 | 4.41E-03 | 0.19 |
| ENSG00000263585.1 | RP11-498C9.13 | 202148_s_at, | 0.06 | 8.04 | 7.72 | 2.88 | 4.85E-03 | 0.19 |
| ENSG00000265688.1 | MAFG-AS1 | 1559352_a_at, | 0.06 | 5.75 | 5.53 | 2.95 | 4.00E-03 | 0.19 |
| ENSG00000228262.8 | LINC01320 | 224836_at,227742_at, | -0.15 | 6.01 | 6.69 | -3.02 | 3.17E-03 | 0.19 |
| ENSG00000250906.1 | RP11-632F7.3 | 213419_at, | -0.13 | 5.62 | 6.15 | -3.02 | 3.21E-03 | 0.19 |
| ENSG00000247572.7 | CKMT2-AS1 | 242585_at,235443_at, | -0.09 | 4.56 | 4.84 | -2.88 | 4.90E-03 | 0.19 |
| ENSG00000262075.3 | DKFZP434A062 | 216608_at, | 0.06 | 4.27 | 4.11 | 2.90 | 4.61E-03 | 0.19 |
| ENSG00000224699.8 | LAMTOR5-AS1 | 205234_at,225554_s_at,1554659_at,225521_at, | -0.05 | 4.94 | 5.09 | -2.84 | 5.47E-03 | 0.21 |
| ENSG00000247121.6 | CTD-2260A17.2 | 1569521_s_at,1554272_at, | -0.05 | 3.18 | 3.29 | -2.84 | 5.47E-03 | 0.21 |
| ENSG00000255224.1 | CTD-3065J16.9 | 91684_g_at, | 0.09 | 6.77 | 6.37 | 2.82 | 6.01E-03 | 0.22 |
| ENSG00000186056.9 | MATN1-AS1 | 1557557_at,215053_at, | -0.04 | 3.21 | 3.30 | -2.79 | 6.27E-03 | 0.23 |
| ENSG00000280157.1 | RP11-520H14.7 | 1557869_at, | 0.05 | 2.83 | 2.73 | 2.81 | 6.17E-03 | 0.23 |
| ENSG00000247317.3 | RP11-273G15.2 | 1561850_at, | 0.06 | 5.16 | 4.96 | 2.79 | 6.39E-03 | 0.23 |
| ENSG00000280237.1 | MIR4697HG | 229734_at, | -0.09 | 4.34 | 4.61 | -2.78 | 6.58E-03 | 0.23 |
| ENSG00000224445.2 | NA | 219018_s_at, | 0.05 | 5.43 | 5.27 | 2.77 | 6.63E-03 | 0.23 |
| ENSG00000236856.1 | AC105393.1 | 218145_at, | 0.09 | 7.61 | 7.14 | 2.76 | 6.87E-03 | 0.23 |
| ENSG00000238290.1 | RP11-431K24.1 | 209603_at,209602_s_at,210737_at,209300_s_at,203734_at,1561817_at, | -0.07 | 6.48 | 6.83 | -2.68 | 8.62E-03 | 0.23 |
| ENSG00000255082.1 | GRM5-AS1 | 1565389_s_at, | 0.05 | 4.60 | 4.44 | 2.70 | 8.16E-03 | 0.23 |
| ENSG00000232298.2 | RP11-10N16.3 | 220130_x_at,225514_at,216388_s_at, | 0.04 | 5.05 | 4.91 | 2.69 | 8.38E-03 | 0.23 |
| ENSG00000234497.5 | ERICH3-AS1 | 218964_at,218564_at, | 0.04 | 5.52 | 5.39 | 2.70 | 8.23E-03 | 0.23 |
| ENSG00000261487.1 | AC135048.13 | 221864_at, | -0.06 | 7.26 | 7.57 | -2.71 | 8.00E-03 | 0.23 |
| ENSG00000278341.1 | RP5-1142A6.10 | 1561500_at, | 0.05 | 4.78 | 4.62 | 2.72 | 7.82E-03 | 0.23 |
| ENSG00000279428.1 | RP11-258F1.2 | 203268_s_at, | 0.04 | 6.12 | 5.95 | 2.70 | 8.15E-03 | 0.23 |
| ENSG00000267270.5 | PARD6G-AS1 | 224538_s_at, | 0.04 | 4.13 | 4.01 | 2.71 | 8.05E-03 | 0.23 |
| ENSG00000269352.1 | PTOV1-AS2 | 213690_s_at, | 0.05 | 5.62 | 5.42 | 2.68 | 8.73E-03 | 0.23 |
| ENSG00000279716.1 | AC006128.2 | 227691_at, | 0.05 | 3.78 | 3.65 | 2.70 | 8.08E-03 | 0.23 |
| ENSG00000267244.5 | CTB-31O20.4 | 230940_at, | 0.05 | 3.37 | 3.25 | 2.69 | 8.47E-03 | 0.23 |
| ENSG00000242540.2 | AC010729.1 | 204914_s_at,204915_s_at,204913_s_at, | 0.25 | 5.70 | 4.81 | 2.69 | 8.53E-03 | 0.23 |
| ENSG00000204380.4 | AC005042.4 | 240379_at,1563120_at, | 0.08 | 4.78 | 4.53 | 2.76 | 7.17E-03 | 0.23 |
| ENSG00000249252.5 | RP11-665G4.1 | 226939_at, | -0.12 | 7.33 | 7.95 | -2.68 | 8.84E-03 | 0.23 |
| ENSG00000251003.7 | ZFPM2-AS1 | 219778_at, | -0.12 | 4.50 | 4.88 | -2.68 | 8.55E-03 | 0.23 |
| ENSG00000264031.1 | ABHD15-AS1 | 1560306_at,1552301_a_at, | -0.20 | 4.29 | 4.91 | -2.65 | 9.51E-03 | 0.25 |
| ENSG00000267655.1 | CTD-2286N8.2 | 231515_at, | 0.05 | 3.52 | 3.41 | 2.64 | 9.65E-03 | 0.25 |
| ENSG00000269210.2 | RP11-173C1.1 | 1556244_s_at, | 0.18 | 3.11 | 2.75 | 2.68 | 9.56E-03 | 0.25 |
| ENSG00000233706.1 | RP5-1087E8.3 | 213595_s_at, | 0.06 | 5.38 | 5.14 | 2.63 | 1.01E-02 | 0.25 |
| ENSG00000203804.4 | ADAMTSL4-AS1 | 226071_at, | -0.07 | 3.27 | 3.43 | -2.63 | 9.92E-03 | 0.25 |
| ENSG00000259475.1 | RP11-654A16.3 | 214304_x_at,203627_at,225330_at, | -0.15 | 6.30 | 7.00 | -2.63 | 9.99E-03 | 0.25 |
| ENSG00000244513.6 | CTD-2013N24.2 | 227685_at,236829_at,213024_at, | -0.07 | 4.82 | 5.07 | -2.62 | 1.04E-02 | 0.25 |
| ENSG00000231616.8 | RP11-575L7.4 | 240547_at,232514_at, | -0.05 | 5.36 | 5.53 | -2.60 | 1.06E-02 | 0.25 |
| ENSG00000271893.1 | RP11-762E8.1 | 237992_at, | -0.05 | 2.60 | 2.69 | -2.60 | 1.09E-02 | 0.26 |
| ENSG00000233184.6 | RP11-421L21.3 | 1568598_at,217226_s_at,220974_x_at,228554_at,219441_s_at, | -0.08 | 5.68 | 6.02 | -2.55 | 1.24E-02 | 0.26 |
| ENSG00000257000.1 | RP13-820C6.2 | 212375_at,212376_s_at, | -0.04 | 6.39 | 6.57 | -2.55 | 1.26E-02 | 0.26 |
| ENSG00000260329.1 | RP11-412D9.4 | 239343_at, | -0.06 | 2.65 | 2.76 | -2.57 | 1.16E-02 | 0.26 |
| ENSG00000205861.11 | C1QTNF9B-AS1 | 222277_at, | -0.07 | 3.94 | 4.15 | -2.52 | 1.32E-02 | 0.26 |
| ENSG00000259116.1 | RP11-973N13.4 | 213376_at, | -0.09 | 6.22 | 6.61 | -2.53 | 1.32E-02 | 0.26 |
| ENSG00000262049.1 | RP13-1032I1.7 | 232460_at, | 0.04 | 4.72 | 4.58 | 2.52 | 1.35E-02 | 0.26 |
| ENSG00000266903.1 | CTB-171A8.1 | 212662_at, | 0.10 | 5.27 | 4.93 | 2.57 | 1.18E-02 | 0.26 |
| ENSG00000268983.1 | AC005253.4 | 200076_s_at, | 0.04 | 7.15 | 6.95 | 2.51 | 1.35E-02 | 0.26 |
| ENSG00000227227.1 | AC017101.10 | 202351_at, | -0.07 | 9.18 | 9.63 | -2.52 | 1.35E-02 | 0.26 |
| ENSG00000223647.1 | AL133249.1 | 210052_s_at, | 0.12 | 6.39 | 5.86 | 2.51 | 1.37E-02 | 0.26 |
| ENSG00000279322.1 | RP4-738P15.6 | 234946_at, | 0.08 | 4.48 | 4.23 | 2.58 | 1.16E-02 | 0.26 |
| ENSG00000237735.2 | AP000473.6 | 237528_at, | 0.04 | 2.45 | 2.39 | 2.54 | 1.25E-02 | 0.26 |
| ENSG00000261012.2 | RP11-116D2.1 | 218492_s_at, | 0.05 | 7.55 | 7.31 | 2.54 | 1.27E-02 | 0.26 |
| ENSG00000236869.1 | RP11-944L7.4 | 206314_at,242434_at,241231_at,233070_at,241714_at,1561361_at, | -0.03 | 3.62 | 3.71 | -2.53 | 1.32E-02 | 0.26 |
| ENSG00000228242.6 | AC093495.4 | 209375_at, | -0.04 | 6.90 | 7.12 | -2.58 | 1.15E-02 | 0.26 |
| ENSG00000250546.5 | RP11-8L2.1 | 1569812_at, | 0.04 | 2.54 | 2.47 | 2.56 | 1.24E-02 | 0.26 |
| ENSG00000250167.1 | CTC-321K16.1 | 222484_s_at, | -0.17 | 8.36 | 9.39 | -2.55 | 1.22E-02 | 0.26 |
| ENSG00000248881.1 | CTC-366B18.2 | 223261_at, | -0.10 | 4.01 | 4.29 | -2.54 | 1.26E-02 | 0.26 |
| ENSG00000184608.8 | FAM167A-AS1 | 226614_s_at, | -0.09 | 4.74 | 5.04 | -2.51 | 1.37E-02 | 0.26 |
| ENSG00000253675.1 | CTD-3118D11.2 | 1553155_x_at,1553153_at, | -0.05 | 4.23 | 4.40 | -2.56 | 1.21E-02 | 0.26 |
| ENSG00000228623.3 | ZNF883 | 207068_at,230876_at, | 0.11 | 3.50 | 3.23 | 2.53 | 1.35E-02 | 0.26 |
| ENSG00000276107.1 | CTD-2033D15.2 | 239336_at, | -0.15 | 4.32 | 4.80 | -2.49 | 1.44E-02 | 0.27 |
| ENSG00000231185.6 | AC005592.2 | 1552721_a_at, | -0.05 | 2.90 | 3.01 | -2.49 | 1.44E-02 | 0.27 |
| ENSG00000261771.5 | DYX1C1-CCPG1 | 221511_x_at, | -0.08 | 5.49 | 5.82 | -2.48 | 1.49E-02 | 0.27 |
| ENSG00000253859.2 | RP11-157I4.4 | 235978_at, | -0.22 | 4.41 | 5.12 | -2.48 | 1.49E-02 | 0.27 |
| ENSG00000180139.11 | ACTA2-AS1 | 1564257_at, | 0.04 | 4.11 | 3.99 | 2.47 | 1.51E-02 | 0.27 |
| ENSG00000248908.1 | RP11-730N24.1 | 1557009_a_at,1557008_at, | 0.04 | 3.97 | 3.87 | 2.47 | 1.55E-02 | 0.27 |
| ENSG00000260695.1 | RP11-513N24.1 | 1560872_at, | 0.04 | 3.14 | 3.05 | 2.45 | 1.59E-02 | 0.28 |
| ENSG00000226496.2 | LINC00323 | 240068_at, | 0.08 | 2.64 | 2.50 | 2.48 | 1.60E-02 | 0.28 |
| ENSG00000250619.1 | CTD-2215L10.1 | 213169_at, | -0.13 | 5.32 | 5.82 | -2.45 | 1.60E-02 | 0.28 |
| ENSG00000225087.1 | RP4-660H19.1 | 225320_at,203620_s_at,234757_at,214194_at,220980_s_at,  224455_s_at,242738_s_at,233752_s_at,208033_s_at,243323_s_at,1552904_at, | -0.02 | 4.72 | 4.80 | -2.44 | 1.65E-02 | 0.28 |
| ENSG00000267280.5 | TBX2-AS1 | 213417_at, | 0.06 | 5.09 | 4.86 | 2.44 | 1.67E-02 | 0.28 |
| ENSG00000228784.7 | LINC00954 | 210803_at, | 0.04 | 4.38 | 4.27 | 2.44 | 1.67E-02 | 0.28 |
| ENSG00000269956.1 | MKNK1-AS1 | 1564044_at,209467_s_at, | -0.03 | 5.68 | 5.81 | -2.42 | 1.72E-02 | 0.28 |
| ENSG00000227372.10 | TP73-AS1 | 213340_s_at,1564795_at, | -0.05 | 4.17 | 4.32 | -2.43 | 1.72E-02 | 0.28 |
| ENSG00000260966.1 | RP11-690D19.3 | 239425_at, | -0.08 | 3.82 | 4.04 | -2.41 | 1.76E-02 | 0.28 |
| ENSG00000237301.1 | RP4-680D5.2 | 208121_s_at,212736_at,202386_s_at, | -0.05 | 5.80 | 6.01 | -2.41 | 1.76E-02 | 0.28 |
| ENSG00000267125.2 | CTB-31O20.6 | 226328_at, | 0.03 | 5.79 | 5.65 | 2.41 | 1.76E-02 | 0.28 |
| ENSG00000241472.6 | PTPRG-AS1 | 232242_at,227126_at, | -0.07 | 3.26 | 3.42 | -2.43 | 1.71E-02 | 0.28 |
| ENSG00000227110.6 | LMCD1-AS1 | 218574_s_at,242767_at,227317_at, | -0.06 | 5.12 | 5.35 | -2.40 | 1.85E-02 | 0.29 |
| ENSG00000223949.6 | ROR1-AS1 | 1562823_at,231142_at,201716_at,216357_at,1559154_at,  213364_s_at,1557024_at,228879_at,235745_at, | -0.03 | 4.56 | 4.65 | -2.38 | 1.95E-02 | 0.30 |
| ENSG00000254873.1 | RP11-770J1.5 | 202038_at, | -0.05 | 8.49 | 8.80 | -2.38 | 1.99E-02 | 0.30 |
| ENSG00000259007.1 | RP11-463J10.3 | 224964_s_at, | -0.13 | 5.01 | 5.47 | -2.38 | 1.90E-02 | 0.30 |
| ENSG00000258949.1 | RP11-857B24.5 | 235369_at, | -0.08 | 5.44 | 5.75 | -2.38 | 1.92E-02 | 0.30 |
| ENSG00000230735.1 | RP11-413E1.4 | 205913_at,229660_at, | -0.08 | 5.44 | 5.74 | -2.36 | 2.00E-02 | 0.30 |
| ENSG00000267396.1 | RP11-845C23.3 | 236490_at, | 0.04 | 4.57 | 4.45 | 2.37 | 1.97E-02 | 0.30 |
| ENSG00000268095.1 | ZNF649-AS1 | 223616_at, | 0.07 | 3.27 | 3.11 | 2.38 | 1.99E-02 | 0.30 |
| ENSG00000237282.3 | LINC00851 | 231447_at, | 0.05 | 5.27 | 5.10 | 2.38 | 1.95E-02 | 0.30 |
| ENSG00000231200.1 | AC068490.2 | 1557483_at,240115_at,230789_at, | 0.10 | 3.12 | 2.92 | 2.38 | 1.97E-02 | 0.30 |
| ENSG00000271894.1 | RP11-482H16.1 | 235228_at,233846_at,204092_s_at,221442_at,224690_at, | 0.03 | 5.26 | 5.15 | 2.36 | 2.02E-02 | 0.30 |
| ENSG00000267394.1 | CTB-175E5.7 | 202692_s_at, | 0.05 | 6.73 | 6.52 | 2.36 | 2.04E-02 | 0.30 |
| ENSG00000256690.1 | RP11-727F15.9 | 240672_at, | 0.05 | 3.78 | 3.64 | 2.36 | 2.09E-02 | 0.30 |
| ENSG00000234264.1 | DEPDC1-AS1 | 1569958_at,240530_at,200605_s_at,242482_at, | -0.03 | 4.40 | 4.51 | -2.33 | 2.20E-02 | 0.30 |
| ENSG00000204971.3 | RP11-807H22.7 | 204437_s_at, | 0.22 | 4.96 | 4.25 | 2.34 | 2.18E-02 | 0.30 |
| ENSG00000246889.2 | AP000487.5 | 210236_at,202066_at, | 0.08 | 6.57 | 6.21 | 2.35 | 2.12E-02 | 0.30 |
| ENSG00000230798.5 | FOXD3-AS1 | 214164_x_at, | -0.15 | 8.13 | 9.00 | -2.34 | 2.16E-02 | 0.30 |
| ENSG00000260618.1 | RP11-23N2.4 | 225327_at, | -0.12 | 6.78 | 7.37 | -2.34 | 2.16E-02 | 0.30 |
| ENSG00000259952.1 | AC009133.15 | 229807_s_at, | 0.05 | 3.96 | 3.84 | 2.33 | 2.19E-02 | 0.30 |
| ENSG00000250295.6 | RDH10-AS1 | 227467_at, | 0.13 | 5.82 | 5.32 | 2.34 | 2.20E-02 | 0.30 |
| ENSG00000228237.5 | EFCAB14-AS1 | 1559022_at,201778_s_at,241669_x_at, | -0.04 | 5.56 | 5.73 | -2.32 | 2.24E-02 | 0.31 |
| ENSG00000261845.2 | RP13-638C3.4 | 223408_s_at, | 0.06 | 5.72 | 5.49 | 2.32 | 2.26E-02 | 0.31 |
| ENSG00000270087.5 | RP11-399K21.11 | 228684_at, | 0.04 | 3.67 | 3.56 | 2.31 | 2.32E-02 | 0.31 |
| ENSG00000235016.1 | SEMA3F-AS1 | 201395_at, | -0.04 | 8.92 | 9.18 | -2.31 | 2.32E-02 | 0.31 |
| ENSG00000228363.2 | AC015971.2 | 202636_at, | -0.05 | 8.35 | 8.64 | -2.30 | 2.37E-02 | 0.31 |
| ENSG00000225285.1 | RP4-758J18.10 | 238639_x_at, | 0.04 | 5.29 | 5.14 | 2.30 | 2.42E-02 | 0.32 |
| ENSG00000242349.5 | NPPA-AS1 | 203950_s_at, | -0.05 | 5.13 | 5.29 | -2.28 | 2.47E-02 | 0.32 |
| ENSG00000228889.6 | UBAC2-AS1 | 1557038_s_at,1557037_a_at, | -0.04 | 3.62 | 3.72 | -2.28 | 2.49E-02 | 0.32 |
| ENSG00000259972.2 | AC009120.6 | 237518_at, | 0.03 | 5.39 | 5.26 | 2.28 | 2.48E-02 | 0.32 |
| ENSG00000267225.1 | WDR7-OT1 | 237122_at,212880_at, | -0.05 | 4.50 | 4.64 | -2.28 | 2.50E-02 | 0.32 |
| ENSG00000197210.7 | KB-1592A4.15 | 232736_s_at, | 0.05 | 4.37 | 4.22 | 2.28 | 2.49E-02 | 0.32 |
| ENSG00000242908.6 | AADACL2-AS1 | 223939_at, | -0.09 | 3.48 | 3.71 | -2.28 | 2.55E-02 | 0.32 |
| ENSG00000230223.6 | ATXN8OS | 216391_s_at, | 0.04 | 3.91 | 3.80 | 2.26 | 2.60E-02 | 0.33 |
| ENSG00000270135.1 | RP11-362K14.7 | 236918_s_at,236654_s_at, | -0.06 | 2.73 | 2.85 | -2.27 | 2.60E-02 | 0.33 |
| ENSG00000253445.1 | CTB-79E8.2 | 225355_at, | -0.07 | 7.19 | 7.55 | -2.25 | 2.63E-02 | 0.33 |
| ENSG00000232335.1 | RP11-435D7.3 | 237216_at, | -0.09 | 4.42 | 4.70 | -2.25 | 2.69E-02 | 0.33 |
| ENSG00000256802.2 | RP11-680F8.1 | 1557405_at, | -0.04 | 2.95 | 3.03 | -2.25 | 2.69E-02 | 0.33 |
| ENSG00000277969.1 | CTB-58E17.1 | 227571_at, | 0.08 | 4.89 | 4.61 | 2.26 | 2.70E-02 | 0.33 |
| ENSG00000259080.1 | RP11-158I13.2 | 220321_s_at,1553218_a_at,225050_at,1561938_at, | -0.04 | 4.61 | 4.73 | -2.24 | 2.72E-02 | 0.33 |
| ENSG00000267222.1 | RP11-194N12.2 | 1559374_at, | -0.03 | 3.00 | 3.07 | -2.24 | 2.74E-02 | 0.33 |
| ENSG00000225721.5 | RP11-269F19.2 | 209408_at, | 0.09 | 6.93 | 6.49 | 2.24 | 2.77E-02 | 0.33 |
| ENSG00000259673.5 | IQCH-AS1 | 220361_at,238485_at, | -0.04 | 4.26 | 4.39 | -2.23 | 2.79E-02 | 0.33 |
| ENSG00000261734.1 | RP11-669C19.1 | 234189_at, | 0.05 | 2.71 | 2.61 | 2.23 | 2.80E-02 | 0.33 |
| ENSG00000223745.7 | RP4-717I23.3 | 228465_at,242894_at,244383_at,201798_s_at, | -0.05 | 6.03 | 6.26 | -2.22 | 2.89E-02 | 0.33 |
| ENSG00000225506.2 | CYP4A22-AS1 | 217319_x_at,203257_s_at,227191_at,241226_at,217879_at,  217880_at,223155_at, | -0.03 | 5.31 | 5.41 | -2.21 | 2.99E-02 | 0.33 |
| ENSG00000258121.1 | RP11-722P11.4 | 240353_s_at, | 0.04 | 2.88 | 2.79 | 2.20 | 2.99E-02 | 0.33 |
| ENSG00000204603.6 | LINC01257 | 234493_at, | -0.04 | 2.52 | 2.58 | -2.20 | 2.98E-02 | 0.33 |
| ENSG00000235706.7 | DICER1-AS1 | 1557063_at, | 0.04 | 2.78 | 2.70 | 2.22 | 2.89E-02 | 0.33 |
| ENSG00000258407.1 | RP11-300J18.2 | 214467_at, | -0.10 | 3.98 | 4.25 | -2.22 | 2.90E-02 | 0.33 |
| ENSG00000188825.13 | LINC00910 | 1570049_at,1559144_x_at, | -0.04 | 2.89 | 2.97 | -2.20 | 2.99E-02 | 0.33 |
| ENSG00000142396.10 | NA | 242139_s_at, | -0.07 | 7.10 | 7.47 | -2.22 | 2.91E-02 | 0.33 |
| ENSG00000223947.1 | AC016738.4 | 205459_s_at,213462_at,39549_at, | 0.08 | 5.36 | 5.09 | 2.21 | 2.94E-02 | 0.33 |
| ENSG00000233806.7 | LINC01237 | 1556473_at,241505_at, | -0.16 | 5.82 | 6.49 | -2.22 | 2.89E-02 | 0.33 |
| ENSG00000230612.3 | AC004237.1 | 228680_at, | -0.07 | 6.28 | 6.58 | -2.22 | 2.87E-02 | 0.33 |
| ENSG00000230490.2 | RP11-141M1.3 | 1563596_at, | -0.05 | 3.36 | 3.48 | -2.19 | 3.06E-02 | 0.34 |
| ENSG00000225762.1 | LINC01389 | 204816_s_at, | 0.04 | 3.16 | 3.07 | 2.20 | 3.10E-02 | 0.34 |
| ENSG00000237667.5 | LINC01115 | 1562844_at,216505_x_at, | 0.03 | 5.74 | 5.62 | 2.19 | 3.08E-02 | 0.34 |
| ENSG00000177640.15 | CASC2 | 1564372_s_at,1564371_a_at, | -0.03 | 2.76 | 2.82 | -2.18 | 3.17E-02 | 0.34 |
| ENSG00000257453.1 | RP11-290L1.3 | 218000_s_at, | 0.05 | 4.52 | 4.37 | 2.17 | 3.23E-02 | 0.34 |
| ENSG00000259915.2 | RP11-410E4.1 | 233826_at, | -0.03 | 2.66 | 2.73 | -2.18 | 3.21E-02 | 0.34 |
| ENSG00000279390.1 | AF127577.13 | 223951_at, | -0.06 | 3.21 | 3.35 | -2.18 | 3.17E-02 | 0.34 |
| ENSG00000269984.1 | RP11-362K14.5 | 1566108_at,1566109_at, | -0.03 | 2.70 | 2.76 | -2.17 | 3.22E-02 | 0.34 |
| ENSG00000251260.1 | WDFY3-AS1 | 238660_at, | -0.06 | 4.04 | 4.20 | -2.17 | 3.22E-02 | 0.34 |
| ENSG00000272338.2 | RP11-722E23.2 | 237419_at, | -0.06 | 4.04 | 4.20 | -2.18 | 3.15E-02 | 0.34 |
| ENSG00000266677.1 | RP11-258F1.1 | 228078_at,1570071_at,228034_x_at, | 0.03 | 4.12 | 4.03 | 2.17 | 3.26E-02 | 0.34 |
| ENSG00000244738.1 | RP11-373E16.3 | 1562352_at,1562353_x_at, | 0.04 | 2.68 | 2.60 | 2.17 | 3.28E-02 | 0.34 |
| ENSG00000233338.1 | TLR8-AS1 | 1562805_at, | 0.04 | 2.89 | 2.81 | 2.17 | 3.30E-02 | 0.34 |
| ENSG00000232811.1 | RP11-96K19.2 | 220235_s_at, | -0.12 | 3.96 | 4.32 | -2.16 | 3.35E-02 | 0.34 |
| ENSG00000223814.1 | RP11-543D5.2 | 220574_at, | -0.10 | 4.66 | 5.00 | -2.16 | 3.36E-02 | 0.34 |
| ENSG00000262001.1 | DLGAP1-AS2 | 223974_at, | -0.04 | 3.71 | 3.82 | -2.15 | 3.36E-02 | 0.34 |
| ENSG00000279434.1 | RP11-85K15.3 | 1569894_at, | -0.04 | 2.94 | 3.02 | -2.15 | 3.45E-02 | 0.35 |
| ENSG00000254604.1 | AP000487.6 | 242990_at, | 0.10 | 5.75 | 5.35 | 2.14 | 3.55E-02 | 0.35 |
| ENSG00000227082.1 | CH17-437K3.1 | 229855_at,219035_s_at,226215_s_at, | -0.04 | 5.31 | 5.46 | -2.13 | 3.55E-02 | 0.35 |
| ENSG00000261574.1 | RP1-168P16.2 | 228866_at, | 0.06 | 6.77 | 6.50 | 2.13 | 3.53E-02 | 0.35 |
| ENSG00000280029.3 | CH17-140K24.2 | 216313_at,216355_at,221450_x_at,221317_x_at,208504_x_at,  223629_at,234479_at,223854_at,234724_x_at,221408_x_at, | 0.03 | 4.88 | 4.77 | 2.14 | 3.50E-02 | 0.35 |
| ENSG00000260196.1 | RP1-239B22.5 | 229715_at, | -0.10 | 3.72 | 3.98 | -2.12 | 3.62E-02 | 0.35 |
| ENSG00000279965.1 | RP11-472K17.3 | 1556476_at,1556477_a_at, | 0.04 | 3.09 | 3.00 | 2.13 | 3.64E-02 | 0.35 |
| ENSG00000260742.1 | RP11-366L5.1 | 1561215_at, | -0.05 | 2.56 | 2.64 | -2.13 | 3.62E-02 | 0.35 |
| ENSG00000225376.5 | TMEM246-AS1 | 224458_at, | 0.04 | 4.24 | 4.12 | 2.12 | 3.64E-02 | 0.35 |
| ENSG00000227857.2 | RP4-533D7.5 | 238774_at, | -0.10 | 5.46 | 5.85 | -2.11 | 3.72E-02 | 0.36 |
| ENSG00000248489.1 | CTD-2007H13.3 | 238456_at, | -0.06 | 3.94 | 4.11 | -2.11 | 3.72E-02 | 0.36 |
| ENSG00000257500.1 | RP11-1020M18.10 | 207065_at, | 0.04 | 4.66 | 4.53 | 2.10 | 3.82E-02 | 0.36 |
| ENSG00000260807.6 | RP11-161M6.2 | 1569871_at,1558463_s_at, | 0.04 | 5.16 | 5.00 | 2.11 | 3.81E-02 | 0.36 |
| ENSG00000246731.2 | MGC16275 | 1558167_a_at,1558166_at, | 0.04 | 3.43 | 3.34 | 2.11 | 3.78E-02 | 0.36 |
| ENSG00000233621.1 | LINC01137 | 229678_at, | -0.06 | 4.54 | 4.74 | -2.09 | 3.88E-02 | 0.36 |
| ENSG00000264589.1 | NA | 231253_at,237331_s_at, | 0.03 | 4.51 | 4.41 | 2.10 | 3.88E-02 | 0.36 |
| ENSG00000254813.5 | RP11-252C15.1 | 1562924_at, | 0.05 | 3.96 | 3.83 | 2.10 | 3.87E-02 | 0.36 |
| ENSG00000266978.1 | CTD-2369P2.5 | 223742_at, | 0.06 | 5.10 | 4.89 | 2.09 | 3.96E-02 | 0.37 |
| ENSG00000272933.1 | RP11-47A8.5 | 231161_x_at, | 0.05 | 5.60 | 5.42 | 2.09 | 3.98E-02 | 0.37 |
| ENSG00000261675.1 | RP5-1119A7.17 | 244099_at, | 0.04 | 3.68 | 3.58 | 2.07 | 4.06E-02 | 0.37 |
| ENSG00000281100.1 | RP11-640L9.2 | 233476_at, | -0.03 | 5.85 | 5.97 | -2.08 | 4.05E-02 | 0.37 |
| ENSG00000253875.1 | RP11-16P20.3 | 218125_s_at, | -0.05 | 6.07 | 6.27 | -2.08 | 4.06E-02 | 0.37 |
| ENSG00000218537.1 | MIF-AS1 | 1556316_s_at, | 0.06 | 6.53 | 6.28 | 2.07 | 4.11E-02 | 0.37 |
| ENSG00000260475.1 | RP11-85A1.3 | 1556970_at, | 0.04 | 4.16 | 4.04 | 2.07 | 4.15E-02 | 0.37 |
| ENSG00000280268.1 | RP11-147K16.3 | 1558667_at,1558668_s_at, | 0.03 | 2.51 | 2.47 | 2.07 | 4.14E-02 | 0.37 |
| ENSG00000213963.6 | AC074286.1 | 231540_at, | -0.04 | 3.17 | 3.25 | -2.06 | 4.17E-02 | 0.37 |
| ENSG00000198468.7 | FLVCR1-AS1 | 235126_at, | -0.06 | 5.70 | 5.94 | -2.06 | 4.19E-02 | 0.37 |
| ENSG00000241014.1 | RP11-244H3.1 | 236246_x_at,220608_s_at, | 0.03 | 4.75 | 4.64 | 2.05 | 4.39E-02 | 0.37 |
| ENSG00000224870.6 | RP4-758J18.2 | 225920_at, | 0.03 | 5.80 | 5.67 | 2.04 | 4.37E-02 | 0.37 |
| ENSG00000276255.2 | RP5-881P19.7 | 221109_at, | 0.03 | 5.00 | 4.89 | 2.04 | 4.45E-02 | 0.37 |
| ENSG00000226304.1 | RP11-435M3.2 | 1563771_a_at, | -0.04 | 2.54 | 2.61 | -2.06 | 4.22E-02 | 0.37 |
| ENSG00000255435.6 | RP11-770J1.3 | 244301_at,212076_at,226981_at,212080_at, | -0.03 | 5.78 | 5.92 | -2.06 | 4.25E-02 | 0.37 |
| ENSG00000268531.3 | RP11-32B5.8 | 234891_at, | 0.04 | 4.63 | 4.50 | 2.05 | 4.35E-02 | 0.37 |
| ENSG00000263072.5 | NA | 206416_at, | 0.05 | 6.15 | 5.95 | 2.05 | 4.26E-02 | 0.37 |
| ENSG00000266368.1 | RP11-1096G20.5 | 207959_s_at, | 0.04 | 3.99 | 3.89 | 2.05 | 4.35E-02 | 0.37 |
| ENSG00000235643.1 | RP1-69M21.2 | 1555209_at,240857_at, | -0.06 | 2.74 | 2.86 | -2.06 | 4.28E-02 | 0.37 |
| ENSG00000263146.2 | RP11-849I19.1 | 236643_s_at, | 0.05 | 3.13 | 3.02 | 2.04 | 4.44E-02 | 0.37 |
| ENSG00000203395.2 | AC015969.3 | 1562902_at, | -0.05 | 2.49 | 2.57 | -2.04 | 4.41E-02 | 0.37 |
| ENSG00000234936.1 | AC010883.5 | 217346_at, | 0.03 | 6.00 | 5.88 | 2.04 | 4.43E-02 | 0.37 |
| ENSG00000280262.1 | RP11-542P2.2 | 244649_at, | -0.05 | 2.99 | 3.09 | -2.04 | 4.41E-02 | 0.37 |
| ENSG00000259426.5 | RP11-253M7.1 | 243622_at, | 0.04 | 5.10 | 4.98 | 2.03 | 4.54E-02 | 0.38 |
| ENSG00000261924.1 | CTD-2561B21.5 | 1565903_at, | 0.04 | 4.43 | 4.31 | 2.03 | 4.55E-02 | 0.38 |
| ENSG00000238197.5 | PAXBP1-AS1 | 239407_at, | -0.05 | 3.87 | 4.00 | -2.03 | 4.52E-02 | 0.38 |
| ENSG00000253133.1 | RP11-360L9.4 | 1554356_at, | 0.05 | 3.22 | 3.11 | 2.03 | 4.53E-02 | 0.38 |
| ENSG00000267458.1 | CTC-425F1.4 | 212952_at, | 0.03 | 10.30 | 10.08 | 2.02 | 4.62E-02 | 0.38 |
| ENSG00000225733.5 | FGD5-AS1 | 226985_at,217300_at, | -0.04 | 4.97 | 5.12 | -2.01 | 4.70E-02 | 0.38 |
| ENSG00000240666.2 | MME-AS1 | 203434_s_at, | -0.16 | 3.50 | 3.90 | -2.01 | 4.71E-02 | 0.38 |
| ENSG00000229348.1 | HYI-AS1 | 240447_at,1558345_a_at, | -0.07 | 3.55 | 3.72 | -2.01 | 4.76E-02 | 0.38 |
| ENSG00000237750.2 | AC007740.1 | 237608_at, | -0.03 | 2.41 | 2.47 | -2.01 | 4.75E-02 | 0.38 |
| ENSG00000226664.1 | RP4-745E8.2 | 214324_at,228900_at, | -0.16 | 4.97 | 5.55 | -2.00 | 4.84E-02 | 0.39 |
| ENSG00000253490.5 | AC145110.1 | 1569099_at, | -0.05 | 3.18 | 3.30 | -1.99 | 4.91E-02 | 0.39 |
| ENSG00000223711.1 | AC091633.3 | 241235_at, | 0.03 | 2.48 | 2.44 | 1.99 | 4.96E-02 | 0.40 |
| ENSG00000228436.2 | RP5-864K19.4 | 203359_s_at,1563339_at,1554895_a_at,230964_at,228388_at,220605_s_at, | 0.03 | 4.33 | 4.25 | 1.99 | 4.99E-02 | 0.40 |
| ENSG00000255467.1 | RP11-144G7.2 | 203531_at, | -0.06 | 8.00 | 8.32 | -1.99 | 5.04E-02 | 0.40 |
| ENSG00000260304.1 | RP11-388M20.6 | 1565717_s_at, | 0.07 | 5.18 | 4.94 | 1.98 | 5.06E-02 | 0.40 |
| ENSG00000237424.1 | FOXD2-AS1 | 224457_at,224456_s_at, | 0.04 | 3.53 | 3.43 | 1.96 | 5.23E-02 | 0.40 |
| ENSG00000230021.7 | NA | 240305_at,1557548_at,234952_s_at,236886_at,224886_at,1562799_at,  1554466_a_at,223911_at,227625_s_at,227269_s_at,227698_s_at,  1559109_a_at,213645_at,217690_at,202932_at,217684_at,204142_at,  1561375_at,228989_at,214019_at,1566816_at,207592_s_at,241921_x_at, | 0.02 | 5.61 | 5.55 | 1.96 | 5.25E-02 | 0.40 |
| ENSG00000272106.1 | RP11-345P4.9 | 213732_at, | 0.04 | 3.50 | 3.39 | 1.97 | 5.18E-02 | 0.40 |
| ENSG00000273118.1 | AC079610.1 | 1555060_a_at, | 0.05 | 3.78 | 3.65 | 1.96 | 5.25E-02 | 0.40 |
| ENSG00000213981.8 | AC007277.3 | 1552578_a_at, | -0.11 | 2.82 | 3.04 | -1.98 | 5.20E-02 | 0.40 |
| ENSG00000226785.1 | AC073218.1 | 1565611_at,236235_at,1562316_at,236878_at,1559614_at,215253_s_at,  208370_s_at,217337_at, | 0.03 | 4.36 | 4.28 | 1.97 | 5.14E-02 | 0.40 |
| ENSG00000254262.1 | RP11-58O3.2 | 244283_x_at, | 0.03 | 2.38 | 2.34 | 1.98 | 5.15E-02 | 0.40 |
| ENSG00000236756.4 | DNAJC9-AS1 | 229701_at,229170_s_at, | 0.04 | 3.36 | 3.27 | 1.96 | 5.36E-02 | 0.40 |
| ENSG00000267707.2 | RP11-95O2.5 | 243665_s_at,223654_s_at, | 0.06 | 3.51 | 3.36 | 1.96 | 5.34E-02 | 0.40 |
| ENSG00000269813.1 | CTD-3193O13.14 | 227746_at, | 0.08 | 3.73 | 3.53 | 1.97 | 5.30E-02 | 0.40 |
| ENSG00000250802.6 | ZBED3-AS1 | 1564475_s_at,1564474_at, | -0.03 | 2.95 | 3.02 | -1.96 | 5.32E-02 | 0.40 |
| ENSG00000249349.1 | CTC-384G19.1 | 1563396_x_at, | -0.05 | 2.27 | 2.35 | -1.96 | 5.34E-02 | 0.40 |
| ENSG00000255202.1 | RP4-541C22.5 | 244584_at, | 0.04 | 3.07 | 2.99 | 1.94 | 5.55E-02 | 0.41 |
| ENSG00000231163.5 | CSMD2-AS1 | 231049_at,220568_at, | -0.07 | 2.99 | 3.15 | -1.95 | 5.50E-02 | 0.41 |
| ENSG00000257759.2 | RP11-486O13.4 | 1552579_a_at, | -0.05 | 3.18 | 3.30 | -1.95 | 5.44E-02 | 0.41 |
| ENSG00000258520.1 | RP11-363J20.2 | 218363_at, | -0.04 | 6.66 | 6.84 | -1.95 | 5.45E-02 | 0.41 |
| ENSG00000232451.1 | AC016768.1 | 1566923_at,1566924_at,231089_at,202877_s_at,203887_s_at,  203888_at,237252_at, | -0.04 | 4.42 | 4.53 | -1.94 | 5.47E-02 | 0.41 |
| ENSG00000203644.3 | RP11-332M2.1 | 227356_at, | -0.08 | 6.03 | 6.35 | -1.94 | 5.55E-02 | 0.41 |
| ENSG00000240423.1 | LINC00636 | 1560514_at, | 0.03 | 4.22 | 4.14 | 1.94 | 5.51E-02 | 0.41 |
| ENSG00000282143.1 | RP1-163M9.8 | 239496_at,1569543_at, | 0.04 | 3.93 | 3.82 | 1.93 | 5.61E-02 | 0.41 |
| ENSG00000243389.1 | AC012442.5 | 237993_at, | 0.04 | 4.97 | 4.83 | 1.93 | 5.63E-02 | 0.41 |
| ENSG00000258908.1 | RP11-203M5.8 | 201695_s_at, | 0.10 | 6.32 | 5.91 | 1.93 | 5.69E-02 | 0.41 |
| ENSG00000249532.3 | RP11-148B6.1 | 215246_at, | -0.04 | 3.74 | 3.85 | -1.92 | 5.72E-02 | 0.41 |
| ENSG00000262691.1 | CTC-277H1.7 | 1559092_at, | 0.03 | 4.16 | 4.08 | 1.93 | 5.75E-02 | 0.41 |
| ENSG00000230023.2 | RP11-10N16.2 | 1562716_at,218571_s_at, | -0.03 | 6.16 | 6.31 | -1.89 | 6.16E-02 | 0.42 |
| ENSG00000224066.1 | RP4-622L5.7 | 219585_at, | 0.04 | 3.46 | 3.37 | 1.90 | 6.04E-02 | 0.42 |
| ENSG00000238022.1 | RP11-38C18.3 | 240534_at, | 0.04 | 3.80 | 3.70 | 1.90 | 6.03E-02 | 0.42 |
| ENSG00000246250.2 | RP11-613D13.5 | 217869_at, | 0.04 | 9.46 | 9.21 | 1.92 | 5.84E-02 | 0.42 |
| ENSG00000257379.1 | RP11-793H13.8 | 217794_at,237813_at, | -0.02 | 5.94 | 6.04 | -1.88 | 6.24E-02 | 0.42 |
| ENSG00000261351.2 | CTD-3185P2.1 | 202670_at, | -0.06 | 6.66 | 6.95 | -1.90 | 5.97E-02 | 0.42 |
| ENSG00000260988.1 | RP11-285A1.1 | 1560772_a_at, | 0.03 | 3.20 | 3.14 | 1.90 | 5.98E-02 | 0.42 |
| ENSG00000262152.6 | LA16c-380H5.1 | 1564122_at, | 0.05 | 3.39 | 3.28 | 1.90 | 6.20E-02 | 0.42 |
| ENSG00000261312.1 | AC002550.5 | 213235_at, | 0.05 | 8.31 | 8.04 | 1.90 | 6.03E-02 | 0.42 |
| ENSG00000227036.6 | LINC00511 | 236993_at,230812_at, | 0.03 | 4.76 | 4.65 | 1.91 | 5.87E-02 | 0.42 |
| ENSG00000266101.1 | RP5-906A24.2 | 210185_at, | 0.05 | 5.23 | 5.07 | 1.89 | 6.22E-02 | 0.42 |
| ENSG00000266002.1 | RP11-567L7.5 | 227020_at, | -0.06 | 6.63 | 6.93 | -1.88 | 6.26E-02 | 0.42 |
| ENSG00000267165.1 | RP11-78A19.3 | 218178_s_at, | 0.03 | 5.60 | 5.49 | 1.89 | 6.16E-02 | 0.42 |
| ENSG00000236144.6 | TMEM147-AS1 | 1558381_a_at, | 0.03 | 3.88 | 3.79 | 1.91 | 5.90E-02 | 0.42 |
| ENSG00000224043.7 | CCNT2-AS1 | 242046_at, | -0.04 | 3.05 | 3.13 | -1.90 | 6.01E-02 | 0.42 |
| ENSG00000273451.1 | RP4-569M23.4 | 230533_at, | -0.05 | 5.88 | 6.09 | -1.89 | 6.12E-02 | 0.42 |
| ENSG00000234617.1 | SNRK-AS1 | 209481_at, | -0.06 | 7.02 | 7.31 | -1.89 | 6.19E-02 | 0.42 |
| ENSG00000261757.1 | AC005592.3 | 234509_at, | -0.07 | 2.98 | 3.13 | -1.89 | 6.20E-02 | 0.42 |
| ENSG00000250025.2 | CTC-359M8.1 | 208554_at, | 0.04 | 2.96 | 2.88 | 1.91 | 5.91E-02 | 0.42 |
| ENSG00000251314.2 | CTD-2337A12.1 | 205825_at, | -0.25 | 2.88 | 3.43 | -1.90 | 6.25E-02 | 0.42 |
| ENSG00000232725.1 | U52111.14 | 205142_x_at, | 0.05 | 5.30 | 5.12 | 1.88 | 6.25E-02 | 0.42 |
| ENSG00000182109.7 | RP11-69E11.4 | 220204_s_at,225694_at,225697_at,225691_at,225690_at,225936_at, | 0.05 | 6.01 | 5.79 | 1.88 | 6.40E-02 | 0.42 |
| ENSG00000228470.1 | RP11-176D17.3 | 226282_at, | 0.07 | 7.49 | 7.13 | 1.88 | 6.38E-02 | 0.42 |
| ENSG00000261316.1 | CTC-400I9.1 | 1569305_a_at, | 0.02 | 5.36 | 5.29 | 1.88 | 6.36E-02 | 0.42 |
| ENSG00000274629.1 | RP11-92F20.1 | 201370_s_at, | -0.08 | 5.07 | 5.36 | -1.88 | 6.40E-02 | 0.42 |
| ENSG00000237481.1 | RP4-803J11.2 | 225904_at, | 0.07 | 7.11 | 6.78 | 1.87 | 6.45E-02 | 0.42 |
| ENSG00000226779.1 | NAALADL2-AS2 | 241163_at, | -0.07 | 3.37 | 3.55 | -1.87 | 6.48E-02 | 0.42 |
| ENSG00000259775.1 | RP11-45P15.4 | 1556216_s_at, | -0.07 | 2.72 | 2.85 | -1.86 | 6.53E-02 | 0.42 |
| ENSG00000229956.9 | NA | 239548_at,243357_at,239136_at,1565908_at,228915_at,205472_s_at,  239600_at,231652_at,229287_at,1565557_at,1562524_at,234612_at,  228958_at,222811_at,1566502_at,213934_s_at, | -0.04 | 4.78 | 4.90 | -1.85 | 6.69E-02 | 0.43 |
| ENSG00000260570.1 | RP11-24N18.1 | 201806_s_at, | 0.04 | 5.60 | 5.46 | 1.85 | 6.71E-02 | 0.43 |
| ENSG00000271646.1 | RP11-326I11.3 | 235543_at, | -0.05 | 5.67 | 5.85 | -1.85 | 6.74E-02 | 0.43 |
| ENSG00000250241.5 | RP11-9G1.3 | 1562389_at, | -0.05 | 2.50 | 2.58 | -1.85 | 6.79E-02 | 0.43 |
| ENSG00000259968.3 | RP11-428C6.2 | 1564749_at, | 0.05 | 3.59 | 3.47 | 1.85 | 6.78E-02 | 0.43 |
| ENSG00000279151.1 | RP1-163M9.7 | 1560643_x_at,1560642_at, | 0.04 | 3.11 | 3.02 | 1.85 | 6.82E-02 | 0.43 |
| ENSG00000269145.2 | AC007192.6 | 213045_at, | -0.04 | 6.12 | 6.29 | -1.84 | 6.84E-02 | 0.43 |
| ENSG00000238133.6 | MLK7-AS1 | 1555259_at,222757_s_at,225665_at,237133_at,223519_at, | -0.05 | 5.57 | 5.76 | -1.84 | 6.86E-02 | 0.43 |
| ENSG00000231987.1 | RP5-898J17.1 | 1566363_at,1558569_at,221773_at,219164_s_at,1564471_at,209120_at, | -0.03 | 5.98 | 6.12 | -1.83 | 7.02E-02 | 0.44 |
| ENSG00000236088.9 | COX10-AS1 | 1557192_at, | -0.07 | 3.82 | 4.02 | -1.82 | 7.18E-02 | 0.45 |
| ENSG00000267857.2 | RP5-1023B21.1 | 231346_s_at,231245_s_at, | 0.03 | 3.69 | 3.60 | 1.82 | 7.24E-02 | 0.45 |
| ENSG00000250337.5 | LINC01021 | 1557765_at, | -0.07 | 2.35 | 2.47 | -1.83 | 7.24E-02 | 0.45 |
| ENSG00000279347.1 | RP11-85I17.2 | 1552961_at, | 0.04 | 3.21 | 3.13 | 1.82 | 7.19E-02 | 0.45 |
| ENSG00000239205.1 | RP11-747D18.1 | 1564323_at, | -0.05 | 2.54 | 2.63 | -1.81 | 7.32E-02 | 0.45 |
| ENSG00000224356.5 | RP11-151A6.4 | 1556502_at, | 0.04 | 3.50 | 3.41 | 1.81 | 7.37E-02 | 0.46 |
| ENSG00000214184.3 | GCC2-AS1 | 202832_at, | -0.06 | 6.54 | 6.82 | -1.81 | 7.41E-02 | 0.46 |
| ENSG00000253917.4 | AC226119.5 | 241341_at, | 0.04 | 3.60 | 3.52 | 1.81 | 7.41E-02 | 0.46 |
| ENSG00000234425.1 | RP11-528G1.2 | 230391_at,211191_at, | -0.05 | 4.98 | 5.15 | -1.80 | 7.50E-02 | 0.46 |
| ENSG00000203321.2 | C9orf41-AS1 | 243646_at, | -0.07 | 4.50 | 4.71 | -1.79 | 7.58E-02 | 0.46 |
| ENSG00000229951.5 | FLJ31356 | 1560932_at, | 0.03 | 2.77 | 2.72 | 1.79 | 7.66E-02 | 0.47 |
| ENSG00000255850.1 | TMEM5-AS1 | 204807_at, | -0.08 | 5.52 | 5.86 | -1.79 | 7.73E-02 | 0.47 |
| ENSG00000261338.2 | RP11-378A13.1 | 239064_at, | -0.04 | 4.95 | 5.09 | -1.79 | 7.72E-02 | 0.47 |
| ENSG00000233399.2 | RP11-111I12.1 | 237394_at,1564429_at,227781_x_at,210157_at, | 0.02 | 4.90 | 4.82 | 1.78 | 7.79E-02 | 0.47 |
| ENSG00000245832.6 | MIR4300HG | 208904_s_at, | 0.01 | 12.64 | 12.52 | 1.78 | 7.78E-02 | 0.47 |
| ENSG00000240618.1 | NA | 237389_at,209092_s_at,212925_at, | -0.03 | 7.28 | 7.43 | -1.78 | 7.84E-02 | 0.47 |
| ENSG00000224209.6 | NA | 1556622_s_at,243481_at,238906_s_at,219210_s_at,226633_at,  226358_at,237837_at, | -0.03 | 4.30 | 4.40 | -1.77 | 7.92E-02 | 0.47 |
| ENSG00000280120.1 | RP11-546D6.3 | 215731_s_at, | -0.08 | 4.11 | 4.36 | -1.77 | 8.01E-02 | 0.48 |
| ENSG00000260810.1 | CTD-2547L24.4 | 1556779_s_at, | 0.03 | 3.58 | 3.50 | 1.76 | 8.13E-02 | 0.48 |
| ENSG00000260252.1 | RP11-384M15.3 | 218794_s_at, | 0.04 | 6.20 | 6.04 | 1.76 | 8.18E-02 | 0.48 |
| ENSG00000230148.8 | HOXB-AS1 | 208414_s_at,237189_at,228904_at, | -0.09 | 6.17 | 6.59 | -1.76 | 8.16E-02 | 0.48 |
| ENSG00000259018.1 | RP11-124D2.3 | 204400_at, | 0.10 | 7.39 | 6.89 | 1.76 | 8.26E-02 | 0.49 |
| ENSG00000214772.2 | RP11-174G6.1 | 1556253_s_at, | -0.12 | 3.65 | 3.95 | -1.75 | 8.34E-02 | 0.49 |
| ENSG00000260317.1 | RP11-48B3.4 | 230302_at, | 0.09 | 4.35 | 4.09 | 1.75 | 8.35E-02 | 0.49 |
| ENSG00000224939.1 | LINC00184 | 1559646_a_at,1559645_at, | 0.03 | 3.27 | 3.21 | 1.72 | 8.87E-02 | 0.49 |
| ENSG00000236656.1 | RP11-144L1.4 | 1556944_at, | 0.04 | 2.59 | 2.52 | 1.72 | 8.82E-02 | 0.49 |
| ENSG00000275678.1 | RP4-547N15.3 | 242459_at, | 0.04 | 3.30 | 3.21 | 1.73 | 8.72E-02 | 0.49 |
| ENSG00000175147.11 | TMEM51-AS1 | 213752_at, | 0.04 | 4.49 | 4.36 | 1.74 | 8.47E-02 | 0.49 |
| ENSG00000247473.2 | CARS-AS1 | 1562436_at, | 0.03 | 3.47 | 3.40 | 1.74 | 8.49E-02 | 0.49 |
| ENSG00000255422.1 | AP002954.4 | 1562836_at, | 0.04 | 5.93 | 5.77 | 1.73 | 8.66E-02 | 0.49 |
| ENSG00000255517.6 | CTD-3074O7.5 | 244214_at,236997_at, | 0.03 | 4.93 | 4.83 | 1.72 | 8.87E-02 | 0.49 |
| ENSG00000257545.4 | RP11-144F15.1 | 223673_at,237260_at,219459_at, | -0.03 | 4.73 | 4.82 | -1.72 | 8.90E-02 | 0.49 |
| ENSG00000277011.1 | RP13-895J2.6 | 1560782_at, | 0.04 | 5.05 | 4.90 | 1.73 | 8.61E-02 | 0.49 |
| ENSG00000281344.1 | HELLPAR | 1561543_at,241072_s_at,209541_at, | -0.13 | 5.04 | 5.53 | -1.72 | 8.83E-02 | 0.49 |
| ENSG00000235423.8 | RP11-282O18.3 | 201938_at, | 0.03 | 10.60 | 10.36 | 1.72 | 8.92E-02 | 0.49 |
| ENSG00000258301.3 | RP11-488C13.5 | 203940_s_at, | -0.05 | 6.22 | 6.42 | -1.72 | 8.79E-02 | 0.49 |
| ENSG00000258844.1 | RP11-259K15.2 | 237355_at, | 0.03 | 4.63 | 4.52 | 1.73 | 8.67E-02 | 0.49 |
| ENSG00000279980.1 | GABARAPL3 | 211457_at,211458_s_at, | 0.03 | 6.12 | 6.01 | 1.74 | 8.47E-02 | 0.49 |
| ENSG00000259895.1 | RP11-715J22.2 | 1563637_at, | -0.03 | 2.43 | 2.48 | -1.74 | 8.56E-02 | 0.49 |
| ENSG00000265749.5 | RP11-849F2.5 | 1558533_at, | -0.03 | 4.79 | 4.90 | -1.74 | 8.54E-02 | 0.49 |
| ENSG00000267104.2 | TBC1D3P1-DHX40P1 | 221194_s_at, | -0.03 | 3.79 | 3.89 | -1.72 | 8.94E-02 | 0.49 |
| ENSG00000230615.6 | RP5-1198O20.4 | 221759_at,206175_x_at,239462_at,207128_s_at, | -0.03 | 4.92 | 5.02 | -1.73 | 8.75E-02 | 0.49 |
| ENSG00000267191.1 | RP11-15A1.2 | 222028_at, | -0.06 | 6.19 | 6.44 | -1.73 | 8.74E-02 | 0.49 |
| ENSG00000224875.2 | AC083949.1 | 212931_at, | 0.02 | 7.79 | 7.65 | 1.74 | 8.59E-02 | 0.49 |
| ENSG00000241684.5 | ADAMTS9-AS2 | 1562295_at,234078_at, | -0.03 | 3.23 | 3.30 | -1.75 | 8.39E-02 | 0.49 |
| ENSG00000253744.1 | AC025442.3 | 242184_s_at, | 0.04 | 3.90 | 3.79 | 1.74 | 8.55E-02 | 0.49 |
| ENSG00000226530.1 | RP11-348F1.2 | 1555396_s_at, | -0.04 | 3.09 | 3.17 | -1.72 | 8.93E-02 | 0.49 |
| ENSG00000270605.1 | RP5-1092A3.4 | 1557383_a_at, | -0.11 | 3.87 | 4.17 | -1.71 | 9.02E-02 | 0.49 |
| ENSG00000227375.5 | DLG1-AS1 | 1556518_at, | -0.03 | 2.76 | 2.81 | -1.71 | 9.02E-02 | 0.49 |
| ENSG00000267466.1 | RP11-13K12.5 | 1562764_at,1559017_at, | 0.03 | 3.56 | 3.49 | 1.71 | 9.06E-02 | 0.49 |
| ENSG00000255240.5 | RP11-142C4.6 | 227794_at,1563867_at, | -0.06 | 4.39 | 4.59 | -1.70 | 9.16E-02 | 0.49 |
| ENSG00000259153.1 | RP6-65G23.3 | 1565836_at, | -0.03 | 3.21 | 3.29 | -1.70 | 9.14E-02 | 0.49 |
| ENSG00000262251.1 | RP11-199F11.2 | 224185_at, | 0.07 | 5.14 | 4.89 | 1.70 | 9.21E-02 | 0.49 |
| ENSG00000262580.5 | RP11-334C17.5 | 1555308_at, | 0.03 | 4.34 | 4.24 | 1.70 | 9.21E-02 | 0.49 |
| ENSG00000228504.1 | RP4-586O15.1 | 218087_s_at, | -0.12 | 5.31 | 5.78 | -1.69 | 9.39E-02 | 0.50 |
| ENSG00000257526.2 | RP11-20E24.1 | 241061_at, | -0.02 | 2.44 | 2.47 | -1.69 | 9.50E-02 | 0.50 |
| ENSG00000257894.2 | RP1-78O14.1 | 203998_s_at,203999_at, | -0.16 | 3.47 | 3.88 | -1.69 | 9.38E-02 | 0.50 |
| ENSG00000236107.7 | AC010127.3 | 1555246_a_at, | -0.05 | 3.00 | 3.10 | -1.68 | 9.52E-02 | 0.50 |
| ENSG00000279160.1 | RP11-387A1.6 | 205975_s_at, | -0.04 | 3.91 | 4.01 | -1.68 | 9.56E-02 | 0.50 |
| ENSG00000260583.1 | AP000223.42 | 1556414_at, | 0.04 | 3.02 | 2.93 | 1.69 | 9.47E-02 | 0.50 |
| ENSG00000243305.1 | RP11-362A9.3 | 201153_s_at,201151_s_at,201152_s_at, | -0.04 | 6.71 | 6.89 | -1.69 | 9.43E-02 | 0.50 |
| ENSG00000245293.2 | RP11-286E11.1 | 226402_at,226393_at,243141_at, | -0.03 | 4.69 | 4.78 | -1.69 | 9.40E-02 | 0.50 |
| ENSG00000247311.2 | CTC-441N14.2 | 1556907_at, | 0.03 | 3.69 | 3.61 | 1.68 | 9.57E-02 | 0.50 |
| ENSG00000253535.5 | RP11-624C23.1 | 241446_at,211239_s_at, | -0.04 | 2.94 | 3.02 | -1.70 | 9.32E-02 | 0.50 |
| ENSG00000281649.1 | EBLN3 | 226635_at,225640_at, | -0.04 | 8.00 | 8.24 | -1.68 | 9.53E-02 | 0.50 |
| ENSG00000279863.1 | RP11-521C22.2 | 220670_at, | -0.03 | 2.87 | 2.94 | -1.67 | 9.74E-02 | 0.50 |
| ENSG00000230439.2 | RP11-488P3.1 | 1559623_at,223268_at,229851_s_at, | -0.04 | 5.77 | 5.92 | -1.67 | 9.87E-02 | 0.50 |
| ENSG00000279879.1 | RP11-1072C15.6 | 1564075_a_at, | 0.03 | 3.14 | 3.08 | 1.67 | 9.76E-02 | 0.50 |
| ENSG00000224789.1 | AC012363.4 | 225855_at, | -0.07 | 5.44 | 5.72 | -1.68 | 9.69E-02 | 0.50 |
| ENSG00000260837.1 | RP11-434B12.1 | 236166_at, | -0.07 | 3.78 | 3.95 | -1.68 | 9.71E-02 | 0.50 |
| ENSG00000279519.1 | RP11-288C18.1 | 244354_at, | 0.04 | 5.80 | 5.63 | 1.67 | 9.84E-02 | 0.50 |
| ENSG00000245864.2 | CTC-467M3.1 | 209200_at,230902_at,209199_s_at,1561392_at, | -0.06 | 6.47 | 6.76 | -1.68 | 9.63E-02 | 0.50 |
| ENSG00000177738.3 | CTD-2201E18.3 | 227901_at, | 0.03 | 5.45 | 5.33 | 1.67 | 9.81E-02 | 0.50 |
| ENSG00000247993.2 | FOXD1-AS1 | 206307_s_at, | 0.13 | 4.43 | 4.06 | 1.68 | 9.74E-02 | 0.50 |
| ENSG00000253608.1 | RP11-770E5.1 | 1563993_at, | -0.04 | 3.16 | 3.26 | -1.67 | 9.88E-02 | 0.50 |
| ENSG00000229851.1 | ARSD-AS1 | 223696_at,230131_x_at,223695_s_at, | -0.05 | 6.46 | 6.70 | -1.67 | 9.87E-02 | 0.50 |
| ENSG00000229444.1 | RP11-184I16.4 | 214392_at, | 0.03 | 3.50 | 3.43 | 1.67 | 9.95E-02 | 0.50 |
| ENSG00000198491.3 | RP11-208N14.4 | 1553823_a_at,1553822_at, | 0.03 | 2.74 | 2.69 | 1.67 | 9.95E-02 | 0.50 |
| ENSG00000279589.1 | RP11-454F8.4 | 233740_at, | 0.03 | 2.91 | 2.85 | 1.65 | 1.02E-01 | 0.51 |
| ENSG00000268532.1 | RP11-514D23.1 | 1562623_at, | 0.05 | 2.96 | 2.86 | 1.66 | 1.01E-01 | 0.51 |
| ENSG00000223442.1 | TH2LCRR | 209349_at, | 0.05 | 3.53 | 3.41 | 1.65 | 1.02E-01 | 0.51 |
| ENSG00000232063.1 | RP11-307E17.8 | 1559514_at, | -0.03 | 2.75 | 2.81 | -1.65 | 1.01E-01 | 0.51 |
| ENSG00000261488.1 | RP11-757F18.5 | 1556220_at, | -0.07 | 2.60 | 2.73 | -1.64 | 1.04E-01 | 0.52 |
| ENSG00000198685.3 | LINC01565 | 208440_at, | 0.04 | 4.83 | 4.71 | 1.64 | 1.04E-01 | 0.52 |
| ENSG00000251169.2 | AC005355.2 | 1568643_a_at, | 0.05 | 3.83 | 3.71 | 1.64 | 1.05E-01 | 0.52 |
| ENSG00000259172.1 | RP11-299G20.2 | 207414_s_at, | -0.10 | 5.59 | 6.01 | -1.63 | 1.05E-01 | 0.52 |
| ENSG00000235910.1 | APOA1-AS | 213034_at,204156_at, | -0.05 | 4.78 | 4.94 | -1.63 | 1.06E-01 | 0.52 |
| ENSG00000264449.5 | RP11-945C19.4 | 238128_at, | 0.03 | 3.07 | 3.00 | 1.63 | 1.06E-01 | 0.52 |
| ENSG00000245937.7 | LINC01184 | 231171_at,228686_at,230685_at, | -0.04 | 4.65 | 4.79 | -1.63 | 1.06E-01 | 0.52 |
| ENSG00000260793.2 | RP5-882C2.2 | 230580_at, | -0.04 | 4.60 | 4.73 | -1.63 | 1.07E-01 | 0.52 |
| ENSG00000260759.1 | RP11-677O4.2 | 1561223_at, | -0.03 | 3.62 | 3.71 | -1.63 | 1.07E-01 | 0.52 |
| ENSG00000230424.1 | RP1-43E13.2 | 215991_s_at,1556186_s_at,1556597_a_at, | -0.03 | 3.98 | 4.07 | -1.62 | 1.08E-01 | 0.52 |
| ENSG00000282057.1 | RP4-621F18.2 | 237290_at,240259_at,243450_at, | -0.04 | 4.06 | 4.17 | -1.61 | 1.10E-01 | 0.52 |
| ENSG00000237290.1 | LINC01343 | 235591_at, | 0.04 | 2.63 | 2.56 | 1.62 | 1.10E-01 | 0.52 |
| ENSG00000280639.1 | RP11-96C21.2 | 1561673_at, | -0.04 | 3.17 | 3.25 | -1.62 | 1.09E-01 | 0.52 |
| ENSG00000225602.5 | MTOR-AS1 | 235572_at, | 0.05 | 5.93 | 5.74 | 1.62 | 1.09E-01 | 0.52 |
| ENSG00000242516.1 | LINC00960 | 1559827_at,1564175_at, | 0.07 | 3.41 | 3.26 | 1.62 | 1.09E-01 | 0.52 |
| ENSG00000248138.5 | RP11-446J8.1 | 206481_s_at, | -0.07 | 4.95 | 5.22 | -1.61 | 1.10E-01 | 0.52 |
| ENSG00000260761.1 | RP11-184E9.2 | 1562866_at, | -0.03 | 2.59 | 2.64 | -1.61 | 1.10E-01 | 0.52 |
| ENSG00000230782.1 | RP11-508N12.2 | 226575_at, | 0.07 | 7.07 | 6.73 | 1.61 | 1.10E-01 | 0.52 |
| ENSG00000230185.4 | C9orf147 | 209512_at, | -0.09 | 4.30 | 4.58 | -1.62 | 1.08E-01 | 0.52 |
| ENSG00000227303.1 | GS1-256O22.5 | 234368_at, | -0.03 | 2.37 | 2.42 | -1.62 | 1.09E-01 | 0.52 |
| ENSG00000236513.1 | RP11-707P20.1 | 1562973_at, | 0.03 | 2.75 | 2.70 | 1.61 | 1.10E-01 | 0.52 |
| ENSG00000234690.6 | AC073283.4 | 237223_at,1562604_at,237501_at,207130_at,224034_at,  209049_s_at,1558477_at, | -0.02 | 3.79 | 3.86 | -1.61 | 1.11E-01 | 0.52 |
| ENSG00000228035.1 | RP4-663N10.1 | 206814_at,1564819_at, | 0.04 | 4.76 | 4.64 | 1.60 | 1.14E-01 | 0.53 |
| ENSG00000256034.1 | RP11-707G14.1 | 201045_s_at, | 0.03 | 4.27 | 4.17 | 1.60 | 1.14E-01 | 0.53 |
| ENSG00000245534.6 | RORA-AS1 | 1562529_s_at,236266_at,1562682_at,1562528_at, | -0.04 | 4.57 | 4.69 | -1.60 | 1.14E-01 | 0.53 |
| ENSG00000260816.2 | RP11-319G9.3 | 237035_at, | -0.10 | 3.67 | 3.94 | -1.60 | 1.13E-01 | 0.53 |
| ENSG00000263765.5 | RP11-746B8.1 | 1558820_a_at, | -0.05 | 4.15 | 4.28 | -1.59 | 1.14E-01 | 0.53 |
| ENSG00000234945.7 | GTF3C2-AS1 | 1556670_at, | 0.03 | 3.12 | 3.05 | 1.60 | 1.13E-01 | 0.53 |
| ENSG00000242759.6 | LINC00882 | 1569885_at, | 0.03 | 3.14 | 3.08 | 1.59 | 1.14E-01 | 0.53 |
| ENSG00000240207.6 | RP11-379F4.4 | 1565608_at,1565610_at, | -0.03 | 3.09 | 3.15 | -1.60 | 1.14E-01 | 0.53 |
| ENSG00000272906.1 | RP11-533E19.7 | 223929_s_at, | 0.03 | 4.46 | 4.36 | 1.59 | 1.15E-01 | 0.53 |
| ENSG00000261781.1 | RP11-174G17.2 | 1561311_at, | 0.03 | 3.02 | 2.97 | 1.58 | 1.16E-01 | 0.53 |
| ENSG00000246640.1 | RP11-1094H24.4 | 1557608_a_at,1557607_at, | -0.03 | 3.50 | 3.57 | -1.58 | 1.17E-01 | 0.53 |
| ENSG00000132204.13 | LINC00470 | 223977_s_at,224181_at,242276_at, | 0.07 | 2.92 | 2.79 | 1.59 | 1.17E-01 | 0.53 |
| ENSG00000253661.1 | ZFHX4-AS1 | 1559965_at, | -0.03 | 2.47 | 2.52 | -1.58 | 1.17E-01 | 0.53 |
| ENSG00000271746.1 | RP1-202O8.3 | 231413_at, | 0.03 | 3.53 | 3.45 | 1.58 | 1.19E-01 | 0.54 |
| ENSG00000213057.5 | C1orf220 | 1563876_at, | -0.04 | 3.22 | 3.32 | -1.56 | 1.23E-01 | 0.54 |
| ENSG00000229191.1 | RP11-168O16.1 | 233016_at, | 0.03 | 3.97 | 3.88 | 1.55 | 1.24E-01 | 0.54 |
| ENSG00000228971.2 | RP11-286B14.1 | 212123_at,244347_at,226753_at,234202_at,227993_at,239119_at,  204557_s_at,209995_s_at,219840_s_at,239964_at,1566690_at,  1559665_at, | -0.03 | 4.89 | 4.98 | -1.57 | 1.21E-01 | 0.54 |
| ENSG00000224919.1 | RP11-144G6.4 | 240230_s_at, | -0.06 | 4.43 | 4.61 | -1.56 | 1.22E-01 | 0.54 |
| ENSG00000233930.3 | KRTAP5-AS1 | 1566218_at,1562681_at, | 0.03 | 4.20 | 4.13 | 1.57 | 1.20E-01 | 0.54 |
| ENSG00000247595.2 | NA | 235440_at,229594_at,1558262_at, | -0.03 | 4.60 | 4.70 | -1.56 | 1.22E-01 | 0.54 |
| ENSG00000257947.1 | RP11-46I1.1 | 241211_at, | 0.03 | 3.57 | 3.49 | 1.56 | 1.21E-01 | 0.54 |
| ENSG00000250366.2 | TUNAR | 232111_at, | 0.04 | 3.55 | 3.45 | 1.56 | 1.22E-01 | 0.54 |
| ENSG00000247240.7 | UBL7-AS1 | 239792_at,238511_at, | -0.03 | 4.32 | 4.42 | -1.56 | 1.22E-01 | 0.54 |
| ENSG00000260017.1 | RP11-1035H13.2 | 210057_at, | 0.07 | 3.31 | 3.16 | 1.55 | 1.25E-01 | 0.54 |
| ENSG00000233290.1 | RP11-147G16.1 | 208774_at,1569263_at,202856_s_at,217685_at, | 0.03 | 6.36 | 6.22 | 1.55 | 1.24E-01 | 0.54 |
| ENSG00000235373.1 | NA | 218898_at, | 0.05 | 6.03 | 5.81 | 1.55 | 1.24E-01 | 0.54 |
| ENSG00000232931.5 | LINC00342 | 1555898_at, | 0.06 | 3.33 | 3.20 | 1.57 | 1.21E-01 | 0.54 |
| ENSG00000240401.8 | AC012358.8 | 239233_at,237291_at,225045_at, | -0.06 | 3.55 | 3.68 | -1.56 | 1.22E-01 | 0.54 |
| ENSG00000230613.1 | HM13-AS1 | 230107_at, | 0.03 | 4.80 | 4.70 | 1.57 | 1.20E-01 | 0.54 |
| ENSG00000261035.1 | RP11-151E14.1 | 1561354_at, | 0.03 | 3.77 | 3.69 | 1.58 | 1.18E-01 | 0.54 |
| ENSG00000185433.8 | LINC00158 | 231303_at, | 0.03 | 2.76 | 2.70 | 1.58 | 1.19E-01 | 0.54 |
| ENSG00000236519.1 | LINC01424 | 242798_at, | 0.04 | 2.76 | 2.68 | 1.55 | 1.23E-01 | 0.54 |
| ENSG00000242428.5 | C3orf67-AS1 | 233707_at, | -0.04 | 3.22 | 3.30 | -1.55 | 1.23E-01 | 0.54 |
| ENSG00000198590.11 | C3orf35 | 237368_at, | 0.03 | 3.36 | 3.30 | 1.57 | 1.20E-01 | 0.54 |
| ENSG00000250910.7 | AC097467.2 | 210730_s_at,239415_at,235550_at, | 0.03 | 4.04 | 3.96 | 1.56 | 1.22E-01 | 0.54 |
| ENSG00000230551.4 | CTB-89H12.4 | 213860_x_at,1556006_s_at,240221_at,1556007_s_at,208865_at, | -0.04 | 7.18 | 7.37 | -1.56 | 1.22E-01 | 0.54 |
| ENSG00000249249.1 | AC010226.4 | 214658_at, | -0.07 | 6.52 | 6.83 | -1.55 | 1.24E-01 | 0.54 |
| ENSG00000248733.1 | CTD-2176I21.2 | 229300_at, | -0.04 | 3.39 | 3.48 | -1.58 | 1.18E-01 | 0.54 |
| ENSG00000272163.1 | GS1-72M22.1 | 223902_at, | 0.04 | 3.37 | 3.29 | 1.57 | 1.20E-01 | 0.54 |
| ENSG00000226031.5 | FGF13-AS1 | 1560537_at, | -0.04 | 3.05 | 3.12 | -1.56 | 1.22E-01 | 0.54 |
| ENSG00000260368.1 | RP11-521I2.3 | 1556496_a_at, | 0.04 | 3.95 | 3.84 | 1.55 | 1.26E-01 | 0.54 |
| ENSG00000258791.7 | LINC00520 | 1555786_s_at, | -0.04 | 7.12 | 7.31 | -1.54 | 1.27E-01 | 0.54 |
| ENSG00000204187.5 | LINC00619 | 1560851_at, | -0.03 | 2.61 | 2.66 | -1.53 | 1.28E-01 | 0.54 |
| ENSG00000265142.6 | MIR133A1HG | 236573_at, | -0.05 | 2.42 | 2.50 | -1.54 | 1.27E-01 | 0.54 |
| ENSG00000225057.2 | AC096574.4 | 205251_at, | -0.05 | 6.85 | 7.08 | -1.53 | 1.28E-01 | 0.54 |
| ENSG00000273106.1 | RP11-559M23.1 | 239532_at, | 0.03 | 5.30 | 5.18 | 1.54 | 1.28E-01 | 0.54 |
| ENSG00000272758.5 | RP11-299J3.8 | 202055_at, | -0.04 | 6.41 | 6.59 | -1.54 | 1.28E-01 | 0.54 |
| ENSG00000224078.12 | SNHG14 | 213291_s_at,1559545_at,211575_s_at,234163_at,213128_s_at,  1559343_at,241834_at,221974_at,221001_at,1559546_s_at,  234386_s_at, | -0.03 | 4.45 | 4.56 | -1.53 | 1.29E-01 | 0.54 |
| ENSG00000279397.1 | RP11-196B3.4 | 234806_at,234855_at, | 0.03 | 3.05 | 2.99 | 1.53 | 1.29E-01 | 0.54 |
| ENSG00000270792.5 | RP11-103J8.1 | 209097_s_at,241338_at, | 0.03 | 4.22 | 4.12 | 1.54 | 1.28E-01 | 0.54 |
| ENSG00000247137.8 | RP11-727A23.5 | 220572_at, | 0.05 | 4.49 | 4.33 | 1.52 | 1.31E-01 | 0.54 |
| ENSG00000179523.4 | EIF3J-AS1 | 235124_at, | -0.04 | 5.20 | 5.36 | -1.52 | 1.31E-01 | 0.54 |
| ENSG00000262136.1 | CTD-2033A16.3 | 223018_at, | 0.04 | 7.15 | 6.93 | 1.53 | 1.30E-01 | 0.54 |
| ENSG00000269564.1 | AC008753.4 | 237882_at, | 0.03 | 5.14 | 5.04 | 1.52 | 1.31E-01 | 0.54 |
| ENSG00000249717.1 | RP11-44F21.3 | 221183_at, | -0.03 | 3.73 | 3.81 | -1.52 | 1.31E-01 | 0.54 |
| ENSG00000248572.5 | EGFLAM-AS2 | 1556740_at, | -0.02 | 3.79 | 3.86 | -1.52 | 1.31E-01 | 0.54 |
| ENSG00000221817.9 | PPP3CB-AS1 | 235772_at, | -0.03 | 4.39 | 4.47 | -1.52 | 1.32E-01 | 0.55 |
| ENSG00000257337.6 | RP11-983P16.4 | 219599_at, | -0.04 | 6.71 | 6.92 | -1.52 | 1.32E-01 | 0.55 |
| ENSG00000269973.1 | RP11-95D17.1 | 235673_at, | 0.02 | 5.81 | 5.71 | 1.52 | 1.32E-01 | 0.55 |
| ENSG00000237505.6 | PKN2-AS1 | 1569873_at,238634_x_at,1569874_s_at,1556706_at,213063_at,  1568777_at,226108_at,224079_at, | -0.01 | 3.79 | 3.82 | -1.51 | 1.34E-01 | 0.55 |
| ENSG00000237416.6 | NA | 216058_s_at, | -0.03 | 3.16 | 3.22 | -1.51 | 1.35E-01 | 0.55 |
| ENSG00000277173.1 | CTD-2024F21.1 | 239103_at, | -0.03 | 2.97 | 3.04 | -1.50 | 1.36E-01 | 0.55 |
| ENSG00000260830.1 | RP11-524O1.4 | 1559927_a_at, | 0.02 | 4.48 | 4.42 | 1.50 | 1.36E-01 | 0.55 |
| ENSG00000258768.2 | CTD-2292M16.8 | 1558606_s_at,1558605_at, | -0.05 | 4.49 | 4.64 | -1.50 | 1.37E-01 | 0.55 |
| ENSG00000265666.1 | RARA-AS1 | 228037_at, | 0.04 | 5.89 | 5.72 | 1.50 | 1.37E-01 | 0.55 |
| ENSG00000215424.9 | MCM3AP-AS1 | 232740_at, | 0.04 | 3.70 | 3.59 | 1.51 | 1.35E-01 | 0.55 |
| ENSG00000250608.1 | RP11-933H2.4 | 232251_at,1568593_a_at,219542_at, | -0.03 | 4.19 | 4.29 | -1.50 | 1.37E-01 | 0.55 |
| ENSG00000279726.1 | AC005609.16 | 211870_s_at,210572_at,211365_s_at, | 0.03 | 3.41 | 3.34 | 1.50 | 1.37E-01 | 0.55 |
| ENSG00000251538.5 | RP11-166A12.1 | 1562801_at, | 0.03 | 4.01 | 3.93 | 1.51 | 1.35E-01 | 0.55 |
| ENSG00000236256.9 | DIAPH2-AS1 | 237778_at, | 0.05 | 3.41 | 3.30 | 1.50 | 1.36E-01 | 0.55 |
| ENSG00000260060.1 | RP11-388M20.1 | 1564802_at, | 0.03 | 3.49 | 3.41 | 1.50 | 1.37E-01 | 0.55 |
| ENSG00000226386.1 | PARD3-AS1 | 230545_at, | 0.05 | 3.12 | 3.00 | 1.50 | 1.38E-01 | 0.55 |
| ENSG00000254815.5 | RP11-496I9.1 | 1569452_at, | 0.04 | 4.21 | 4.09 | 1.49 | 1.39E-01 | 0.56 |
| ENSG00000226605.1 | AC007098.1 | 237427_at,235694_at, | 0.03 | 4.08 | 3.99 | 1.49 | 1.39E-01 | 0.56 |
| ENSG00000258457.5 | RP11-298I3.4 | 219009_at, | -0.03 | 4.40 | 4.50 | -1.49 | 1.41E-01 | 0.56 |
| ENSG00000225315.2 | RP11-293P20.2 | 1555194_at,1559449_a_at, | -0.04 | 2.97 | 3.06 | -1.49 | 1.40E-01 | 0.56 |
| ENSG00000227757.3 | AP000282.2 | 1559771_at, | 0.04 | 3.09 | 3.02 | 1.49 | 1.40E-01 | 0.56 |
| ENSG00000271270.5 | TMCC1-AS1 | 1558480_at, | 0.03 | 2.40 | 2.36 | 1.48 | 1.41E-01 | 0.56 |
| ENSG00000197291.8 | RAMP2-AS1 | 232731_x_at,1564670_at, | -0.04 | 3.83 | 3.93 | -1.48 | 1.42E-01 | 0.56 |
| ENSG00000270820.5 | RP11-355B11.2 | 208775_at,230729_at, | -0.03 | 6.99 | 7.14 | -1.48 | 1.42E-01 | 0.56 |
| ENSG00000227161.1 | AC092755.4 | 209308_s_at, | -0.03 | 5.98 | 6.12 | -1.48 | 1.43E-01 | 0.56 |
| ENSG00000260366.1 | CTD-2639E6.4 | 1556823_s_at, | -0.04 | 3.19 | 3.27 | -1.48 | 1.43E-01 | 0.56 |
| ENSG00000279786.1 | CTD-2014D20.1 | 234211_at,234215_at, | -0.03 | 3.26 | 3.34 | -1.47 | 1.44E-01 | 0.56 |
| ENSG00000231090.1 | RP11-101C11.1 | 212386_at, | -0.04 | 9.08 | 9.35 | -1.47 | 1.44E-01 | 0.57 |
| ENSG00000116652.6 | DLEU2L | 215629_s_at, | -0.07 | 3.81 | 4.01 | -1.47 | 1.46E-01 | 0.57 |
| ENSG00000254963.1 | CTD-2562J17.9 | 228444_at, | 0.03 | 5.76 | 5.63 | 1.47 | 1.46E-01 | 0.57 |
| ENSG00000256195.2 | RP11-64D24.4 | 1569753_at, | -0.05 | 2.79 | 2.89 | -1.46 | 1.47E-01 | 0.57 |
| ENSG00000249873.6 | RP11-983C2.2 | 240268_at, | -0.04 | 2.75 | 2.83 | -1.46 | 1.47E-01 | 0.57 |
| ENSG00000228592.1 | D21S2088E | 1559470_at, | -0.02 | 2.71 | 2.75 | -1.46 | 1.47E-01 | 0.57 |
| ENSG00000228487.2 | RP13-225O21.2 | 242689_at, | -0.02 | 3.74 | 3.80 | -1.46 | 1.47E-01 | 0.57 |
| ENSG00000231252.1 | NA | 234235_at,237527_at,224355_s_at,1564194_a_at,1556828_at,  204566_at,230330_at, | -0.02 | 3.76 | 3.81 | -1.45 | 1.50E-01 | 0.57 |
| ENSG00000269967.1 | RP11-84A19.4 | 208615_s_at,220176_at,219540_at,241025_at, | -0.02 | 5.16 | 5.24 | -1.45 | 1.50E-01 | 0.57 |
| ENSG00000241073.1 | RP4-714D9.2 | 242374_at, | -0.06 | 3.90 | 4.08 | -1.44 | 1.52E-01 | 0.57 |
| ENSG00000165511.6 | C10orf25 | 1552422_at, | -0.04 | 4.43 | 4.56 | -1.45 | 1.49E-01 | 0.57 |
| ENSG00000184224.3 | C11orf72 | 1553438_at, | -0.02 | 5.06 | 5.13 | -1.44 | 1.52E-01 | 0.57 |
| ENSG00000258804.1 | RP11-322L20.1 | 1561442_at, | -0.05 | 2.69 | 2.79 | -1.45 | 1.49E-01 | 0.57 |
| ENSG00000259327.1 | CTD-2184D3.6 | 204000_at, | -0.04 | 6.71 | 6.89 | -1.45 | 1.51E-01 | 0.57 |
| ENSG00000260661.1 | RP11-152L20.3 | 210542_s_at,1562927_at,229776_at, | 0.03 | 2.81 | 2.76 | 1.46 | 1.49E-01 | 0.57 |
| ENSG00000260853.1 | RP11-264B17.2 | 217527_s_at, | -0.03 | 7.77 | 7.92 | -1.46 | 1.49E-01 | 0.57 |
| ENSG00000275210.1 | AC008984.2 | 1555634_a_at, | 0.02 | 3.62 | 3.57 | 1.45 | 1.51E-01 | 0.57 |
| ENSG00000233922.2 | AL133493.2 | 1568817_at, | -0.06 | 2.73 | 2.85 | -1.45 | 1.52E-01 | 0.57 |
| ENSG00000280219.1 | RP11-752L20.3 | 244832_at,243555_at, | -0.05 | 3.34 | 3.46 | -1.44 | 1.52E-01 | 0.57 |
| ENSG00000249673.6 | NOP14-AS1 | 230476_at, | 0.03 | 4.12 | 4.05 | 1.45 | 1.51E-01 | 0.57 |
| ENSG00000246526.2 | RP11-539L10.2 | 1562698_x_at,1562697_at, | -0.03 | 5.92 | 6.04 | -1.46 | 1.48E-01 | 0.57 |
| ENSG00000249700.8 | SRD5A3-AS1 | 1568615_a_at, | 0.06 | 3.00 | 2.88 | 1.45 | 1.52E-01 | 0.57 |
| ENSG00000281016.1 | RP11-2H3.7 | 220159_at, | -0.04 | 4.65 | 4.78 | -1.45 | 1.51E-01 | 0.57 |
| ENSG00000249001.5 | RP11-742B18.1 | 217067_s_at, | 0.03 | 2.59 | 2.53 | 1.46 | 1.49E-01 | 0.57 |
| ENSG00000245532.5 | NEAT1 | 224566_at,214657_s_at, | -0.07 | 7.19 | 7.56 | -1.44 | 1.53E-01 | 0.57 |
| ENSG00000235426.2 | RP11-342M3.5 | 212423_at,1559642_a_at, | -0.03 | 5.25 | 5.37 | -1.43 | 1.55E-01 | 0.57 |
| ENSG00000254721.1 | RP11-805J14.5 | 202535_at, | 0.05 | 6.62 | 6.40 | 1.44 | 1.54E-01 | 0.57 |
| ENSG00000267872.1 | RP11-157B13.7 | 1566689_at, | -0.02 | 3.34 | 3.40 | -1.44 | 1.54E-01 | 0.57 |
| ENSG00000279083.1 | RP11-395N21.2 | 222973_at, | 0.03 | 3.75 | 3.68 | 1.44 | 1.55E-01 | 0.57 |
| ENSG00000232748.3 | RP11-196G11.6 | 219047_s_at, | 0.03 | 5.47 | 5.34 | 1.43 | 1.56E-01 | 0.58 |
| ENSG00000241860.6 | RP11-34P13.13 | 236045_x_at, | -0.07 | 5.11 | 5.37 | -1.42 | 1.57E-01 | 0.58 |
| ENSG00000267193.5 | RP11-116O18.3 | 208409_at, | -0.02 | 4.85 | 4.93 | -1.42 | 1.58E-01 | 0.58 |
| ENSG00000268823.2 | CTC-457E21.6 | 237977_at, | 0.02 | 5.59 | 5.51 | 1.42 | 1.58E-01 | 0.58 |
| ENSG00000230027.1 | RP11-550H2.2 | 208753_s_at,208754_s_at,1568699_at, | -0.03 | 6.41 | 6.55 | -1.42 | 1.59E-01 | 0.58 |
| ENSG00000237298.8 | TTN-AS1 | 237775_x_at,1561230_at,240793_at,244839_at,208195_at, | -0.05 | 3.63 | 3.75 | -1.42 | 1.59E-01 | 0.58 |
| ENSG00000261317.1 | RP11-566K11.5 | 229171_at,223728_at,1552330_at, | 0.03 | 4.50 | 4.40 | 1.42 | 1.60E-01 | 0.58 |
| ENSG00000268818.2 | CITF22-62D4.1 | 224117_at, | 0.03 | 3.98 | 3.89 | 1.42 | 1.60E-01 | 0.58 |
| ENSG00000267219.1 | AC010504.2 | 203288_at, | -0.04 | 6.91 | 7.10 | -1.41 | 1.60E-01 | 0.58 |
| ENSG00000254842.6 | RP11-890B15.2 | 1552972_at, | -0.15 | 2.49 | 2.77 | -1.42 | 1.61E-01 | 0.58 |
| ENSG00000231992.1 | RP11-57H12.2 | 212570_at, | -0.05 | 5.71 | 5.91 | -1.41 | 1.61E-01 | 0.58 |
| ENSG00000245498.6 | RP11-677M14.7 | 1563776_at, | -0.03 | 3.31 | 3.38 | -1.41 | 1.62E-01 | 0.59 |
| ENSG00000250116.2 | RP11-417F21.1 | 1556663_s_at,1556662_at, | 0.02 | 3.91 | 3.84 | 1.41 | 1.62E-01 | 0.59 |
| ENSG00000226835.1 | NA | 218889_at, | -0.04 | 6.68 | 6.86 | -1.41 | 1.63E-01 | 0.59 |
| ENSG00000281106.2 | LINC00282 | 1557465_at, | -0.03 | 3.14 | 3.20 | -1.40 | 1.64E-01 | 0.59 |
| ENSG00000246223.8 | LINC01550 | 240394_at,1564211_at, | 0.02 | 3.43 | 3.38 | 1.41 | 1.63E-01 | 0.59 |
| ENSG00000254101.5 | RP11-30J20.1 | 1561778_at, | 0.07 | 3.12 | 2.97 | 1.41 | 1.64E-01 | 0.59 |
| ENSG00000231535.5 | LINC00278 | 1560800_at,1564510_at, | 0.02 | 2.72 | 2.68 | 1.40 | 1.64E-01 | 0.59 |
| ENSG00000231246.1 | RP5-965F6.2 | 1557529_at, | -0.04 | 3.96 | 4.06 | -1.40 | 1.64E-01 | 0.59 |
| ENSG00000258428.5 | RP11-1085N6.2 | 219757_s_at, | -0.04 | 6.24 | 6.41 | -1.40 | 1.65E-01 | 0.59 |
| ENSG00000237463.5 | RP11-280O1.2 | 1561693_at,1557761_s_at, | -0.03 | 3.52 | 3.58 | -1.39 | 1.67E-01 | 0.59 |
| ENSG00000278811.4 | LINC00624 | 238070_at, | 0.04 | 5.34 | 5.19 | 1.39 | 1.68E-01 | 0.60 |
| ENSG00000250733.5 | C8orf17 | 208266_at, | 0.03 | 3.18 | 3.11 | 1.39 | 1.68E-01 | 0.60 |
| ENSG00000273071.1 | RP11-337C18.10 | 1569688_at,215300_s_at,205776_at, | -0.05 | 3.05 | 3.15 | -1.39 | 1.68E-01 | 0.60 |
| ENSG00000280046.1 | RP11-1099M24.6 | 226833_at, | -0.06 | 5.54 | 5.77 | -1.39 | 1.69E-01 | 0.60 |
| ENSG00000249592.5 | RP11-440L14.1 | 229413_s_at,230408_at, | -0.05 | 5.76 | 5.95 | -1.39 | 1.69E-01 | 0.60 |
| ENSG00000271763.1 | RP11-386M24.9 | 221880_s_at, | -0.04 | 7.79 | 8.02 | -1.38 | 1.70E-01 | 0.60 |
| ENSG00000266469.1 | CTB-131K11.1 | 225456_at, | 0.10 | 5.56 | 5.19 | 1.38 | 1.70E-01 | 0.60 |
| ENSG00000269926.1 | RP11-442H21.2 | 202887_s_at, | 0.05 | 8.76 | 8.48 | 1.38 | 1.72E-01 | 0.60 |
| ENSG00000227712.1 | RP11-418J17.3 | 225231_at,1564060_at, | -0.04 | 6.49 | 6.67 | -1.38 | 1.71E-01 | 0.60 |
| ENSG00000251239.1 | CTB-22K21.2 | 207869_s_at, | -0.03 | 2.42 | 2.46 | -1.38 | 1.72E-01 | 0.60 |
| ENSG00000267437.1 | CTC-454I21.4 | 238937_at,242761_s_at, | -0.03 | 6.41 | 6.56 | -1.37 | 1.73E-01 | 0.60 |
| ENSG00000231898.8 | AC012594.1 | 1560788_at, | -0.07 | 2.77 | 2.89 | -1.38 | 1.72E-01 | 0.60 |
| ENSG00000225421.1 | AC019330.1 | 234581_at,234606_at, | -0.02 | 2.65 | 2.69 | -1.37 | 1.72E-01 | 0.60 |
| ENSG00000239454.1 | RP11-508O18.1 | 1561519_at, | -0.03 | 2.71 | 2.78 | -1.37 | 1.73E-01 | 0.60 |
| ENSG00000224186.8 | C5orf66 | 229593_at,236821_at, | -0.02 | 4.54 | 4.61 | -1.37 | 1.73E-01 | 0.60 |
| ENSG00000280064.1 | RP11-205M5.3 | 225177_at, | -0.06 | 6.83 | 7.13 | -1.38 | 1.72E-01 | 0.60 |
| ENSG00000244558.5 | KCNK15-AS1 | 205792_at, | -0.07 | 4.97 | 5.23 | -1.37 | 1.74E-01 | 0.60 |
| ENSG00000272405.1 | RP11-284F21.10 | 223633_s_at,223632_s_at, | 0.03 | 3.66 | 3.58 | 1.37 | 1.75E-01 | 0.60 |
| ENSG00000266729.5 | DSG1-AS1 | 1561330_at, | -0.03 | 2.43 | 2.48 | -1.37 | 1.75E-01 | 0.60 |
| ENSG00000175611.11 | LINC00476 | 234835_at,1557788_a_at, | -0.02 | 3.61 | 3.67 | -1.36 | 1.75E-01 | 0.60 |
| ENSG00000257913.2 | RP11-386G11.5 | 201805_at, | -0.03 | 6.83 | 6.96 | -1.36 | 1.76E-01 | 0.60 |
| ENSG00000240291.1 | RP11-499P20.2 | 213714_at,207776_s_at, | -0.05 | 3.27 | 3.38 | -1.36 | 1.77E-01 | 0.61 |
| ENSG00000279877.1 | RP11-420N3.3 | 1570215_at, | -0.03 | 3.18 | 3.25 | -1.35 | 1.79E-01 | 0.61 |
| ENSG00000267169.1 | CTB-55O6.12 | 219145_at,47560_at,203488_at, | 0.03 | 6.05 | 5.92 | 1.35 | 1.79E-01 | 0.61 |
| ENSG00000261824.6 | LINC00662 | 242663_at,1558256_at, | -0.03 | 3.44 | 3.52 | -1.35 | 1.79E-01 | 0.61 |
| ENSG00000260912.1 | RP11-363E7.4 | 227306_at, | -0.07 | 4.05 | 4.26 | -1.36 | 1.78E-01 | 0.61 |
| ENSG00000232828.1 | AF196970.3 | 218619_s_at, | 0.03 | 6.26 | 6.12 | 1.36 | 1.78E-01 | 0.61 |
| ENSG00000269289.5 | CTB-92J24.3 | 1568648_a_at,1568647_at, | -0.05 | 2.98 | 3.08 | -1.35 | 1.80E-01 | 0.61 |
| ENSG00000227963.1 | RP5-1074L1.1 | 200071_at, | -0.03 | 8.28 | 8.45 | -1.35 | 1.82E-01 | 0.61 |
| ENSG00000228065.10 | LINC01515 | 233908_x_at, | 0.06 | 5.37 | 5.17 | 1.33 | 1.85E-01 | 0.61 |
| ENSG00000279689.1 | RP11-574K11.32 | 202361_at, | -0.03 | 7.77 | 7.93 | -1.34 | 1.84E-01 | 0.61 |
| ENSG00000275963.1 | RP11-180M15.6 | 240774_at, | 0.02 | 4.22 | 4.16 | 1.33 | 1.86E-01 | 0.61 |
| ENSG00000235446.1 | RP11-74K19.1 | 236582_at, | 0.03 | 2.67 | 2.62 | 1.34 | 1.84E-01 | 0.61 |
| ENSG00000203605.3 | RP11-335E6.2 | 238835_at,206250_x_at,238935_at, | -0.02 | 3.77 | 3.83 | -1.33 | 1.86E-01 | 0.61 |
| ENSG00000170919.15 | TPT1-AS1 | 234056_at,234052_at, | 0.02 | 3.83 | 3.77 | 1.33 | 1.86E-01 | 0.61 |
| ENSG00000247556.6 | OIP5-AS1 | 225225_at,225332_at, | -0.04 | 7.35 | 7.53 | -1.34 | 1.83E-01 | 0.61 |
| ENSG00000241818.1 | RP11-1000B6.2 | 1561434_at, | -0.03 | 3.22 | 3.29 | -1.33 | 1.85E-01 | 0.61 |
| ENSG00000259678.1 | RP11-707P17.1 | 212820_at, | -0.05 | 5.70 | 5.91 | -1.34 | 1.83E-01 | 0.61 |
| ENSG00000260874.5 | RP11-715J22.4 | 204826_at,204827_s_at, | 0.02 | 5.64 | 5.55 | 1.34 | 1.85E-01 | 0.61 |
| ENSG00000261738.6 | MIR3976HG | 233610_at, | 0.02 | 3.61 | 3.55 | 1.34 | 1.83E-01 | 0.61 |
| ENSG00000269292.1 | CTB-12A17.3 | 200623_s_at, | -0.03 | 6.04 | 6.15 | -1.35 | 1.81E-01 | 0.61 |
| ENSG00000238018.2 | AC093110.3 | 226765_at, | 0.08 | 4.94 | 4.68 | 1.33 | 1.86E-01 | 0.61 |
| ENSG00000250392.2 | RP11-700N1.1 | 231347_at, | -0.03 | 3.66 | 3.73 | -1.34 | 1.83E-01 | 0.61 |
| ENSG00000231025.1 | RP11-175O19.4 | 226892_at, | -0.03 | 5.27 | 5.38 | -1.33 | 1.86E-01 | 0.61 |
| ENSG00000247131.5 | RP11-588G21.2 | 1557606_at, | 0.03 | 2.93 | 2.87 | 1.33 | 1.87E-01 | 0.62 |
| ENSG00000250132.6 | RP11-359B12.2 | 235820_at, | 0.03 | 3.17 | 3.11 | 1.33 | 1.88E-01 | 0.62 |
| ENSG00000251536.2 | RP11-572C21.1 | 1559629_at, | 0.02 | 4.78 | 4.70 | 1.33 | 1.88E-01 | 0.62 |
| ENSG00000234377.7 | RNF219-AS1 | 239997_at,1561298_at,231354_at, | -0.03 | 3.40 | 3.48 | -1.32 | 1.88E-01 | 0.62 |
| ENSG00000267379.1 | CTC-548K16.5 | 202910_s_at, | 0.05 | 6.16 | 5.94 | 1.33 | 1.87E-01 | 0.62 |
| ENSG00000268729.1 | CTD-2105E13.14 | 1552504_a_at, | 0.03 | 5.46 | 5.36 | 1.32 | 1.90E-01 | 0.62 |
| ENSG00000235890.2 | TSPEAR-AS1 | 1555048_a_at,1555049_at, | 0.03 | 3.41 | 3.34 | 1.32 | 1.90E-01 | 0.62 |
| ENSG00000227398.3 | KIF9-AS1 | 1557613_at, | 0.02 | 4.87 | 4.81 | 1.32 | 1.90E-01 | 0.62 |
| ENSG00000235455.1 | IQCF5-AS1 | 231525_at, | 0.03 | 3.04 | 2.97 | 1.32 | 1.90E-01 | 0.62 |
| ENSG00000253961.1 | RP11-363L24.3 | 205667_at, | -0.03 | 5.62 | 5.74 | -1.32 | 1.91E-01 | 0.62 |
| ENSG00000226944.1 | RP1-120G22.11 | 237940_s_at,237941_at, | 0.02 | 3.75 | 3.70 | 1.32 | 1.91E-01 | 0.62 |
| ENSG00000261646.1 | RP11-489G11.3 | 1570046_at, | 0.02 | 2.47 | 2.43 | 1.31 | 1.92E-01 | 0.62 |
| ENSG00000240553.1 | RP1-184J9.2 | 221832_s_at,225831_at,238652_at,243298_at,201715_s_at,  1558117_s_at,226033_at,244441_at, | 0.01 | 5.43 | 5.38 | 1.31 | 1.93E-01 | 0.62 |
| ENSG00000260526.1 | RP11-73K9.2 | 1559103_s_at,1559102_at, | -0.02 | 2.86 | 2.90 | -1.31 | 1.93E-01 | 0.62 |
| ENSG00000230590.7 | FTX | 1569311_at,1558515_at,242121_at,229315_at, | -0.03 | 7.24 | 7.40 | -1.31 | 1.93E-01 | 0.62 |
| ENSG00000271955.1 | RP11-444A22.1 | 1566600_at,225313_at,227949_at,208399_s_at,204554_at,236938_at,  1553311_at,1558826_at,1558827_a_at, | -0.02 | 3.78 | 3.83 | -1.31 | 1.94E-01 | 0.62 |
| ENSG00000235978.6 | AC018816.3 | 216944_s_at, | -0.05 | 4.26 | 4.42 | -1.31 | 1.93E-01 | 0.62 |
| ENSG00000253807.5 | LINC01170 | 1564813_at, | -0.02 | 2.45 | 2.49 | -1.31 | 1.94E-01 | 0.62 |
| ENSG00000241772.2 | AC092620.2 | 243170_at,225659_at,225658_at, | -0.05 | 6.46 | 6.68 | -1.31 | 1.94E-01 | 0.62 |
| ENSG00000223907.1 | LINC01226 | 212758_s_at,237198_at, | -0.03 | 5.59 | 5.71 | -1.30 | 1.95E-01 | 0.62 |
| ENSG00000251448.1 | RP11-71E19.2 | 222340_at, | -0.04 | 3.38 | 3.47 | -1.30 | 1.95E-01 | 0.62 |
| ENSG00000235079.1 | ZRANB2-AS1 | 223716_s_at, | -0.04 | 8.46 | 8.67 | -1.31 | 1.96E-01 | 0.62 |
| ENSG00000177133.10 | LINC00982 | 230675_at,207890_s_at, | 0.02 | 5.22 | 5.13 | 1.30 | 1.96E-01 | 0.62 |
| ENSG00000260683.1 | CTD-2076M15.1 | 1561713_at, | -0.05 | 2.74 | 2.84 | -1.30 | 1.96E-01 | 0.62 |
| ENSG00000228863.8 | RP11-404F10.2 | 237759_at, | -0.04 | 4.42 | 4.54 | -1.30 | 1.99E-01 | 0.63 |
| ENSG00000258461.5 | RP11-164J13.1 | 236077_at, | -0.03 | 4.84 | 4.95 | -1.29 | 1.98E-01 | 0.63 |
| ENSG00000259953.1 | RP11-4O1.2 | 236769_at, | -0.06 | 3.90 | 4.06 | -1.30 | 1.98E-01 | 0.63 |
| ENSG00000235823.1 | OLMALINC | 232230_at, | -0.05 | 3.24 | 3.36 | -1.29 | 2.01E-01 | 0.63 |
| ENSG00000258658.1 | RP11-517O13.3 | 230141_at, | -0.06 | 4.06 | 4.23 | -1.28 | 2.02E-01 | 0.63 |
| ENSG00000260046.1 | RP11-454K7.3 | 1559813_at, | -0.03 | 3.11 | 3.17 | -1.29 | 2.01E-01 | 0.63 |
| ENSG00000260125.1 | AGBL1-AS1 | 1553447_at, | -0.03 | 3.17 | 3.24 | -1.28 | 2.02E-01 | 0.63 |
| ENSG00000280254.1 | RP11-81A22.4 | 1558723_at, | -0.02 | 4.06 | 4.12 | -1.29 | 2.00E-01 | 0.63 |
| ENSG00000226925.1 | AC007271.3 | 215561_s_at, | 0.04 | 3.51 | 3.42 | 1.29 | 2.00E-01 | 0.63 |
| ENSG00000235351.1 | AC114730.11 | 229197_at, | 0.02 | 3.29 | 3.24 | 1.29 | 2.02E-01 | 0.63 |
| ENSG00000236204.5 | LINC01376 | 210010_s_at,204383_at,215003_at,216285_at,216145_at,  1569560_at,217285_at,217275_at,1566235_at, | 0.01 | 4.91 | 4.86 | 1.28 | 2.02E-01 | 0.63 |
| ENSG00000231711.2 | LINC00899 | 1556304_s_at, | -0.04 | 4.59 | 4.72 | -1.29 | 2.02E-01 | 0.63 |
| ENSG00000245213.6 | RP11-10K16.1 | 240212_at, | 0.03 | 2.88 | 2.83 | 1.28 | 2.03E-01 | 0.63 |
| ENSG00000279799.1 | AC006077.3 | 231085_s_at,230984_s_at, | 0.02 | 3.44 | 3.38 | 1.29 | 2.00E-01 | 0.63 |
| ENSG00000237975.6 | FLG-AS1 | 1570423_at,1569410_at,220090_at, | -0.02 | 3.26 | 3.30 | -1.28 | 2.03E-01 | 0.63 |
| ENSG00000203721.5 | LINC00862 | 1562909_at, | 0.03 | 2.77 | 2.71 | 1.28 | 2.05E-01 | 0.63 |
| ENSG00000255443.1 | RP1-68D18.4 | 234411_x_at,234418_x_at, | 0.03 | 3.35 | 3.29 | 1.27 | 2.06E-01 | 0.63 |
| ENSG00000277688.1 | RP11-697E22.1 | 213637_at, | 0.06 | 5.49 | 5.26 | 1.27 | 2.06E-01 | 0.63 |
| ENSG00000264514.1 | RP11-720L2.4 | 240397_x_at, | -0.04 | 3.88 | 3.98 | -1.27 | 2.06E-01 | 0.63 |
| ENSG00000267651.1 | RP11-95O2.1 | 233602_at, | 0.03 | 2.64 | 2.60 | 1.28 | 2.05E-01 | 0.63 |
| ENSG00000267268.1 | AC007204.2 | 215758_x_at, | -0.02 | 4.70 | 4.78 | -1.28 | 2.05E-01 | 0.63 |
| ENSG00000241158.5 | ADAMTS9-AS1 | 1556413_a_at,1562275_at, | 0.03 | 3.22 | 3.14 | 1.27 | 2.07E-01 | 0.63 |
| ENSG00000228420.1 | RP4-735C1.6 | 209045_at,226177_at, | -0.02 | 8.62 | 8.72 | -1.26 | 2.09E-01 | 0.63 |
| ENSG00000280367.1 | RP11-121L10.2 | 1557845_at, | -0.03 | 2.72 | 2.78 | -1.26 | 2.10E-01 | 0.63 |
| ENSG00000256469.1 | RP11-856F16.2 | 225459_at,225450_at, | 0.03 | 5.60 | 5.48 | 1.27 | 2.08E-01 | 0.63 |
| ENSG00000271327.1 | RP11-1109F11.3 | 239625_at, | -0.03 | 3.61 | 3.68 | -1.26 | 2.10E-01 | 0.63 |
| ENSG00000278390.4 | RP11-74J13.8 | 229298_at, | -0.05 | 4.66 | 4.81 | -1.26 | 2.11E-01 | 0.63 |
| ENSG00000262119.1 | RP11-483C6.1 | 1570136_at, | -0.02 | 2.58 | 2.61 | -1.26 | 2.10E-01 | 0.63 |
| ENSG00000262160.1 | RP11-96D1.11 | 207416_s_at,225141_at,225137_at, | 0.02 | 6.23 | 6.14 | 1.27 | 2.08E-01 | 0.63 |
| ENSG00000274386.4 | RP5-994D16.11 | 236998_at,229158_at, | -0.06 | 4.47 | 4.67 | -1.26 | 2.10E-01 | 0.63 |
| ENSG00000234953.2 | RP5-837I24.2 | 217796_s_at, | 0.02 | 8.00 | 7.87 | 1.26 | 2.10E-01 | 0.63 |
| ENSG00000223573.6 | TINCR | 229385_s_at, | 0.09 | 4.99 | 4.69 | 1.27 | 2.08E-01 | 0.63 |
| ENSG00000251095.6 | RP11-115D19.1 | 204466_s_at,236081_at, | -0.03 | 3.50 | 3.58 | -1.27 | 2.07E-01 | 0.63 |
| ENSG00000259820.1 | AC083843.1 | 229455_at, | 0.08 | 3.32 | 3.15 | 1.26 | 2.11E-01 | 0.63 |
| ENSG00000231365.5 | RP11-418J17.1 | 1562474_at,231505_s_at,225143_at,207593_at, | -0.02 | 5.71 | 5.77 | -1.26 | 2.12E-01 | 0.63 |
| ENSG00000226752.7 | PSMD5-AS1 | 1555892_s_at,241249_at,215546_at, | 0.02 | 3.58 | 3.53 | 1.26 | 2.11E-01 | 0.63 |
| ENSG00000234323.5 | RP11-308N19.1 | 1570469_at, | -0.06 | 2.58 | 2.69 | -1.26 | 2.12E-01 | 0.63 |
| ENSG00000279031.1 | LA16c-360H6.1 | 239502_at, | -0.02 | 3.24 | 3.29 | -1.25 | 2.13E-01 | 0.63 |
| ENSG00000273784.4 | RP11-78J21.7 | 238696_at, | 0.02 | 2.49 | 2.46 | 1.25 | 2.14E-01 | 0.64 |
| ENSG00000259037.1 | RP11-614O9.1 | 1558420_at, | -0.02 | 5.55 | 5.65 | -1.25 | 2.14E-01 | 0.64 |
| ENSG00000244879.4 | GABPB1-AS1 | 227406_at, | -0.03 | 6.38 | 6.52 | -1.25 | 2.15E-01 | 0.64 |
| ENSG00000260277.1 | RP11-101E7.2 | 230721_at, | -0.03 | 4.88 | 4.98 | -1.25 | 2.15E-01 | 0.64 |
| ENSG00000263412.1 | RP5-890E16.2 | 1559578_at, | 0.03 | 4.02 | 3.95 | 1.25 | 2.15E-01 | 0.64 |
| ENSG00000254349.5 | MIR2052HG | 1561371_at, | -0.02 | 3.75 | 3.81 | -1.25 | 2.16E-01 | 0.64 |
| ENSG00000274961.1 | RP3-492J12.2 | 234798_x_at, | 0.04 | 3.13 | 3.06 | 1.25 | 2.16E-01 | 0.64 |
| ENSG00000226202.2 | RP11-328D5.1 | 239986_at, | -0.02 | 3.43 | 3.49 | -1.24 | 2.17E-01 | 0.64 |
| ENSG00000255723.1 | RP11-529H2.2 | 217989_at, | -0.06 | 6.12 | 6.40 | -1.24 | 2.18E-01 | 0.64 |
| ENSG00000259370.2 | RP11-1069G10.1 | 212701_at,212703_at, | -0.02 | 5.32 | 5.41 | -1.23 | 2.20E-01 | 0.64 |
| ENSG00000230035.2 | RP11-174G17 | 1553504_at,1570071_at,229392_s_at, | 0.03 | 5.36 | 5.27 | 1.24 | 2.20E-01 | 0.64 |
| ENSG00000269707.1 | RP11-13J10.1 | 228780_at,208563_x_at, | 0.03 | 3.37 | 3.30 | 1.23 | 2.20E-01 | 0.64 |
| ENSG00000260322.1 | RP11-339A11.2 | 234824_at,234800_at, | -0.03 | 3.23 | 3.30 | -1.23 | 2.22E-01 | 0.65 |
| ENSG00000267272.5 | LINC01140 | 235870_at, | 0.03 | 4.51 | 4.43 | 1.23 | 2.21E-01 | 0.65 |
| ENSG00000258498.6 | DIO3OS | 239727_at, | 0.02 | 3.75 | 3.69 | 1.23 | 2.22E-01 | 0.65 |
| ENSG00000261744.1 | RP11-21B21.4 | 237463_at, | 0.03 | 5.90 | 5.79 | 1.23 | 2.21E-01 | 0.65 |
| ENSG00000268205.1 | CTC-444N24.11 | 242691_at, | 0.05 | 5.09 | 4.91 | 1.23 | 2.22E-01 | 0.65 |
| ENSG00000273015.2 | RP11-352M15.2 | 228833_s_at, | 0.03 | 2.95 | 2.89 | 1.23 | 2.23E-01 | 0.65 |
| ENSG00000272141.1 | RP11-465B22.8 | 235663_at, | 0.02 | 5.94 | 5.85 | 1.22 | 2.24E-01 | 0.65 |
| ENSG00000256947.1 | RP11-64D24.2 | 231262_at,202237_at, | 0.03 | 9.90 | 9.69 | 1.22 | 2.24E-01 | 0.65 |
| ENSG00000259284.1 | RP11-162I7.1 | 231473_at, | -0.02 | 3.02 | 3.07 | -1.22 | 2.24E-01 | 0.65 |
| ENSG00000242242.5 | PVRL3-AS1 | 1562856_at, | -0.03 | 2.70 | 2.75 | -1.22 | 2.24E-01 | 0.65 |
| ENSG00000223823.1 | LINC01342 | 1562788_at, | -0.03 | 3.79 | 3.86 | -1.22 | 2.26E-01 | 0.65 |
| ENSG00000223883.1 | RP11-424D14.1 | 243316_x_at,241279_at,240957_at,226467_at,224732_at,  1552980_at,237969_at,236184_at, | 0.01 | 3.87 | 3.85 | 1.21 | 2.28E-01 | 0.65 |
| ENSG00000235410.1 | RP11-397C18.2 | 1554034_a_at, | 0.02 | 2.68 | 2.64 | 1.22 | 2.26E-01 | 0.65 |
| ENSG00000225778.5 | PROSER2-AS1 | 1569722_s_at, | 0.03 | 3.36 | 3.29 | 1.22 | 2.26E-01 | 0.65 |
| ENSG00000253352.8 | TUG1 | 212337_at,228397_at,212725_s_at, | 0.04 | 6.73 | 6.56 | 1.21 | 2.28E-01 | 0.65 |
| ENSG00000242086.8 | LINC00969 | 232047_at, | -0.03 | 3.88 | 3.97 | -1.22 | 2.27E-01 | 0.65 |
| ENSG00000259976.1 | RP11-553L6.5 | 213158_at, | -0.05 | 7.58 | 7.84 | -1.22 | 2.27E-01 | 0.65 |
| ENSG00000254226.5 | CTB-12O2.1 | 233237_at,233238_s_at, | 0.02 | 2.96 | 2.91 | 1.22 | 2.26E-01 | 0.65 |
| ENSG00000246316.7 | RP11-492A10.1 | 220116_at, | 0.04 | 3.37 | 3.28 | 1.22 | 2.25E-01 | 0.65 |
| ENSG00000253741.1 | CTD-2292P10.4 | 223688_s_at,223687_s_at, | 0.07 | 4.71 | 4.48 | 1.22 | 2.27E-01 | 0.65 |
| ENSG00000272419.5 | RP11-403I13.8 | 235284_s_at, | 0.02 | 2.83 | 2.79 | 1.21 | 2.28E-01 | 0.65 |
| ENSG00000234694.1 | RP1-92O14.3 | 241454_at, | 0.02 | 3.54 | 3.49 | 1.21 | 2.28E-01 | 0.65 |
| ENSG00000227244.2 | LINC00845 | 238298_at, | -0.03 | 3.20 | 3.26 | -1.21 | 2.30E-01 | 0.65 |
| ENSG00000246263.2 | UBR5-AS1 | 1555888_at,208883_at, | -0.05 | 4.62 | 4.77 | -1.21 | 2.30E-01 | 0.65 |
| ENSG00000267879.1 | CTB-147C22.9 | 1552319_a_at,239381_at, | 0.04 | 4.52 | 4.39 | 1.21 | 2.31E-01 | 0.65 |
| ENSG00000211683.3 | KB-1572G7.3 | 221108_at, | 0.02 | 4.37 | 4.30 | 1.21 | 2.30E-01 | 0.65 |
| ENSG00000224854.3 | CDKN2A-AS1 | 220505_at, | 0.03 | 4.02 | 3.95 | 1.21 | 2.31E-01 | 0.65 |
| ENSG00000237149.5 | ZNF503-AS2 | 235928_at, | 0.05 | 3.31 | 3.20 | 1.20 | 2.33E-01 | 0.65 |
| ENSG00000181995.11 | LINC00301 | 1554765_a_at, | 0.03 | 2.82 | 2.77 | 1.20 | 2.34E-01 | 0.65 |
| ENSG00000257520.1 | RP11-896J10.3 | 231315_at,211024_s_at,228979_at, | -0.04 | 2.77 | 2.85 | -1.20 | 2.34E-01 | 0.65 |
| ENSG00000230324.1 | RP4-705O1.1 | 1562829_at, | -0.02 | 3.04 | 3.09 | -1.20 | 2.34E-01 | 0.65 |
| ENSG00000237978.5 | KCNMB2-AS1 | 238282_at, | -0.03 | 2.72 | 2.77 | -1.20 | 2.33E-01 | 0.65 |
| ENSG00000277801.1 | RP11-681H18.2 | 218031_s_at, | -0.05 | 5.66 | 5.87 | -1.19 | 2.35E-01 | 0.65 |
| ENSG00000260619.1 | RP11-775C24.3 | 216106_at, | -0.03 | 3.67 | 3.75 | -1.20 | 2.35E-01 | 0.65 |
| ENSG00000189229.10 | AC069277.2 | 243670_at, | 0.02 | 4.13 | 4.06 | 1.19 | 2.35E-01 | 0.65 |
| ENSG00000227409.1 | ZMYM4-AS1 | 238744_at, | -0.05 | 3.07 | 3.18 | -1.19 | 2.37E-01 | 0.66 |
| ENSG00000231172.2 | AC007099.1 | 1570230_at, | -0.03 | 3.80 | 3.89 | -1.19 | 2.38E-01 | 0.66 |
| ENSG00000251556.1 | RP11-118M9.3 | 217810_x_at, | -0.02 | 7.39 | 7.48 | -1.19 | 2.38E-01 | 0.66 |
| ENSG00000233078.1 | RP11-5P18.5 | 229381_at, | -0.07 | 5.67 | 5.96 | -1.19 | 2.39E-01 | 0.66 |
| ENSG00000228327.2 | NA | 227968_at, | 0.02 | 7.21 | 7.09 | 1.18 | 2.40E-01 | 0.66 |
| ENSG00000226828.1 | RP11-278H7.1 | 240341_at, | -0.02 | 2.75 | 2.79 | -1.18 | 2.41E-01 | 0.66 |
| ENSG00000249859.7 | PVT1 | 1558290_a_at, | 0.05 | 7.05 | 6.83 | 1.18 | 2.42E-01 | 0.67 |
| ENSG00000263307.1 | RP11-166B2.8 | 240452_at,225276_at, | -0.03 | 7.47 | 7.61 | -1.18 | 2.42E-01 | 0.67 |
| ENSG00000204792.2 | LINC01291 | 1556173_a_at, | -0.03 | 4.89 | 5.00 | -1.18 | 2.42E-01 | 0.67 |
| ENSG00000261319.1 | RP11-279O17.1 | 234434_at, | 0.03 | 3.11 | 3.04 | 1.18 | 2.44E-01 | 0.67 |
| ENSG00000226137.5 | BAIAP2-AS1 | 1566557_at,1566555_at,1558147_a_at, | 0.02 | 5.00 | 4.93 | 1.17 | 2.44E-01 | 0.67 |
| ENSG00000238242.1 | RP11-575B7.3 | 237839_at,219972_s_at,236332_at, | -0.03 | 4.18 | 4.26 | -1.16 | 2.48E-01 | 0.67 |
| ENSG00000231920.1 | NEBL-AS1 | 239894_at, | 0.05 | 4.41 | 4.27 | 1.16 | 2.48E-01 | 0.67 |
| ENSG00000251151.2 | HOXC-AS3 | 1561273_at, | 0.03 | 3.71 | 3.63 | 1.16 | 2.47E-01 | 0.67 |
| ENSG00000275202.1 | RP11-156K23.3 | 231367_s_at, | 0.02 | 3.08 | 3.03 | 1.17 | 2.47E-01 | 0.67 |
| ENSG00000259498.1 | RP11-244F12.3 | 1558532_at, | -0.02 | 3.05 | 3.09 | -1.17 | 2.47E-01 | 0.67 |
| ENSG00000264272.1 | CTD-2514K5.4 | 223741_s_at, | 0.04 | 6.57 | 6.40 | 1.16 | 2.47E-01 | 0.67 |
| ENSG00000228925.1 | AC016722.4 | 212245_at, | 0.03 | 7.35 | 7.19 | 1.17 | 2.45E-01 | 0.67 |
| ENSG00000225610.1 | AC007679.3 | 226555_at, | -0.03 | 5.74 | 5.85 | -1.17 | 2.46E-01 | 0.67 |
| ENSG00000234378.1 | AC098828.2 | 219811_at, | 0.02 | 6.14 | 6.04 | 1.16 | 2.48E-01 | 0.67 |
| ENSG00000245275.7 | SAP30L-AS1 | 212256_at,207357_s_at,230906_at, | -0.04 | 5.86 | 6.03 | -1.17 | 2.47E-01 | 0.67 |
| ENSG00000176868.2 | RP11-334J6.7 | 212069_s_at, | 0.03 | 6.05 | 5.93 | 1.16 | 2.48E-01 | 0.67 |
| ENSG00000176728.7 | TTTY14 | 1560395_at,224003_at, | 0.03 | 3.59 | 3.52 | 1.16 | 2.47E-01 | 0.67 |
| ENSG00000232878.3 | DPYD-AS1 | 1566965_at,1566966_at,212738_at,236699_at,234160_at,  1560137_at,233698_at, | 0.02 | 4.29 | 4.25 | 1.16 | 2.50E-01 | 0.67 |
| ENSG00000223653.5 | RP11-131L23.1 | 209094_at,206837_at, | -0.02 | 6.44 | 6.54 | -1.14 | 2.56E-01 | 0.67 |
| ENSG00000203709.9 | C1orf132 | 232430_at, | 0.02 | 3.72 | 3.66 | 1.15 | 2.54E-01 | 0.67 |
| ENSG00000233246.1 | RP11-415J8.5 | 237396_at,210148_at, | 0.02 | 3.85 | 3.80 | 1.14 | 2.58E-01 | 0.67 |
| ENSG00000130600.15 | H19 | 224646_x_at,217723_x_at,224348_s_at, | -0.04 | 6.18 | 6.37 | -1.14 | 2.56E-01 | 0.67 |
| ENSG00000258344.1 | RP11-968A15.8 | 200016_x_at,222040_at,221919_at, | -0.01 | 12.25 | 12.35 | -1.16 | 2.51E-01 | 0.67 |
| ENSG00000275585.1 | CH17-118O6.3 | 205660_at,219022_at, | 0.07 | 5.38 | 5.12 | 1.15 | 2.53E-01 | 0.67 |
| ENSG00000233610.1 | LINC00462 | 241118_at, | 0.03 | 2.75 | 2.70 | 1.15 | 2.56E-01 | 0.67 |
| ENSG00000259354.5 | RP11-519G16.3 | 227193_at, | -0.04 | 6.15 | 6.32 | -1.15 | 2.53E-01 | 0.67 |
| ENSG00000262769.1 | RP11-1113L8.1 | 219525_at, | -0.06 | 3.46 | 3.60 | -1.15 | 2.55E-01 | 0.67 |
| ENSG00000279296.1 | RP11-609D21.3 | 228617_at, | 0.04 | 8.32 | 8.07 | 1.16 | 2.51E-01 | 0.67 |
| ENSG00000272780.5 | RP11-822E23.8 | 228146_at, | 0.02 | 4.57 | 4.50 | 1.15 | 2.54E-01 | 0.67 |
| ENSG00000263218.2 | CTD-2561B21.7 | 218743_at, | 0.02 | 6.62 | 6.52 | 1.14 | 2.56E-01 | 0.67 |
| ENSG00000261627.1 | RP11-91I8.1 | 1560863_a_at, | -0.03 | 2.87 | 2.92 | -1.14 | 2.56E-01 | 0.67 |
| ENSG00000278239.1 | AC020956.3 | 1569772_x_at, | -0.02 | 2.46 | 2.49 | -1.16 | 2.49E-01 | 0.67 |
| ENSG00000261428.2 | RP11-16P6.1 | 1556081_at,1556082_a_at, | 0.02 | 2.95 | 2.90 | 1.14 | 2.58E-01 | 0.67 |
| ENSG00000272902.2 | RP11-299H21.1 | 237543_at, | 0.03 | 3.24 | 3.18 | 1.16 | 2.50E-01 | 0.67 |
| ENSG00000272874.1 | RP5-1103G7.10 | 1561461_at, | 0.02 | 2.93 | 2.88 | 1.15 | 2.52E-01 | 0.67 |
| ENSG00000243629.1 | LINC00880 | 1561402_at, | 0.02 | 4.03 | 3.98 | 1.15 | 2.55E-01 | 0.67 |
| ENSG00000260261.1 | RP11-480A16.1 | 1556103_at, | -0.03 | 6.41 | 6.54 | -1.14 | 2.55E-01 | 0.67 |
| ENSG00000248019.2 | FAM13A-AS1 | 1558711_at, | -0.03 | 4.86 | 4.96 | -1.15 | 2.54E-01 | 0.67 |
| ENSG00000250658.1 | RP11-138B4.1 | 1561323_at, | -0.03 | 3.41 | 3.47 | -1.14 | 2.58E-01 | 0.67 |
| ENSG00000254363.6 | CTB-131B5.5 | 243251_at, | 0.03 | 3.48 | 3.41 | 1.16 | 2.50E-01 | 0.67 |
| ENSG00000246792.2 | RP11-68L18.1 | 1558440_at, | 0.03 | 3.11 | 3.05 | 1.14 | 2.58E-01 | 0.67 |
| ENSG00000246145.1 | RRS1-AS1 | 237729_at, | -0.03 | 2.53 | 2.57 | -1.15 | 2.51E-01 | 0.67 |
| ENSG00000261449.1 | RP11-589C21.5 | 1561579_at, | 0.02 | 3.86 | 3.81 | 1.14 | 2.56E-01 | 0.67 |
| ENSG00000237836.5 | PHKA2-AS1 | 209439_s_at, | -0.03 | 6.33 | 6.45 | -1.16 | 2.50E-01 | 0.67 |
| ENSG00000235437.7 | LINC01278 | 216838_at,228654_at, | -0.02 | 5.12 | 5.21 | -1.14 | 2.58E-01 | 0.67 |
| ENSG00000233864.7 | TTTY15 | 214983_at, | 0.02 | 2.71 | 2.66 | 1.14 | 2.57E-01 | 0.67 |
| ENSG00000227907.1 | RP11-102C16.3 | 205929_at, | -0.02 | 4.56 | 4.63 | -1.13 | 2.60E-01 | 0.67 |
| ENSG00000270094.1 | RP11-245P10.8 | 238797_at, | 0.04 | 4.72 | 4.57 | 1.14 | 2.59E-01 | 0.67 |
| ENSG00000260464.1 | RP4-561L24.3 | 1557431_at, | -0.02 | 2.60 | 2.65 | -1.13 | 2.60E-01 | 0.67 |
| ENSG00000269102.1 | CTD-2525I3.5 | 227284_at, | 0.02 | 7.14 | 7.03 | 1.13 | 2.61E-01 | 0.67 |
| ENSG00000230725.5 | RP4-738P15.1 | 1561978_at, | 0.02 | 3.88 | 3.82 | 1.13 | 2.60E-01 | 0.67 |
| ENSG00000227599.1 | RP4-753D4.2 | 1556921_at, | 0.02 | 2.77 | 2.74 | 1.13 | 2.59E-01 | 0.67 |
| ENSG00000249568.1 | RP11-234O6.2 | 232010_at, | -0.06 | 2.58 | 2.69 | -1.13 | 2.61E-01 | 0.67 |
| ENSG00000223360.1 | AC096559.2 | 242135_at, | 0.13 | 3.68 | 3.38 | 1.13 | 2.61E-01 | 0.67 |
| ENSG00000256116.1 | RP11-783K16.14 | 228622_s_at,223372_at,223371_s_at, | 0.02 | 4.83 | 4.77 | 1.13 | 2.63E-01 | 0.67 |
| ENSG00000235038.1 | RP11-393I23.4 | 1557328_at, | -0.03 | 2.47 | 2.52 | -1.13 | 2.63E-01 | 0.67 |
| ENSG00000275552.1 | CH17-302M23.1 | 1557857_a_at,1557856_at, | -0.02 | 2.72 | 2.75 | -1.12 | 2.63E-01 | 0.67 |
| ENSG00000274245.1 | RP11-357P18.2 | 1556107_at, | -0.03 | 3.72 | 3.79 | -1.12 | 2.64E-01 | 0.67 |
| ENSG00000249326.1 | CTD-2194D22.4 | 1562811_at, | 0.03 | 2.76 | 2.71 | 1.12 | 2.64E-01 | 0.67 |
| ENSG00000260941.1 | LINC00622 | 1558404_at, | -0.06 | 3.82 | 3.99 | -1.12 | 2.66E-01 | 0.67 |
| ENSG00000258702.1 | RP11-433J8.1 | 1556749_at, | -0.02 | 3.21 | 3.26 | -1.12 | 2.66E-01 | 0.67 |
| ENSG00000259462.2 | CPEB1-AS1 | 1570032_at, | -0.04 | 3.17 | 3.25 | -1.12 | 2.66E-01 | 0.67 |
| ENSG00000269696.1 | AC007228.11 | 240565_at,231943_at,240034_at,234937_x_at, | 0.02 | 3.63 | 3.58 | 1.12 | 2.65E-01 | 0.67 |
| ENSG00000222041.10 | LINC00152 | 236595_at,1558837_a_at,1560480_at, | -0.02 | 5.44 | 5.50 | -1.12 | 2.66E-01 | 0.67 |
| ENSG00000224660.1 | SH3BP5-AS1 | 201811_x_at, | -0.03 | 8.19 | 8.36 | -1.12 | 2.66E-01 | 0.67 |
| ENSG00000273033.2 | RP11-67L2.2 | 229699_at, | -0.03 | 6.03 | 6.18 | -1.12 | 2.67E-01 | 0.67 |
| ENSG00000236671.7 | PRKG1-AS1 | 1561558_at, | -0.03 | 2.69 | 2.75 | -1.11 | 2.68E-01 | 0.67 |
| ENSG00000235862.2 | RP11-338C15.5 | 1552383_at, | 0.02 | 6.01 | 5.93 | 1.10 | 2.72E-01 | 0.68 |
| ENSG00000232913.7 | PLCE1-AS2 | 1562826_at, | -0.03 | 3.44 | 3.51 | -1.11 | 2.72E-01 | 0.68 |
| ENSG00000279148.1 | RP11-135F9.4 | 1562098_at, | 0.02 | 2.99 | 2.95 | 1.11 | 2.70E-01 | 0.68 |
| ENSG00000279173.1 | RP11-318G8.4 | 1562932_at, | -0.02 | 2.85 | 2.89 | -1.11 | 2.71E-01 | 0.68 |
| ENSG00000233694.5 | AC007365.1 | 1562776_at, | 0.02 | 4.10 | 4.04 | 1.11 | 2.72E-01 | 0.68 |
| ENSG00000243902.6 | ELFN2 | 1560713_a_at,1559072_a_at, | -0.02 | 3.52 | 3.56 | -1.11 | 2.71E-01 | 0.68 |
| ENSG00000250456.1 | AC006552.1 | 1562540_at, | -0.03 | 3.71 | 3.78 | -1.11 | 2.72E-01 | 0.68 |
| ENSG00000232284.7 | GNG12-AS1 | 235664_at,215506_s_at,231494_at,225988_at,218878_s_at,  211004_s_at,243105_at,222759_at,1565974_at,221111_at,  216476_at,242737_at,1570166_a_at,1558418_at,1568600_at,  211112_at,210556_at,228379_at,204428_s_at,210555_s_at,  213141_at,212910_at,203785_s_at,201088_at, | 0.01 | 5.14 | 5.11 | 1.09 | 2.78E-01 | 0.68 |
| ENSG00000269176.2 | RP11-727F15.12 | 222053_at, | -0.02 | 3.30 | 3.36 | -1.10 | 2.75E-01 | 0.68 |
| ENSG00000231128.5 | RP5-1073O3.2 | 205115_s_at,1598_g_at", | -0.03 | 5.15 | 5.26 | -1.10 | 2.74E-01 | 0.68 |
| ENSG00000258048.1 | RP11-530C5.1 | 201603_at, | -0.03 | 8.06 | 8.21 | -1.09 | 2.77E-01 | 0.68 |
| ENSG00000230114.1 | RP11-141A19.2 | 223527_s_at, | -0.02 | 5.38 | 5.47 | -1.10 | 2.75E-01 | 0.68 |
| ENSG00000259736.1 | CRTC3-AS1 | 1569833_at,218648_at, | -0.02 | 8.38 | 8.52 | -1.10 | 2.73E-01 | 0.68 |
| ENSG00000260018.1 | RP11-505K9.1 | 230591_at, | 0.05 | 4.26 | 4.12 | 1.09 | 2.78E-01 | 0.68 |
| ENSG00000260268.5 | LINC00919 | 231627_at, | 0.03 | 3.37 | 3.30 | 1.09 | 2.76E-01 | 0.68 |
| ENSG00000267254.5 | ZNF790-AS1 | 1558809_s_at,235779_at, | -0.03 | 4.80 | 4.89 | -1.09 | 2.77E-01 | 0.68 |
| ENSG00000253392.2 | AC006277.2 | 240918_at,1554628_at, | 0.03 | 4.43 | 4.35 | 1.10 | 2.74E-01 | 0.68 |
| ENSG00000228340.5 | MIR646HG | 236846_at,1568742_at,1562051_at,237805_at,237806_s_at, | 0.03 | 3.41 | 3.35 | 1.10 | 2.73E-01 | 0.68 |
| ENSG00000270207.1 | RP11-157E16.1 | 224116_at, | 0.02 | 2.65 | 2.61 | 1.09 | 2.78E-01 | 0.68 |
| ENSG00000242049.1 | DNAJB8-AS1 | 1562740_at, | 0.03 | 3.01 | 2.95 | 1.10 | 2.75E-01 | 0.68 |
| ENSG00000180769.8 | WDFY3-AS2 | 1562953_s_at, | -0.03 | 3.56 | 3.63 | -1.09 | 2.77E-01 | 0.68 |
| ENSG00000251249.1 | RP11-73G16.1 | 237758_at, | 0.02 | 2.99 | 2.95 | 1.10 | 2.73E-01 | 0.68 |
| ENSG00000233070.1 | ZFY-AS1 | 207247_s_at,230760_at,207246_at, | 0.02 | 2.58 | 2.54 | 1.09 | 2.77E-01 | 0.68 |
| ENSG00000246331.2 | RP11-77I22.2 | 1560251_at, | -0.02 | 2.48 | 2.51 | -1.09 | 2.79E-01 | 0.68 |
| ENSG00000225521.1 | AC005237.4 | 1557437_a_at, | -0.06 | 2.73 | 2.85 | -1.09 | 2.80E-01 | 0.68 |
| ENSG00000227210.1 | AC079145.4 | 206091_at,209684_at, | -0.03 | 5.85 | 5.98 | -1.08 | 2.82E-01 | 0.69 |
| ENSG00000272589.1 | ZSWIM8-AS1 | 214867_at,216345_at, | 0.02 | 5.01 | 4.95 | 1.08 | 2.83E-01 | 0.69 |
| ENSG00000266993.3 | RP4-657D16.3 | 242994_at, | 0.02 | 4.43 | 4.37 | 1.08 | 2.85E-01 | 0.69 |
| ENSG00000258317.1 | RP11-603J24.5 | 227430_at,240677_at, | -0.01 | 5.57 | 5.63 | -1.08 | 2.85E-01 | 0.69 |
| ENSG00000179818.13 | PCBP1-AS1 | 1557727_at, | -0.02 | 3.15 | 3.21 | -1.08 | 2.85E-01 | 0.69 |
| ENSG00000197670.6 | RP4-724E16.2 | 215861_at, | 0.02 | 4.63 | 4.55 | 1.07 | 2.85E-01 | 0.69 |
| ENSG00000233067.2 | PTCHD1-AS | 1552967_at, | 0.02 | 3.33 | 3.29 | 1.08 | 2.84E-01 | 0.69 |
| ENSG00000259291.2 | RP11-617F23.1 | 39891_at,37590_g_at,213657_s_at, | -0.02 | 5.83 | 5.93 | -1.07 | 2.86E-01 | 0.69 |
| ENSG00000254154.8 | RP4-798P15.3 | 228150_at, | -0.05 | 4.60 | 4.76 | -1.07 | 2.87E-01 | 0.69 |
| ENSG00000246740.2 | PLA2G4E-AS1 | 1557863_at,1557864_x_at, | 0.02 | 2.70 | 2.67 | 1.07 | 2.88E-01 | 0.69 |
| ENSG00000229051.1 | RP5-952N6.1 | 244711_at,1564158_a_at, | 0.02 | 5.43 | 5.36 | 1.07 | 2.87E-01 | 0.69 |
| ENSG00000225667.1 | NA | 218824_at, | 0.08 | 5.50 | 5.19 | 1.07 | 2.88E-01 | 0.69 |
| ENSG00000260803.1 | Z84812.4 | 217212_s_at, | 0.03 | 4.12 | 4.05 | 1.07 | 2.88E-01 | 0.69 |
| ENSG00000278309.1 | NA | 1556114_a_at, | 0.02 | 4.65 | 4.59 | 1.06 | 2.90E-01 | 0.69 |
| ENSG00000279033.1 | RP11-500C12.1 | 218152_at, | -0.03 | 6.24 | 6.39 | -1.06 | 2.90E-01 | 0.69 |
| ENSG00000259661.1 | AC068831.15 | 202032_s_at, | -0.01 | 7.31 | 7.38 | -1.06 | 2.90E-01 | 0.69 |
| ENSG00000261749.2 | RP11-152O14.1 | 240894_at, | 0.02 | 2.93 | 2.88 | 1.07 | 2.89E-01 | 0.69 |
| ENSG00000248115.1 | RP11-752D24.2 | 1561314_at,1560383_at, | 0.01 | 3.09 | 3.06 | 1.06 | 2.91E-01 | 0.69 |
| ENSG00000253736.1 | RP11-779O18.3 | 201041_s_at,201044_x_at, | -0.03 | 6.91 | 7.05 | -1.06 | 2.91E-01 | 0.69 |
| ENSG00000179840.5 | PIK3CD-AS1 | 1559638_at,240572_s_at, | 0.02 | 6.22 | 6.15 | 1.05 | 2.95E-01 | 0.69 |
| ENSG00000281937.1 | RP3-510D11.4 | 1557757_at, | 0.02 | 4.87 | 4.81 | 1.06 | 2.94E-01 | 0.69 |
| ENSG00000224596.7 | ZMIZ1-AS1 | 1563745_a_at, | -0.03 | 4.66 | 4.76 | -1.06 | 2.93E-01 | 0.69 |
| ENSG00000234608.7 | MAPKAPK5-AS1 | 64432_at, | -0.04 | 3.06 | 3.13 | -1.06 | 2.92E-01 | 0.69 |
| ENSG00000258136.1 | RP11-864J10.4 | 1565839_a_at,1565838_at,1565840_at, | -0.01 | 2.74 | 2.77 | -1.05 | 2.95E-01 | 0.69 |
| ENSG00000257839.1 | RP11-290L1.2 | 1556730_at, | -0.02 | 2.93 | 2.97 | -1.05 | 2.94E-01 | 0.69 |
| ENSG00000189223.13 | PAX8-AS1 | 209552_at,244524_at,228425_at,213917_at, | 0.03 | 4.71 | 4.62 | 1.05 | 2.95E-01 | 0.69 |
| ENSG00000226143.1 | RP1-138B7.5 | 213837_at, | -0.02 | 4.50 | 4.56 | -1.06 | 2.93E-01 | 0.69 |
| ENSG00000244040.5 | IL12A-AS1 | 1560725_at, | 0.03 | 3.31 | 3.25 | 1.06 | 2.92E-01 | 0.69 |
| ENSG00000281202.2 | LINC01097 | 1562683_a_at, | -0.02 | 2.86 | 2.91 | -1.05 | 2.94E-01 | 0.69 |
| ENSG00000250328.5 | MGC32805 | 237833_s_at,237834_at, | 0.02 | 3.39 | 3.34 | 1.06 | 2.94E-01 | 0.69 |
| ENSG00000235954.6 | TTC28-AS1 | 215146_s_at,1558889_at, | -0.02 | 4.70 | 4.77 | -1.05 | 2.95E-01 | 0.69 |
| ENSG00000232973.11 | CYP1B1-AS1 | 1553829_at,216183_at,1561393_at,1569818_at,225024_at,  1569790_at,1552468_a_at,204746_s_at,221178_at,202027_at, | 0.01 | 4.52 | 4.48 | 1.05 | 2.96E-01 | 0.69 |
| ENSG00000253123.3 | RP11-527N22.1 | 1557540_at, | -0.02 | 4.06 | 4.11 | -1.05 | 2.96E-01 | 0.69 |
| ENSG00000232650.5 | RP5-834N19.1 | 224338_s_at,224286_at,224947_at,223499_at, | 0.01 | 5.81 | 5.76 | 1.05 | 2.98E-01 | 0.70 |
| ENSG00000258199.1 | RP11-977G19.5 | 1561973_at,201320_at, | -0.02 | 4.70 | 4.75 | -1.05 | 2.98E-01 | 0.70 |
| ENSG00000260565.6 | CTD-2270P14.1 | 1563629_a_at,1561942_x_at, | -0.03 | 4.82 | 4.91 | -1.05 | 2.98E-01 | 0.70 |
| ENSG00000280138.1 | RP11-463O12.5 | 236889_at, | -0.03 | 5.00 | 5.12 | -1.04 | 2.99E-01 | 0.70 |
| ENSG00000261025.1 | RP11-84D1.2 | 1561318_at,243855_at, | -0.01 | 3.51 | 3.55 | -1.04 | 3.02E-01 | 0.70 |
| ENSG00000227051.5 | C14orf132 | 231859_at, | -0.03 | 4.60 | 4.70 | -1.04 | 3.02E-01 | 0.70 |
| ENSG00000259133.5 | RP11-1085N6.3 | 1561919_at, | 0.03 | 4.02 | 3.94 | 1.04 | 3.01E-01 | 0.70 |
| ENSG00000247982.6 | LINC00926 | 230648_at, | 0.02 | 5.66 | 5.59 | 1.04 | 3.02E-01 | 0.70 |
| ENSG00000259336.1 | RP11-323I15.5 | 242142_at, | -0.04 | 4.48 | 4.61 | -1.04 | 3.03E-01 | 0.70 |
| ENSG00000234653.1 | AC079117.1 | 1570273_at, | -0.02 | 2.80 | 2.84 | -1.04 | 3.03E-01 | 0.70 |
| ENSG00000247199.3 | RP11-373N22.3 | 1557873_at, | -0.02 | 3.10 | 3.15 | -1.04 | 3.01E-01 | 0.70 |
| ENSG00000278921.2 | EPB41L4A-AS2 | 220364_at, | 0.04 | 3.85 | 3.76 | 1.04 | 3.02E-01 | 0.70 |
| ENSG00000261432.1 | LINC01613 | 1561278_at, | 0.03 | 3.10 | 3.02 | 1.04 | 3.02E-01 | 0.70 |
| ENSG00000250602.5 | RP11-517I3.1 | 238049_at, | 0.06 | 4.72 | 4.52 | 1.03 | 3.04E-01 | 0.70 |
| ENSG00000253394.5 | LINC00534 | 234087_at, | -0.04 | 3.18 | 3.26 | -1.03 | 3.04E-01 | 0.70 |
| ENSG00000228352.2 | RP11-537H15.3 | 224789_at, | 0.02 | 7.92 | 7.80 | 1.03 | 3.04E-01 | 0.70 |
| ENSG00000236817.5 | RP11-978I15.10 | 1552908_at, | -0.02 | 2.65 | 2.68 | -1.03 | 3.06E-01 | 0.70 |
| ENSG00000261460.1 | RP11-106M3.3 | 1556426_at, | 0.02 | 3.41 | 3.35 | 1.03 | 3.06E-01 | 0.70 |
| ENSG00000236449.1 | AC018890.6 | 211940_x_at,231182_at,213826_s_at,202663_at,  213828_x_at, | 0.01 | 7.66 | 7.59 | 1.03 | 3.06E-01 | 0.70 |
| ENSG00000248724.6 | NPHP3-AS1 | 1569882_at, | -0.04 | 2.98 | 3.06 | -1.03 | 3.06E-01 | 0.70 |
| ENSG00000261790.1 | AC005606.14 | 204660_at, | 0.02 | 6.23 | 6.15 | 1.03 | 3.07E-01 | 0.70 |
| ENSG00000265394.1 | RP11-1148O4.2 | 1558301_a_at,1558300_at, | -0.02 | 2.79 | 2.84 | -1.02 | 3.08E-01 | 0.70 |
| ENSG00000255125.1 | RP11-685M7.5 | 200004_at, | -0.02 | 11.27 | 11.40 | -1.02 | 3.10E-01 | 0.71 |
| ENSG00000239994.2 | RP11-169N13.4 | 1555719_a_at, | -0.02 | 2.81 | 2.85 | -1.02 | 3.11E-01 | 0.71 |
| ENSG00000260278.1 | RP11-109G23.3 | 1562036_at, | 0.02 | 5.36 | 5.29 | 1.02 | 3.10E-01 | 0.71 |
| ENSG00000238287.1 | RP11-656D10.3 | 1569107_s_at, | 0.03 | 3.11 | 3.05 | 1.02 | 3.13E-01 | 0.71 |
| ENSG00000254443.1 | RP11-304C12.3 | 1557007_a_at, | 0.03 | 3.59 | 3.52 | 1.01 | 3.13E-01 | 0.71 |
| ENSG00000250986.1 | AC141928.1 | 1559713_at, | 0.02 | 4.15 | 4.10 | 1.02 | 3.12E-01 | 0.71 |
| ENSG00000246859.2 | STARD4-AS1 | 1564789_at,222344_at,201310_s_at, | -0.03 | 5.13 | 5.23 | -1.01 | 3.13E-01 | 0.71 |
| ENSG00000253553.5 | RP11-586K2.1 | 1569831_at, | 0.03 | 2.56 | 2.52 | 1.02 | 3.12E-01 | 0.71 |
| ENSG00000250258.1 | CTC-431G16.2 | 233520_s_at, | -0.04 | 5.75 | 5.93 | -1.01 | 3.14E-01 | 0.71 |
| ENSG00000279352.1 | RP11-411B10.7 | 224284_x_at, | 0.02 | 7.54 | 7.42 | 1.01 | 3.14E-01 | 0.71 |
| ENSG00000246523.7 | RP11-736K20.6 | 1561539_at, | 0.02 | 4.14 | 4.07 | 1.01 | 3.16E-01 | 0.71 |
| ENSG00000237435.8 | NA | 229596_at,237967_at,206643_at,235749_at,1555560_at, | -0.03 | 3.23 | 3.28 | -1.01 | 3.16E-01 | 0.71 |
| ENSG00000253837.1 | RP11-177H13.2 | 1562263_at, | -0.02 | 4.41 | 4.46 | -1.01 | 3.16E-01 | 0.71 |
| ENSG00000261578.1 | RP11-21L23.2 | 237031_at, | 0.04 | 5.03 | 4.90 | 1.00 | 3.19E-01 | 0.71 |
| ENSG00000271259.1 | RP11-1109F11.5 | 236521_at, | -0.04 | 3.62 | 3.72 | -1.00 | 3.18E-01 | 0.71 |
| ENSG00000173867.8 | RP11-97O12.7 | 215919_s_at, | 0.02 | 3.38 | 3.33 | 1.00 | 3.18E-01 | 0.71 |
| ENSG00000267274.1 | CTD-2006C1.12 | 223590_at, | -0.03 | 6.32 | 6.46 | -1.00 | 3.18E-01 | 0.71 |
| ENSG00000280059.1 | RP11-401I19.2 | 1561239_at, | -0.02 | 2.59 | 2.63 | -1.00 | 3.19E-01 | 0.71 |
| ENSG00000215196.4 | AC091878.1 | 1553335_x_at, | 0.03 | 3.42 | 3.35 | 1.00 | 3.18E-01 | 0.71 |
| ENSG00000260886.1 | TAT-AS1 | 206916_x_at, | 0.03 | 3.68 | 3.61 | 1.00 | 3.20E-01 | 0.71 |
| ENSG00000266560.5 | RP11-1007I13.4 | 1553585_a_at, | 0.02 | 3.33 | 3.29 | 1.00 | 3.20E-01 | 0.71 |
| ENSG00000247853.2 | RP5-940J5.6 | 1560419_at, | 0.02 | 2.65 | 2.62 | 1.00 | 3.22E-01 | 0.72 |
| ENSG00000256362.1 | RP11-955H22.3 | 1563135_at, | -0.02 | 2.51 | 2.54 | -0.99 | 3.24E-01 | 0.72 |
| ENSG00000280415.1 | RP4-800O15.2 | 237150_at, | 0.02 | 2.65 | 2.60 | 0.99 | 3.25E-01 | 0.72 |
| ENSG00000268006.1 | PTOV1-AS1 | 233745_at, | 0.02 | 4.22 | 4.16 | 0.99 | 3.24E-01 | 0.72 |
| ENSG00000261829.1 | RP11-223I10.1 | 1556464_a_at, | 0.02 | 5.94 | 5.86 | 0.99 | 3.25E-01 | 0.72 |
| ENSG00000230773.6 | AC079807.4 | 237740_at,231611_at, | 0.02 | 3.88 | 3.83 | 0.99 | 3.25E-01 | 0.72 |
| ENSG00000260213.5 | RP11-303E16.3 | 222118_at, | 0.04 | 3.39 | 3.29 | 0.98 | 3.27E-01 | 0.72 |
| ENSG00000266830.1 | CTD-2008P7.8 | 220654_at, | 0.02 | 3.64 | 3.59 | 0.98 | 3.27E-01 | 0.72 |
| ENSG00000224271.5 | RP11-191L9.4 | 1564446_at, | -0.02 | 3.34 | 3.39 | -0.98 | 3.27E-01 | 0.72 |
| ENSG00000277879.1 | RP11-129M16.4 | 229907_at, | -0.02 | 3.08 | 3.12 | -0.98 | 3.29E-01 | 0.72 |
| ENSG00000246877.1 | DNM1P35 | 1561249_a_at, | 0.02 | 4.41 | 4.36 | 0.98 | 3.30E-01 | 0.72 |
| ENSG00000267607.1 | CTD-2369P2.8 | 207194_s_at, | -0.02 | 3.69 | 3.76 | -0.98 | 3.30E-01 | 0.72 |
| ENSG00000274769.1 | RP11-493E12.3 | 244673_at, | -0.02 | 2.41 | 2.45 | -0.98 | 3.32E-01 | 0.72 |
| ENSG00000271320.1 | RP11-1152H14.1 | 241513_at, | -0.03 | 3.50 | 3.58 | -0.98 | 3.30E-01 | 0.72 |
| ENSG00000233993.1 | RP11-379J5.5 | 1561459_at, | -0.02 | 3.68 | 3.73 | -0.98 | 3.29E-01 | 0.72 |
| ENSG00000093100.13 | XXbac-B461K10.4 | 212715_s_at,231553_s_at, | 0.02 | 5.52 | 5.44 | 0.98 | 3.31E-01 | 0.72 |
| ENSG00000238195.1 | CTA-503F6.2 | 1563507_at, | -0.03 | 3.16 | 3.22 | -0.97 | 3.32E-01 | 0.72 |
| ENSG00000260572.1 | RP11-16N11.2 | 1556147_at,231390_at,1556148_s_at, | -0.01 | 3.46 | 3.49 | -0.97 | 3.32E-01 | 0.72 |
| ENSG00000241328.1 | RP11-331K15.1 | 1561525_at, | 0.02 | 2.61 | 2.58 | 0.98 | 3.29E-01 | 0.72 |
| ENSG00000236064.1 | RP6-191P20.4 | 209733_at, | 0.03 | 5.31 | 5.22 | 0.98 | 3.31E-01 | 0.72 |
| ENSG00000225889.7 | AC074289.1 | 234847_at,210341_at,1556269_at, | -0.07 | 3.57 | 3.73 | -0.97 | 3.33E-01 | 0.72 |
| ENSG00000255968.1 | RP11-513G19.1 | 240458_at,202662_s_at, | -0.04 | 5.67 | 5.83 | -0.97 | 3.34E-01 | 0.72 |
| ENSG00000250433.1 | CLSTN2-AS1 | 234443_at, | 0.02 | 3.78 | 3.72 | 0.97 | 3.36E-01 | 0.73 |
| ENSG00000225670.4 | CADM3-AS1 | 217442_at,216535_at,208335_s_at, | -0.03 | 4.55 | 4.64 | -0.96 | 3.38E-01 | 0.73 |
| ENSG00000231674.1 | LINC00410 | 1564287_at, | -0.02 | 3.69 | 3.74 | -0.96 | 3.39E-01 | 0.73 |
| ENSG00000259536.5 | RP11-111A22.1 | 1557627_at,1558648_at, | 0.02 | 3.14 | 3.11 | 0.96 | 3.41E-01 | 0.74 |
| ENSG00000260019.1 | RP11-218F4.1 | 1568904_at, | -0.02 | 3.30 | 3.35 | -0.95 | 3.42E-01 | 0.74 |
| ENSG00000260093.1 | RP11-1E4.1 | 1557825_at, | -0.02 | 3.33 | 3.38 | -0.95 | 3.42E-01 | 0.74 |
| ENSG00000261596.2 | CTB-31N19.3 | 230395_at, | -0.02 | 5.51 | 5.61 | -0.95 | 3.43E-01 | 0.74 |
| ENSG00000232684.1 | ATP11A-AS1 | 1556854_at,1561699_a_at, | -0.01 | 3.35 | 3.38 | -0.95 | 3.44E-01 | 0.74 |
| ENSG00000279203.1 | AC005785.5 | 1559259_at, | -0.04 | 3.64 | 3.75 | -0.95 | 3.45E-01 | 0.74 |
| ENSG00000260743.1 | RP11-255C15.3 | 215825_at, | -0.02 | 4.17 | 4.23 | -0.95 | 3.45E-01 | 0.74 |
| ENSG00000234132.2 | RP11-168O16.2 | 217515_s_at, | 0.02 | 4.31 | 4.26 | 0.94 | 3.47E-01 | 0.74 |
| ENSG00000227492.1 | RP11-316M21.6 | 229038_at, | 0.02 | 4.07 | 4.02 | 0.94 | 3.48E-01 | 0.74 |
| ENSG00000244968.6 | LIFR-AS1 | 1559986_at, | -0.02 | 2.48 | 2.51 | -0.94 | 3.49E-01 | 0.75 |
| ENSG00000231999.6 | FLJ27354 | 212978_at,215716_s_at,212930_at,229405_at,219707_at, | 0.01 | 6.14 | 6.09 | 0.94 | 3.51E-01 | 0.75 |
| ENSG00000257042.1 | RP11-993B23.3 | 211756_at, | -0.10 | 3.23 | 3.47 | -0.94 | 3.50E-01 | 0.75 |
| ENSG00000258891.1 | RP5-1021I20.5 | 209944_at,202010_s_at, | -0.01 | 8.30 | 8.39 | -0.94 | 3.52E-01 | 0.75 |
| ENSG00000178977.3 | LINC00324 | 230012_at, | -0.03 | 5.37 | 5.47 | -0.94 | 3.51E-01 | 0.75 |
| ENSG00000235070.3 | AC068138.1 | 1564701_at, | -0.02 | 3.60 | 3.65 | -0.94 | 3.50E-01 | 0.75 |
| ENSG00000226527.1 | AP000289.6 | 1569828_at, | -0.02 | 3.21 | 3.25 | -0.93 | 3.52E-01 | 0.75 |
| ENSG00000233360.4 | Z83844.1 | 213633_at, | 0.02 | 5.76 | 5.69 | 0.94 | 3.52E-01 | 0.75 |
| ENSG00000228839.5 | PIK3IP1-AS1 | 209431_s_at, | -0.02 | 7.34 | 7.45 | -0.94 | 3.52E-01 | 0.75 |
| ENSG00000243197.7 | TUSC7 | 1558601_at, | 0.02 | 2.73 | 2.68 | 0.93 | 3.52E-01 | 0.75 |
| ENSG00000260505.1 | RP11-410C4.5 | 1561683_at, | 0.01 | 4.34 | 4.30 | 0.93 | 3.55E-01 | 0.75 |
| ENSG00000279176.1 | RP11-43D4.3 | 1554615_at, | 0.02 | 3.72 | 3.67 | 0.92 | 3.58E-01 | 0.75 |
| ENSG00000258708.1 | SLC25A21-AS1 | 230307_at, | -0.03 | 3.12 | 3.18 | -0.93 | 3.55E-01 | 0.75 |
| ENSG00000263874.1 | LINC00672 | 238359_at, | 0.02 | 5.38 | 5.31 | 0.92 | 3.59E-01 | 0.75 |
| ENSG00000265496.3 | MIR1539 | 214291_at, | -0.03 | 6.34 | 6.47 | -0.92 | 3.58E-01 | 0.75 |
| ENSG00000267124.2 | CTD-3113P16.5 | 221863_at,44822_s_at, | 0.01 | 6.20 | 6.14 | 0.93 | 3.57E-01 | 0.75 |
| ENSG00000228016.1 | RAPGEF4-AS1 | 1563528_at, | -0.02 | 2.79 | 2.84 | -0.93 | 3.57E-01 | 0.75 |
| ENSG00000227028.6 | SLC8A1-AS1 | 207053_at,237268_at,243027_at,1558764_at,217092_x_at, | 0.01 | 4.43 | 4.40 | 0.93 | 3.56E-01 | 0.75 |
| ENSG00000223799.1 | IL10RB-AS1 | 230631_s_at,204786_s_at, | 0.02 | 5.37 | 5.29 | 0.93 | 3.56E-01 | 0.75 |
| ENSG00000279328.1 | RP11-203H19.2 | 220824_at, | 0.02 | 4.63 | 4.58 | 0.93 | 3.57E-01 | 0.75 |
| ENSG00000270959.1 | LPP-AS2 | 230996_at, | 0.02 | 3.43 | 3.39 | 0.92 | 3.59E-01 | 0.75 |
| ENSG00000272870.1 | RP11-798M19.6 | 1560460_at, | -0.02 | 2.59 | 2.62 | -0.93 | 3.55E-01 | 0.75 |
| ENSG00000250033.5 | SLC7A11-AS1 | 1562253_at, | -0.02 | 3.08 | 3.12 | -0.92 | 3.58E-01 | 0.75 |
| ENSG00000233006.6 | AC034220.3 | 230757_at, | -0.03 | 5.49 | 5.61 | -0.92 | 3.58E-01 | 0.75 |
| ENSG00000213468.3 | FIRRE | 1558791_at, | 0.04 | 3.45 | 3.36 | 0.92 | 3.59E-01 | 0.75 |
| ENSG00000258586.1 | RP5-1021I20.2 | 1557921_s_at, | -0.02 | 2.79 | 2.82 | -0.92 | 3.60E-01 | 0.75 |
| ENSG00000259941.1 | RP11-48G14.2 | 1566605_at, | -0.02 | 2.58 | 2.61 | -0.92 | 3.60E-01 | 0.75 |
| ENSG00000267009.6 | RP11-120M18.2 | 213836_s_at, | -0.03 | 7.57 | 7.73 | -0.92 | 3.60E-01 | 0.75 |
| ENSG00000265692.1 | RP13-516M14.4 | 1568899_at, | -0.02 | 2.92 | 2.96 | -0.92 | 3.61E-01 | 0.75 |
| ENSG00000233718.5 | MYCNOS | 216188_at, | 0.03 | 3.07 | 3.02 | 0.92 | 3.61E-01 | 0.75 |
| ENSG00000272610.1 | MAGI1-IT1 | 1561301_at, | 0.02 | 3.27 | 3.23 | 0.92 | 3.61E-01 | 0.75 |
| ENSG00000226746.2 | SMCR5 | 1553020_at, | 0.02 | 3.14 | 3.09 | 0.92 | 3.62E-01 | 0.75 |
| ENSG00000271941.1 | RP11-188P20.3 | 237942_at, | -0.04 | 4.20 | 4.33 | -0.92 | 3.62E-01 | 0.75 |
| ENSG00000263272.1 | CTC-524C5.2 | 1564301_a_at,1556088_at,1558719_s_at, | -0.02 | 3.74 | 3.79 | -0.91 | 3.63E-01 | 0.75 |
| ENSG00000237781.3 | RP11-54A4.2 | 220578_at, | -0.02 | 4.64 | 4.71 | -0.91 | 3.63E-01 | 0.75 |
| ENSG00000228697.1 | RP5-968D22.1 | 1559522_at, | -0.02 | 4.68 | 4.75 | -0.91 | 3.67E-01 | 0.75 |
| ENSG00000229953.1 | RP11-284F21.7 | 221623_at, | -0.01 | 3.10 | 3.13 | -0.91 | 3.66E-01 | 0.75 |
| ENSG00000255322.1 | NA | 1567591_at,1567590_at, | -0.01 | 3.60 | 3.64 | -0.90 | 3.68E-01 | 0.75 |
| ENSG00000245522.2 | RP11-540A21.2 | 1562606_a_at,1566830_at, | 0.02 | 4.06 | 4.01 | 0.91 | 3.66E-01 | 0.75 |
| ENSG00000225083.1 | GRTP1-AS1 | 1559654_s_at, | 0.02 | 3.53 | 3.49 | 0.91 | 3.68E-01 | 0.75 |
| ENSG00000280061.1 | CTD-2001J20.1 | 206403_at, | -0.02 | 3.33 | 3.38 | -0.91 | 3.67E-01 | 0.75 |
| ENSG00000235724.8 | AC009299.2 | 237609_at,236598_at, | -0.02 | 3.51 | 3.55 | -0.91 | 3.66E-01 | 0.75 |
| ENSG00000232044.6 | LINC01105 | 1553796_at,233970_s_at,1557334_a_at, | 0.01 | 3.85 | 3.82 | 0.90 | 3.68E-01 | 0.75 |
| ENSG00000228109.1 | MFI2-AS1 | 239521_at, | 0.02 | 4.42 | 4.36 | 0.90 | 3.68E-01 | 0.75 |
| ENSG00000280047.1 | CTC-463A16.1 | 1556636_at, | -0.01 | 3.69 | 3.73 | -0.90 | 3.68E-01 | 0.75 |
| ENSG00000257431.1 | RP11-263K4.3 | 1563656_at, | 0.02 | 3.16 | 3.13 | 0.90 | 3.69E-01 | 0.75 |
| ENSG00000263327.6 | TAPT1-AS1 | 240927_at, | -0.04 | 3.05 | 3.14 | -0.90 | 3.69E-01 | 0.75 |
| ENSG00000245904.3 | RP11-796E2.4 | 244429_at, | 0.02 | 6.30 | 6.21 | 0.89 | 3.73E-01 | 0.75 |
| ENSG00000268240.1 | RP11-678G14.4 | 1568894_at, | 0.02 | 4.15 | 4.10 | 0.90 | 3.72E-01 | 0.75 |
| ENSG00000267328.1 | AC002398.12 | 226304_at, | -0.03 | 4.22 | 4.29 | -0.90 | 3.72E-01 | 0.75 |
| ENSG00000228613.1 | AC144450.1 | 217240_at, | -0.02 | 4.91 | 4.98 | -0.90 | 3.73E-01 | 0.75 |
| ENSG00000244358.1 | RP11-88H10.2 | 241160_at, | -0.02 | 2.72 | 2.77 | -0.90 | 3.71E-01 | 0.75 |
| ENSG00000271993.1 | RP11-285J16.1 | 236500_at, | -0.03 | 4.03 | 4.12 | -0.90 | 3.71E-01 | 0.75 |
| ENSG00000245954.6 | RP11-18H21.1 | 1559205_s_at, | -0.02 | 4.01 | 4.07 | -0.90 | 3.71E-01 | 0.75 |
| ENSG00000272904.1 | RP11-392E22.11 | 1559313_at, | -0.02 | 2.87 | 2.91 | -0.90 | 3.73E-01 | 0.75 |
| ENSG00000224950.2 | RP5-1086K13.1 | 240661_at, | 0.02 | 3.50 | 3.45 | 0.89 | 3.75E-01 | 0.76 |
| ENSG00000267342.1 | RP11-552F3.10 | 230726_at, | 0.02 | 4.03 | 3.98 | 0.89 | 3.77E-01 | 0.76 |
| ENSG00000229414.2 | KCNQ1-AS1 | 204487_s_at, | 0.02 | 6.00 | 5.92 | 0.89 | 3.78E-01 | 0.76 |
| ENSG00000259814.1 | RP11-300A12.2 | 1561487_at, | 0.01 | 3.43 | 3.40 | 0.89 | 3.78E-01 | 0.76 |
| ENSG00000266929.1 | RP11-400F19.8 | 215880_at, | 0.01 | 3.97 | 3.94 | 0.88 | 3.78E-01 | 0.76 |
| ENSG00000242861.1 | RP11-285F7.2 | 228549_at, | 0.03 | 6.93 | 6.80 | 0.88 | 3.81E-01 | 0.76 |
| ENSG00000255139.1 | AP000442.1 | 225466_at,225468_at, | -0.03 | 6.04 | 6.16 | -0.88 | 3.82E-01 | 0.76 |
| ENSG00000226472.7 | RP11-551L14.4 | 227610_at, | -0.02 | 4.81 | 4.89 | -0.88 | 3.81E-01 | 0.76 |
| ENSG00000223479.3 | RP4-788P17.1 | 214276_at,1561112_at,238940_at, | -0.02 | 3.32 | 3.36 | -0.88 | 3.82E-01 | 0.76 |
| ENSG00000230058.1 | RP11-172E9.2 | 1555264_a_at, | 0.02 | 3.41 | 3.37 | 0.88 | 3.83E-01 | 0.76 |
| ENSG00000259124.1 | RP11-187O7.3 | 1556156_at, | 0.02 | 2.78 | 2.75 | 0.87 | 3.85E-01 | 0.76 |
| ENSG00000261183.5 | RP11-532F12.5 | 242354_at, | 0.03 | 7.80 | 7.63 | 0.88 | 3.80E-01 | 0.76 |
| ENSG00000180422.3 | LINC00304 | 1564334_at, | -0.01 | 4.25 | 4.29 | -0.87 | 3.84E-01 | 0.76 |
| ENSG00000266846.1 | RP11-793A3.2 | 1554699_at, | -0.03 | 3.04 | 3.10 | -0.88 | 3.81E-01 | 0.76 |
| ENSG00000231609.5 | AC009501.4 | 212653_s_at, | -0.02 | 7.71 | 7.83 | -0.87 | 3.85E-01 | 0.76 |
| ENSG00000241770.1 | RP11-555M1.3 | 238276_at, | -0.01 | 4.43 | 4.47 | -0.88 | 3.83E-01 | 0.76 |
| ENSG00000248165.1 | RP11-44F21.2 | 241124_at, | -0.04 | 2.93 | 3.02 | -0.87 | 3.84E-01 | 0.76 |
| ENSG00000269506.2 | RP11-571I18.5 | 1562079_at, | -0.02 | 2.37 | 2.39 | -0.87 | 3.84E-01 | 0.76 |
| ENSG00000247828.7 | TMEM161B-AS1 | 241035_s_at, | 0.02 | 2.79 | 2.75 | 0.87 | 3.84E-01 | 0.76 |
| ENSG00000233569.1 | RP11-500B12.1 | 1570206_at, | -0.02 | 3.06 | 3.10 | -0.88 | 3.83E-01 | 0.76 |
| ENSG00000229582.3 | RP11-423C15.3 | 220752_at, | -0.02 | 2.74 | 2.78 | -0.88 | 3.83E-01 | 0.76 |
| ENSG00000203930.10 | LINC00632 | 1557133_at, | -0.02 | 2.64 | 2.67 | -0.87 | 3.85E-01 | 0.76 |
| ENSG00000259703.5 | LINC00593 | 1554716_s_at, | -0.02 | 3.20 | 3.24 | -0.87 | 3.89E-01 | 0.76 |
| ENSG00000276980.1 | CTD-3128G10.7 | 221159_at, | 0.03 | 3.49 | 3.43 | 0.87 | 3.89E-01 | 0.76 |
| ENSG00000239440.5 | RP11-260O18.1 | 1569789_at, | -0.02 | 3.09 | 3.14 | -0.87 | 3.89E-01 | 0.76 |
| ENSG00000249375.7 | CASC11 | 1561232_at, | -0.03 | 3.20 | 3.27 | -0.87 | 3.89E-01 | 0.76 |
| ENSG00000253521.1 | HPYR1 | 1566917_at, | 0.02 | 3.54 | 3.49 | 0.87 | 3.89E-01 | 0.76 |
| ENSG00000237365.1 | RP11-334N17.1 | 233042_at, | -0.02 | 3.15 | 3.19 | -0.86 | 3.90E-01 | 0.76 |
| ENSG00000230990.1 | RP4-734C18.1 | 1564049_at, | -0.02 | 2.97 | 3.01 | -0.86 | 3.90E-01 | 0.76 |
| ENSG00000214248.2 | CTD-3193O13.12 | 239433_at, | -0.04 | 5.49 | 5.64 | -0.86 | 3.92E-01 | 0.77 |
| ENSG00000226698.1 | RP1-50O24.6 | 235058_at, | -0.02 | 4.89 | 4.97 | -0.86 | 3.93E-01 | 0.77 |
| ENSG00000224645.1 | RP11-126K1.8 | 202963_at, | -0.03 | 7.57 | 7.71 | -0.86 | 3.94E-01 | 0.77 |
| ENSG00000257815.5 | LINC01481 | 1561272_at, | -0.02 | 2.89 | 2.93 | -0.86 | 3.93E-01 | 0.77 |
| ENSG00000261728.1 | RP11-307O13.1 | 234538_at,234836_at, | 0.01 | 3.97 | 3.94 | 0.86 | 3.94E-01 | 0.77 |
| ENSG00000279091.1 | RP11-461F11.2 | 1562100_at, | -0.02 | 3.24 | 3.30 | -0.86 | 3.92E-01 | 0.77 |
| ENSG00000260708.1 | CTA-29F11.1 | 227044_at, | 0.03 | 5.98 | 5.87 | 0.86 | 3.94E-01 | 0.77 |
| ENSG00000240288.7 | GHRLOS | 239617_at, | 0.02 | 4.24 | 4.19 | 0.86 | 3.95E-01 | 0.77 |
| ENSG00000271895.2 | RP4-635E18.8 | 229111_at, | -0.03 | 6.08 | 6.21 | -0.85 | 3.98E-01 | 0.77 |
| ENSG00000260269.5 | CTD-2323K18.1 | 207438_s_at, | 0.02 | 6.91 | 6.83 | 0.85 | 3.97E-01 | 0.77 |
| ENSG00000262967.1 | RP11-294J22.6 | 211717_at, | 0.02 | 5.24 | 5.16 | 0.85 | 3.97E-01 | 0.77 |
| ENSG00000266844.1 | RP11-862L9.3 | 207323_s_at, | 0.01 | 5.06 | 5.02 | 0.85 | 3.97E-01 | 0.77 |
| ENSG00000251307.1 | RP11-506H20.1 | 1566046_at,1566047_at, | -0.02 | 2.96 | 2.99 | -0.85 | 3.98E-01 | 0.77 |
| ENSG00000236915.1 | RP4-651E10.4 | 234226_at,216572_at, | 0.01 | 3.79 | 3.76 | 0.85 | 3.99E-01 | 0.77 |
| ENSG00000262198.1 | RP11-124N19.3 | 229640_x_at, | 0.02 | 6.96 | 6.84 | 0.84 | 4.01E-01 | 0.77 |
| ENSG00000230393.1 | AC092667.2 | 1561086_at, | -0.02 | 2.99 | 3.04 | -0.84 | 4.01E-01 | 0.77 |
| ENSG00000180539.7 | C9orf139 | 210506_at, | 0.02 | 3.14 | 3.10 | 0.84 | 4.01E-01 | 0.77 |
| ENSG00000198547.8 | C20orf203 | 1556722_a_at, | 0.01 | 5.17 | 5.11 | 0.84 | 4.02E-01 | 0.77 |
| ENSG00000260090.1 | RP11-292D4.3 | 234448_at,234441_at, | -0.02 | 2.99 | 3.03 | -0.84 | 4.02E-01 | 0.77 |
| ENSG00000234917.2 | RP5-994D16.3 | 236142_at, | 0.02 | 3.28 | 3.24 | 0.84 | 4.04E-01 | 0.77 |
| ENSG00000254418.1 | RP11-21L19.1 | 213993_at,213994_s_at,209437_s_at, | -0.05 | 5.18 | 5.34 | -0.84 | 4.05E-01 | 0.77 |
| ENSG00000278338.4 | VWA8-AS1 | 1557646_at, | 0.02 | 2.53 | 2.50 | 0.84 | 4.03E-01 | 0.77 |
| ENSG00000261959.1 | RP11-893F2.14 | 1562729_at,1562730_a_at, | 0.02 | 3.66 | 3.62 | 0.84 | 4.03E-01 | 0.77 |
| ENSG00000271554.1 | RP4-665N4.8 | 221855_at, | 0.01 | 7.37 | 7.31 | 0.84 | 4.05E-01 | 0.77 |
| ENSG00000233684.2 | AC079779.6 | 240549_at, | -0.03 | 3.08 | 3.14 | -0.84 | 4.05E-01 | 0.77 |
| ENSG00000248464.1 | FGF10-AS1 | 1562691_at, | -0.02 | 3.06 | 3.10 | -0.83 | 4.06E-01 | 0.78 |
| ENSG00000247934.4 | RP11-967K21.1 | 240518_at, | -0.02 | 3.04 | 3.09 | -0.83 | 4.07E-01 | 0.78 |
| ENSG00000227354.6 | RBM26-AS1 | 1568807_a_at, | -0.04 | 3.34 | 3.43 | -0.83 | 4.10E-01 | 0.78 |
| ENSG00000239482.6 | RP11-90K6.1 | 1562997_a_at, | -0.01 | 2.25 | 2.27 | -0.83 | 4.11E-01 | 0.78 |
| ENSG00000272145.1 | NFYC-AS1 | 1558782_a_at, | 0.02 | 4.63 | 4.57 | 0.82 | 4.13E-01 | 0.78 |
| ENSG00000231964.1 | RP11-67C2.2 | 204446_s_at, | -0.04 | 7.22 | 7.43 | -0.82 | 4.12E-01 | 0.78 |
| ENSG00000278879.1 | AP000560.3 | 233066_at, | -0.03 | 2.87 | 2.93 | -0.82 | 4.13E-01 | 0.78 |
| ENSG00000171671.6 | SHANK2-AS3 | 224139_at, | 0.01 | 3.73 | 3.69 | 0.82 | 4.13E-01 | 0.78 |
| ENSG00000264968.1 | RP11-387H17.4 | 1557301_a_at, | 0.02 | 2.79 | 2.74 | 0.83 | 4.11E-01 | 0.78 |
| ENSG00000278986.1 | RP11-723J4.3 | 1558990_at, | -0.02 | 2.79 | 2.83 | -0.82 | 4.13E-01 | 0.78 |
| ENSG00000225335.3 | XXbac-B476C20.9 | 233269_at, | 0.02 | 3.08 | 3.05 | 0.82 | 4.13E-01 | 0.78 |
| ENSG00000245573.7 | BDNF-AS | 239367_at,244503_at, | -0.03 | 4.09 | 4.18 | -0.82 | 4.14E-01 | 0.78 |
| ENSG00000258782.3 | RP11-701B16.2 | 221138_s_at,221137_at, | -0.01 | 3.37 | 3.40 | -0.82 | 4.15E-01 | 0.78 |
| ENSG00000235665.5 | LINC00298 | 1562717_at,201580_s_at,216027_at, | 0.03 | 6.44 | 6.31 | 0.82 | 4.16E-01 | 0.78 |
| ENSG00000235304.1 | LINC01281 | 234887_at, | 0.02 | 3.66 | 3.60 | 0.82 | 4.16E-01 | 0.78 |
| ENSG00000260042.1 | CTD-2034I21.1 | 1563064_at, | 0.01 | 3.53 | 3.50 | 0.82 | 4.17E-01 | 0.78 |
| ENSG00000259248.5 | USP3-AS1 | 221654_s_at,1562738_a_at, | -0.01 | 5.02 | 5.07 | -0.81 | 4.17E-01 | 0.78 |
| ENSG00000270075.1 | RP11-127L20.5 | 225582_at, | -0.02 | 5.83 | 5.93 | -0.81 | 4.19E-01 | 0.79 |
| ENSG00000261063.1 | RP11-538I12.2 | 215301_at, | 0.02 | 2.56 | 2.53 | 0.81 | 4.19E-01 | 0.79 |
| ENSG00000225511.6 | LINC00475 | 233525_s_at,233524_at, | 0.01 | 4.09 | 4.05 | 0.81 | 4.20E-01 | 0.79 |
| ENSG00000231814.2 | LINC00210 | 1562802_at, | -0.02 | 2.56 | 2.58 | -0.81 | 4.22E-01 | 0.79 |
| ENSG00000225623.1 | AGBL4-IT1 | 230108_at,207347_at,1561017_at,1555103_s_at,1555102_at,  1561224_at,241039_at,200659_s_at, | 0.01 | 2.62 | 2.60 | 0.81 | 4.22E-01 | 0.79 |
| ENSG00000236213.1 | AC006369.2 | 209286_at,209287_s_at,209288_s_at,210454_s_at, | -0.03 | 4.74 | 4.83 | -0.81 | 4.21E-01 | 0.79 |
| ENSG00000224924.6 | LINC00320 | 1557481_a_at, | 0.02 | 2.65 | 2.62 | 0.81 | 4.22E-01 | 0.79 |
| ENSG00000272149.1 | RP11-627J17.1 | 227138_at, | -0.01 | 5.72 | 5.77 | -0.81 | 4.21E-01 | 0.79 |
| ENSG00000233098.8 | CCDC144NL-AS1 | 1564360_a_at,1564359_a_at,229669_at,1560099_at, | -0.02 | 2.78 | 2.82 | -0.80 | 4.24E-01 | 0.79 |
| ENSG00000248505.1 | RP11-319E12.1 | 238314_x_at, | 0.02 | 2.88 | 2.84 | 0.80 | 4.25E-01 | 0.79 |
| ENSG00000226053.1 | RP5-1070A16.1 | 227145_at, | 0.03 | 6.10 | 5.99 | 0.80 | 4.26E-01 | 0.79 |
| ENSG00000255345.1 | CTD-2337I7.1 | 1554957_at, | 0.02 | 3.38 | 3.33 | 0.80 | 4.27E-01 | 0.79 |
| ENSG00000255153.1 | TOLLIP-AS1 | 1555865_at, | -0.04 | 4.53 | 4.65 | -0.80 | 4.27E-01 | 0.79 |
| ENSG00000255399.3 | TBX5-AS1 | 239919_at, | -0.02 | 3.28 | 3.32 | -0.80 | 4.27E-01 | 0.79 |
| ENSG00000262294.1 | RP11-1260E13.2 | 244880_at, | 0.02 | 3.21 | 3.17 | 0.80 | 4.27E-01 | 0.79 |
| ENSG00000224165.5 | DNAJC27-AS1 | 234483_at,234801_s_at,224882_at,234484_s_at, | -0.01 | 4.95 | 4.99 | -0.80 | 4.28E-01 | 0.79 |
| ENSG00000273192.1 | CITF22-1A6.3 | 218444_at, | 0.01 | 5.72 | 5.66 | 0.79 | 4.29E-01 | 0.79 |
| ENSG00000152931.7 | PART1 | 215972_at,205834_s_at, | -0.02 | 3.90 | 3.94 | -0.80 | 4.28E-01 | 0.79 |
| ENSG00000233973.5 | NA | 1561213_at,220628_s_at,242064_at,237552_at, | 0.02 | 4.14 | 4.07 | 0.79 | 4.30E-01 | 0.79 |
| ENSG00000254246.1 | CTB-120L21.1 | 1555628_a_at, | -0.02 | 4.49 | 4.53 | -0.79 | 4.30E-01 | 0.79 |
| ENSG00000203876.9 | ADD3-AS1 | 222072_at, | 0.02 | 4.30 | 4.26 | 0.79 | 4.30E-01 | 0.79 |
| ENSG00000227217.1 | RP11-367J7.3 | 241278_at, | -0.03 | 2.77 | 2.82 | -0.79 | 4.31E-01 | 0.79 |
| ENSG00000279030.1 | RP11-212I21.3 | 1566678_at, | 0.02 | 4.13 | 4.08 | 0.79 | 4.32E-01 | 0.79 |
| ENSG00000251523.1 | RP11-724M22.1 | 1562281_at, | -0.02 | 2.80 | 2.83 | -0.79 | 4.32E-01 | 0.79 |
| ENSG00000224429.7 | LINC00539 | 236837_x_at,240436_at, | -0.03 | 4.27 | 4.35 | -0.79 | 4.34E-01 | 0.79 |
| ENSG00000268230.5 | CTD-2619J13.8 | 235769_at,232462_s_at, | 0.02 | 4.05 | 4.00 | 0.79 | 4.33E-01 | 0.79 |
| ENSG00000231563.1 | RP11-245P10.4 | 220279_at, | 0.02 | 4.44 | 4.38 | 0.78 | 4.35E-01 | 0.80 |
| ENSG00000264895.1 | RP11-421E14.2 | 1558449_at, | 0.04 | 3.18 | 3.09 | 0.78 | 4.35E-01 | 0.80 |
| ENSG00000236306.2 | LINC01241 | 1560836_at, | -0.02 | 3.39 | 3.43 | -0.78 | 4.35E-01 | 0.80 |
| ENSG00000256699.1 | RP11-669N7.3 | 1560810_at, | 0.02 | 2.96 | 2.93 | 0.78 | 4.37E-01 | 0.80 |
| ENSG00000273783.1 | CTD-2506P8.6 | 1559009_at, | -0.01 | 3.02 | 3.05 | -0.78 | 4.38E-01 | 0.80 |
| ENSG00000132832.9 | LINC01260 | 1556117_at, | -0.02 | 3.29 | 3.34 | -0.78 | 4.37E-01 | 0.80 |
| ENSG00000249464.5 | LINC01091 | 1563462_at,1561096_at, | 0.02 | 2.77 | 2.74 | 0.78 | 4.37E-01 | 0.80 |
| ENSG00000233047.5 | NA | 237909_at, | -0.02 | 3.86 | 3.90 | -0.78 | 4.38E-01 | 0.80 |
| ENSG00000174171.5 | RP11-23P13.6 | 237097_at,239472_at, | 0.01 | 3.27 | 3.24 | 0.78 | 4.39E-01 | 0.80 |
| ENSG00000228748.2 | RP13-39P12.3 | 229689_s_at, | 0.03 | 6.13 | 6.00 | 0.77 | 4.41E-01 | 0.80 |
| ENSG00000280426.1 | RP11-837J7.3 | 1556913_a_at,1556912_at, | -0.01 | 2.52 | 2.54 | -0.77 | 4.41E-01 | 0.80 |
| ENSG00000232307.1 | DAOA-AS1 | 1553024_at, | 0.01 | 2.51 | 2.49 | 0.78 | 4.40E-01 | 0.80 |
| ENSG00000254744.3 | CTD-3076O17.1 | 1552726_at, | -0.02 | 3.02 | 3.07 | -0.78 | 4.39E-01 | 0.80 |
| ENSG00000233766.7 | AC098617.1 | 233910_at,223557_s_at, | 0.01 | 2.68 | 2.67 | 0.78 | 4.40E-01 | 0.80 |
| ENSG00000231004.2 | CECR9 | 1567686_at,1567687_at, | -0.01 | 2.98 | 3.00 | -0.77 | 4.41E-01 | 0.80 |
| ENSG00000251248.1 | RP11-223C24.2 | 1564014_at, | 0.02 | 2.86 | 2.82 | 0.77 | 4.43E-01 | 0.80 |
| ENSG00000225937.1 | PCA3 | 232575_at, | -0.02 | 3.08 | 3.12 | -0.77 | 4.43E-01 | 0.80 |
| ENSG00000275854.1 | RP11-278C7.5 | 236032_at, | 0.02 | 3.70 | 3.64 | 0.77 | 4.45E-01 | 0.80 |
| ENSG00000262668.1 | AJ003147.9 | 207338_s_at,221402_at,217308_at, | 0.01 | 4.86 | 4.84 | 0.77 | 4.45E-01 | 0.80 |
| ENSG00000236886.2 | AC007563.5 | 217424_at,217439_at,203424_s_at, | -0.02 | 4.96 | 5.03 | -0.77 | 4.44E-01 | 0.80 |
| ENSG00000227218.7 | RP11-203J24.8 | 209391_at, | -0.02 | 6.73 | 6.81 | -0.76 | 4.46E-01 | 0.80 |
| ENSG00000246363.2 | RP11-13A1.1 | 1559595_at, | 0.02 | 3.13 | 3.09 | 0.76 | 4.47E-01 | 0.80 |
| ENSG00000234741.7 | GAS5 | 228238_at,227517_s_at, | 0.02 | 7.85 | 7.71 | 0.75 | 4.55E-01 | 0.80 |
| ENSG00000248458.2 | RP4-598P13.1 | 240961_at,235511_at,227979_at, | -0.01 | 2.69 | 2.72 | -0.75 | 4.54E-01 | 0.80 |
| ENSG00000255478.1 | RP11-867O8.5 | 1563498_s_at, | -0.03 | 2.88 | 2.95 | -0.75 | 4.58E-01 | 0.80 |
| ENSG00000277895.1 | RP11-114F3.4 | 1554391_at, | 0.01 | 3.15 | 3.12 | 0.76 | 4.51E-01 | 0.80 |
| ENSG00000257286.1 | RP11-545P7.4 | 221777_at, | 0.01 | 7.04 | 6.98 | 0.75 | 4.57E-01 | 0.80 |
| ENSG00000229537.5 | RP11-5P4.1 | 1562351_at, | 0.01 | 3.18 | 3.15 | 0.76 | 4.50E-01 | 0.80 |
| ENSG00000234810.1 | NA | 216331_at,235487_at,1562033_at,243300_at,1562590_at,  215715_at,239394_at,219230_at,223389_s_at,226667_x_at,  228364_at,242830_at,207006_s_at,218381_s_at,217694_at,229508_at, | 0.01 | 4.61 | 4.58 | 0.75 | 4.56E-01 | 0.80 |
| ENSG00000279516.1 | FAM230C | 211455_at, | 0.02 | 3.78 | 3.74 | 0.75 | 4.57E-01 | 0.80 |
| ENSG00000246283.2 | CTD-2036P10.3 | 1561705_at, | 0.01 | 3.18 | 3.15 | 0.76 | 4.50E-01 | 0.80 |
| ENSG00000260551.1 | PWRN2 | 1568662_at, | 0.01 | 2.43 | 2.41 | 0.75 | 4.56E-01 | 0.80 |
| ENSG00000228141.6 | AC105339.1 | 1563878_a_at, | -0.02 | 4.81 | 4.88 | -0.75 | 4.54E-01 | 0.80 |
| ENSG00000236383.7 | LINC00854 | 1552302_at,1552303_a_at,234580_at, | -0.02 | 5.16 | 5.23 | -0.76 | 4.52E-01 | 0.80 |
| ENSG00000263624.1 | RP11-45M22.3 | 1566885_at, | -0.02 | 4.29 | 4.34 | -0.76 | 4.50E-01 | 0.80 |
| ENSG00000244649.4 | CTD-2377D24.6 | 239332_at, | -0.01 | 4.12 | 4.16 | -0.75 | 4.56E-01 | 0.80 |
| ENSG00000267172.1 | RP11-397A16.3 | 1566586_at, | 0.02 | 4.25 | 4.21 | 0.75 | 4.53E-01 | 0.80 |
| ENSG00000267581.1 | CTC-559E9.4 | 238493_at, | -0.03 | 4.56 | 4.64 | -0.75 | 4.58E-01 | 0.80 |
| ENSG00000230448.5 | LINC00276 | 1569755_at,219250_s_at,242766_at,1552582_at,202557_at,202558_s_at, | -0.03 | 4.71 | 4.79 | -0.76 | 4.52E-01 | 0.80 |
| ENSG00000204685.6 | STARD7-AS1 | 235584_at, | 0.01 | 4.89 | 4.84 | 0.75 | 4.53E-01 | 0.80 |
| ENSG00000225465.8 | RFPL1S | 207934_at, | 0.01 | 2.46 | 2.44 | 0.76 | 4.48E-01 | 0.80 |
| ENSG00000273328.5 | RP11-141M3.6 | 239452_at,1559432_at, | -0.02 | 4.36 | 4.42 | -0.75 | 4.56E-01 | 0.80 |
| ENSG00000228723.6 | SRGAP3-AS2 | 1559333_at, | -0.02 | 3.04 | 3.07 | -0.76 | 4.49E-01 | 0.80 |
| ENSG00000247624.6 | CPEB2-AS1 | 1561471_at, | 0.02 | 3.00 | 2.96 | 0.75 | 4.53E-01 | 0.80 |
| ENSG00000250582.1 | SMAD1-AS2 | 244446_at, | 0.01 | 2.77 | 2.74 | 0.75 | 4.52E-01 | 0.80 |
| ENSG00000254138.1 | RP11-152K4.2 | 232631_at, | -0.02 | 4.48 | 4.53 | -0.76 | 4.50E-01 | 0.80 |
| ENSG00000235531.9 | MSC-AS1 | 217590_s_at,234206_at, | -0.02 | 3.35 | 3.39 | -0.76 | 4.50E-01 | 0.80 |
| ENSG00000235710.4 | RP5-1097F14.3 | 1552942_at, | 0.02 | 4.06 | 4.00 | 0.74 | 4.63E-01 | 0.80 |
| ENSG00000234864.1 | AL022344.5 | 1561212_at, | -0.04 | 3.10 | 3.20 | -0.74 | 4.64E-01 | 0.80 |
| ENSG00000237523.1 | LINC00857 | 1569322_at, | 0.02 | 3.74 | 3.68 | 0.74 | 4.62E-01 | 0.80 |
| ENSG00000233589.1 | RP4-694A7.2 | 214140_at, | -0.03 | 5.72 | 5.84 | -0.74 | 4.61E-01 | 0.80 |
| ENSG00000256661.1 | A2ML1-AS1 | 242870_at,225999_at, | -0.01 | 3.84 | 3.88 | -0.74 | 4.59E-01 | 0.80 |
| ENSG00000246985.7 | SOCS2-AS1 | 1559315_s_at, | -0.04 | 3.22 | 3.30 | -0.74 | 4.62E-01 | 0.80 |
| ENSG00000259442.1 | RP11-752G15.8 | 1559724_at, | -0.02 | 2.79 | 2.83 | -0.74 | 4.60E-01 | 0.80 |
| ENSG00000234494.7 | SP2-AS1 | 211736_at,237795_s_at, | -0.01 | 2.91 | 2.93 | -0.74 | 4.60E-01 | 0.80 |
| ENSG00000247011.2 | RP11-700H6.1 | 1556851_at, | -0.02 | 2.84 | 2.88 | -0.74 | 4.59E-01 | 0.80 |
| ENSG00000265664.1 | RP11-74H8.1 | 221585_at, | 0.02 | 3.71 | 3.66 | 0.73 | 4.64E-01 | 0.80 |
| ENSG00000279220.1 | GPR1-AS | 232799_at, | 0.02 | 2.93 | 2.90 | 0.74 | 4.62E-01 | 0.80 |
| ENSG00000229042.2 | RP1-232N11.2 | 1562052_at, | 0.02 | 3.43 | 3.39 | 0.73 | 4.65E-01 | 0.80 |
| ENSG00000239513.5 | LINC01210 | 1556507_at, | 0.01 | 6.96 | 6.91 | 0.74 | 4.61E-01 | 0.80 |
| ENSG00000251329.1 | RP11-240A16.1 | 1563116_at, | -0.01 | 2.70 | 2.72 | -0.73 | 4.64E-01 | 0.80 |
| ENSG00000260838.3 | RP11-531A24.3 | 1556444_a_at, | 0.03 | 3.62 | 3.56 | 0.74 | 4.65E-01 | 0.80 |
| ENSG00000253389.2 | RP11-930P14.1 | 240363_at, | 0.02 | 4.64 | 4.59 | 0.74 | 4.63E-01 | 0.80 |
| ENSG00000233093.5 | LINC00892 | 1559648_at, | 0.02 | 2.72 | 2.69 | 0.74 | 4.60E-01 | 0.80 |
| ENSG00000249087.6 | ZNF436-AS1 | 229086_at, | -0.02 | 5.83 | 5.90 | -0.73 | 4.66E-01 | 0.80 |
| ENSG00000279801.1 | RP11-75C10.6 | 1560163_at, | -0.01 | 3.45 | 3.48 | -0.73 | 4.66E-01 | 0.80 |
| ENSG00000229558.2 | SACS-AS1 | 1560941_a_at, | -0.01 | 3.08 | 3.11 | -0.73 | 4.66E-01 | 0.80 |
| ENSG00000279295.1 | RP11-683O4.1 | 1559792_at, | -0.02 | 2.59 | 2.62 | -0.73 | 4.67E-01 | 0.80 |
| ENSG00000235054.2 | NA | 1561383_at, | -0.02 | 2.43 | 2.47 | -0.71 | 4.81E-01 | 0.81 |
| ENSG00000237250.3 | RP11-193H5.1 | 215970_at, | -0.01 | 3.20 | 3.23 | -0.70 | 4.86E-01 | 0.81 |
| ENSG00000203325.3 | RP11-277A4.4 | 214185_at, | -0.01 | 5.71 | 5.76 | -0.72 | 4.74E-01 | 0.81 |
| ENSG00000224081.7 | NA | 1561268_at,215103_at,216739_at,1564545_a_at,208468_at,  240104_at,1552338_at, | 0.01 | 3.94 | 3.91 | 0.72 | 4.71E-01 | 0.81 |
| ENSG00000225855.6 | RUSC1-AS1 | 230256_at,1552862_at, | 0.03 | 5.30 | 5.20 | 0.72 | 4.72E-01 | 0.81 |
| ENSG00000233410.1 | LINC01222 | 1561411_at, | -0.02 | 3.69 | 3.75 | -0.72 | 4.72E-01 | 0.81 |
| ENSG00000229240.7 | NA | 1566504_at,231421_at, | -0.02 | 3.19 | 3.23 | -0.70 | 4.84E-01 | 0.81 |
| ENSG00000237491.8 | RP11-206L10.9 | 208954_s_at,1569500_at,1553943_at,218725_at,220806_x_at,  1565558_at,218993_at,236819_at, | 0.01 | 5.27 | 5.24 | 0.72 | 4.76E-01 | 0.81 |
| ENSG00000259994.1 | RP11-305E6.4 | 239863_at, | -0.02 | 3.95 | 4.02 | -0.71 | 4.78E-01 | 0.81 |
| ENSG00000232229.5 | LINC00865 | 232239_at, | -0.02 | 4.25 | 4.32 | -0.70 | 4.84E-01 | 0.81 |
| ENSG00000167355.7 | AC104389.28 | 1570516_s_at,234626_at,234521_at,234775_at, | -0.01 | 3.83 | 3.86 | -0.72 | 4.74E-01 | 0.81 |
| ENSG00000229719.3 | MIR194-2HG | 1556718_s_at,1556717_at, | 0.01 | 5.11 | 5.08 | 0.70 | 4.87E-01 | 0.81 |
| ENSG00000233008.5 | RP11-475O6.1 | 1566947_at, | -0.01 | 2.79 | 2.82 | -0.72 | 4.72E-01 | 0.81 |
| ENSG00000257319.1 | RP11-478B9.1 | 224272_at, | -0.02 | 2.75 | 2.78 | -0.73 | 4.69E-01 | 0.81 |
| ENSG00000256494.1 | RP5-944M2.1 | 1561536_at, | -0.02 | 3.14 | 3.17 | -0.73 | 4.69E-01 | 0.81 |
| ENSG00000223732.2 | RP11-321C24.1 | 226429_at,229078_s_at, | -0.02 | 5.22 | 5.29 | -0.71 | 4.81E-01 | 0.81 |
| ENSG00000227258.5 | SMIM2-AS1 | 220930_s_at,220931_at, | -0.01 | 2.82 | 2.84 | -0.72 | 4.74E-01 | 0.81 |
| ENSG00000258851.1 | RP11-894P9.2 | 230427_s_at,202984_s_at, | -0.02 | 4.28 | 4.34 | -0.71 | 4.77E-01 | 0.81 |
| ENSG00000258038.5 | CTD-2384A14.1 | 241675_s_at,241674_s_at, | -0.03 | 2.72 | 2.79 | -0.71 | 4.77E-01 | 0.81 |
| ENSG00000258377.1 | RP11-649E7.5 | 211061_s_at, | -0.02 | 6.09 | 6.16 | -0.71 | 4.81E-01 | 0.81 |
| ENSG00000259709.1 | CTD-2184D3.7 | 1560094_at,1560095_s_at, | 0.01 | 3.59 | 3.56 | 0.70 | 4.86E-01 | 0.81 |
| ENSG00000260755.1 | RP11-403P17.3 | 204276_at,204277_s_at, | -0.01 | 6.20 | 6.26 | -0.72 | 4.71E-01 | 0.81 |
| ENSG00000260600.1 | RP11-109D24.1 | 1564291_at, | -0.01 | 2.93 | 2.96 | -0.72 | 4.76E-01 | 0.81 |
| ENSG00000260989.1 | LA16c-395F10.2 | 237674_at, | -0.02 | 3.26 | 3.30 | -0.71 | 4.78E-01 | 0.81 |
| ENSG00000260907.1 | AC008088.4 | 224172_at, | -0.02 | 4.18 | 4.25 | -0.70 | 4.85E-01 | 0.81 |
| ENSG00000263069.5 | CTD-2047H16.4 | 225931_s_at,230000_at,233880_at,225929_s_at, | -0.02 | 6.53 | 6.61 | -0.70 | 4.84E-01 | 0.81 |
| ENSG00000175061.17 | LRRC75A-AS1 | 229889_at, | -0.02 | 3.64 | 3.69 | -0.70 | 4.84E-01 | 0.81 |
| ENSG00000229980.4 | TOB1-AS1 | 1561911_at, | -0.01 | 3.06 | 3.09 | -0.71 | 4.79E-01 | 0.81 |
| ENSG00000264860.1 | RP11-143K11.5 | 214597_at,217455_s_at, | -0.02 | 4.03 | 4.09 | -0.70 | 4.84E-01 | 0.81 |
| ENSG00000272884.1 | RP11-104H15.10 | 227271_at, | 0.02 | 5.64 | 5.57 | 0.72 | 4.75E-01 | 0.81 |
| ENSG00000261526.2 | CTB-31O20.2 | 230304_at, | -0.02 | 6.32 | 6.42 | -0.70 | 4.86E-01 | 0.81 |
| ENSG00000234572.1 | AC007880.1 | 1559787_at, | -0.02 | 2.96 | 2.99 | -0.70 | 4.83E-01 | 0.81 |
| ENSG00000235770.5 | LINC00607 | 229479_at, | -0.05 | 4.93 | 5.10 | -0.71 | 4.80E-01 | 0.81 |
| ENSG00000272477.1 | RP11-158G18.1 | 1564776_at, | -0.02 | 3.31 | 3.36 | -0.71 | 4.76E-01 | 0.81 |
| ENSG00000244227.5 | LINC01330 | 1560168_at, | -0.01 | 2.39 | 2.41 | -0.71 | 4.78E-01 | 0.81 |
| ENSG00000229619.3 | MBNL1-AS1 | 232298_at, | 0.02 | 2.73 | 2.70 | 0.72 | 4.73E-01 | 0.81 |
| ENSG00000245748.1 | RP11-367J11.2 | 224622_at, | -0.01 | 7.44 | 7.51 | -0.70 | 4.85E-01 | 0.81 |
| ENSG00000251438.1 | RP11-431M7.2 | 1564160_at, | -0.01 | 2.66 | 2.69 | -0.72 | 4.76E-01 | 0.81 |
| ENSG00000270147.1 | RP11-646I6.6 | 244505_at, | -0.01 | 2.78 | 2.81 | -0.70 | 4.84E-01 | 0.81 |
| ENSG00000272927.1 | RP11-1191J2.5 | 238153_at, | 0.02 | 5.02 | 4.96 | 0.72 | 4.75E-01 | 0.81 |
| ENSG00000272154.4 | AC005754.7 | 216313_at,216355_at,240317_at,221317_x_at,223629_at,  221410_x_at, | 0.02 | 5.14 | 5.09 | 0.70 | 4.87E-01 | 0.81 |
| ENSG00000270021.1 | CTC-203F4.2 | 1558780_a_at, | -0.01 | 3.39 | 3.42 | -0.72 | 4.76E-01 | 0.81 |
| ENSG00000254334.1 | RP11-24P4.1 | 1562078_at, | 0.02 | 4.12 | 4.07 | 0.73 | 4.68E-01 | 0.81 |
| ENSG00000167912.5 | RP11-25K19.1 | 235171_at, | -0.03 | 3.12 | 3.19 | -0.70 | 4.86E-01 | 0.81 |
| ENSG00000279256.1 | RP11-5P15.1 | 1562688_at, | -0.01 | 2.83 | 2.86 | -0.71 | 4.82E-01 | 0.81 |
| ENSG00000269994.1 | RP11-276H19.2 | 229761_at, | 0.02 | 3.89 | 3.84 | 0.72 | 4.73E-01 | 0.81 |
| ENSG00000261731.2 | CTD-2358C21.4 | 238910_at, | 0.02 | 4.55 | 4.49 | 0.70 | 4.88E-01 | 0.81 |
| ENSG00000260391.2 | RP11-71H17.7 | 230596_at, | 0.03 | 3.54 | 3.47 | 0.70 | 4.88E-01 | 0.81 |
| ENSG00000260918.1 | RP11-731J8.2 | 1556904_at, | -0.02 | 2.46 | 2.50 | -0.70 | 4.88E-01 | 0.81 |
| ENSG00000235919.4 | ASH1L-AS1 | 1554057_at, | 0.02 | 6.03 | 5.95 | 0.69 | 4.90E-01 | 0.81 |
| ENSG00000263300.1 | RP5-1029F21.4 | 219794_at, | 0.01 | 3.68 | 3.64 | 0.69 | 4.91E-01 | 0.81 |
| ENSG00000267610.2 | AC007787.2 | 1570167_at, | 0.01 | 3.53 | 3.50 | 0.69 | 4.92E-01 | 0.81 |
| ENSG00000267014.5 | LINC01532 | 1559303_at, | 0.01 | 3.69 | 3.65 | 0.69 | 4.91E-01 | 0.81 |
| ENSG00000244567.1 | AC096772.6 | 235360_at,236063_at, | -0.01 | 3.94 | 3.98 | -0.69 | 4.91E-01 | 0.81 |
| ENSG00000276223.1 | RP4-781B1.5 | 1560620_at, | -0.02 | 4.21 | 4.28 | -0.69 | 4.92E-01 | 0.81 |
| ENSG00000271918.1 | CTD-2287O16.5 | 240595_at,242709_s_at, | -0.01 | 4.20 | 4.23 | -0.69 | 4.91E-01 | 0.81 |
| ENSG00000281796.1 | EHMT1-IT1 | 1559311_at, | -0.01 | 4.14 | 4.18 | -0.69 | 4.91E-01 | 0.81 |
| ENSG00000280278.1 | FLJ30679 | 1553258_at, | 0.02 | 3.66 | 3.61 | 0.69 | 4.93E-01 | 0.81 |
| ENSG00000176593.7 | CTD-2368P22.1 | 1556724_at,1556725_a_at, | -0.01 | 3.50 | 3.52 | -0.69 | 4.93E-01 | 0.81 |
| ENSG00000253716.5 | MINCR | 235428_at, | -0.04 | 4.45 | 4.57 | -0.69 | 4.94E-01 | 0.81 |
| ENSG00000236208.1 | C10orf71-AS1 | 237244_at, | 0.02 | 3.71 | 3.67 | 0.68 | 4.96E-01 | 0.81 |
| ENSG00000280190.1 | RP11-319G9.1 | 234537_at, | 0.01 | 2.87 | 2.84 | 0.69 | 4.95E-01 | 0.81 |
| ENSG00000260372.6 | AQP4-AS1 | 1559276_at,1560751_at,224400_s_at,210067_at,210066_s_at,  226228_at,223737_x_at, | 0.02 | 2.87 | 2.83 | 0.68 | 4.95E-01 | 0.81 |
| ENSG00000204929.11 | AC074391.1 | 1559939_at,217012_at, | -0.01 | 3.34 | 3.38 | -0.68 | 4.95E-01 | 0.81 |
| ENSG00000233117.2 | LINC00702 | 1561776_at, | -0.01 | 2.65 | 2.68 | -0.68 | 4.96E-01 | 0.81 |
| ENSG00000272167.2 | PROX1-AS1 | 1564262_at, | -0.01 | 2.79 | 2.81 | -0.68 | 4.98E-01 | 0.81 |
| ENSG00000255094.1 | RP11-406D1.2 | 1567389_at,1567390_at, | 0.01 | 3.18 | 3.15 | 0.68 | 4.99E-01 | 0.81 |
| ENSG00000272994.1 | RP11-332H14.2 | 236958_at, | 0.02 | 5.10 | 5.03 | 0.68 | 5.01E-01 | 0.81 |
| ENSG00000281398.2 | SNHG4 | 1567681_at,1567679_at,1564906_at,1567682_x_at,1564907_s_at, | -0.01 | 3.38 | 3.40 | -0.68 | 5.01E-01 | 0.81 |
| ENSG00000260640.1 | KB-1000E4.2 | 1560979_a_at, | -0.01 | 2.85 | 2.87 | -0.68 | 5.00E-01 | 0.81 |
| ENSG00000282021.1 | CTD-2336O2.3 | 1569492_at, | -0.01 | 2.81 | 2.84 | -0.68 | 5.01E-01 | 0.81 |
| ENSG00000235387.1 | LINC00961 | 1557371_a_at, | 0.01 | 6.97 | 6.93 | 0.68 | 5.00E-01 | 0.81 |
| ENSG00000248869.5 | RP11-138I17.1 | 243844_at,1569811_at, | -0.01 | 2.66 | 2.68 | -0.67 | 5.02E-01 | 0.81 |
| ENSG00000279324.1 | RP11-93G23.2 | 220871_at, | 0.02 | 4.09 | 4.04 | 0.67 | 5.05E-01 | 0.81 |
| ENSG00000273409.1 | RP11-480C22.1 | 1554224_at, | 0.02 | 2.87 | 2.83 | 0.67 | 5.05E-01 | 0.81 |
| ENSG00000231119.2 | RP4-569M23.2 | 243814_at, | 0.01 | 3.65 | 3.62 | 0.67 | 5.04E-01 | 0.81 |
| ENSG00000260949.1 | KB-1836B5.1 | 1569724_at, | 0.02 | 4.03 | 3.99 | 0.67 | 5.05E-01 | 0.81 |
| ENSG00000281207.1 | SLFNL1-AS1 | 1553315_at,1554074_s_at,220976_s_at,233533_at,220978_at, | 0.01 | 3.73 | 3.71 | 0.66 | 5.09E-01 | 0.82 |
| ENSG00000229846.1 | RP11-141A19.1 | 1559828_at,1553458_at,211058_x_at,220036_s_at,209118_s_at,  1565669_at,213646_x_at,211072_x_at,1553713_a_at,241574_s_at,  220774_at,201221_s_at, | 0.01 | 6.56 | 6.54 | 0.66 | 5.08E-01 | 0.82 |
| ENSG00000255474.1 | RP11-234B24.2 | 1559538_at, | -0.02 | 3.61 | 3.66 | -0.67 | 5.07E-01 | 0.82 |
| ENSG00000228192.7 | RP11-342M1.3 | 209852_x_at, | -0.02 | 6.95 | 7.05 | -0.66 | 5.08E-01 | 0.82 |
| ENSG00000268189.2 | AC005785.2 | 1570133_at, | 0.01 | 4.24 | 4.20 | 0.66 | 5.09E-01 | 0.82 |
| ENSG00000253320.5 | AZIN1-AS1 | 1559029_at,1559030_a_at,1565897_at, | 0.01 | 3.24 | 3.21 | 0.66 | 5.09E-01 | 0.82 |
| ENSG00000236266.1 | RP3-467L1.4 | 1553078_at,222088_s_at, | -0.04 | 3.69 | 3.79 | -0.66 | 5.13E-01 | 0.82 |
| ENSG00000231473.2 | LINC00441 | 1559543_at, | -0.01 | 2.55 | 2.57 | -0.66 | 5.12E-01 | 0.82 |
| ENSG00000259048.1 | CTD-2058B24.2 | 240506_at, | 0.02 | 4.12 | 4.07 | 0.66 | 5.13E-01 | 0.82 |
| ENSG00000279887.1 | RP11-264L1.1 | 236617_at, | -0.03 | 6.12 | 6.26 | -0.66 | 5.13E-01 | 0.82 |
| ENSG00000267659.5 | LINC01482 | 217471_at,241177_at, | 0.01 | 3.42 | 3.40 | 0.66 | 5.12E-01 | 0.82 |
| ENSG00000272667.1 | RP11-395A13.2 | 228839_s_at, | -0.02 | 6.74 | 6.82 | -0.66 | 5.13E-01 | 0.82 |
| ENSG00000254275.6 | LINC00824 | 1555580_at, | -0.02 | 3.10 | 3.13 | -0.66 | 5.12E-01 | 0.82 |
| ENSG00000264443.1 | RP4-594I10.3 | 226272_at,1562966_at,227635_at,228814_at,232044_at,  223802_s_at, | -0.01 | 5.10 | 5.13 | -0.65 | 5.15E-01 | 0.82 |
| ENSG00000265408.1 | RP11-361L15.4 | 213509_x_at,209667_at, | -0.01 | 6.66 | 6.70 | -0.65 | 5.16E-01 | 0.82 |
| ENSG00000149443.7 | C20orf78 | 231134_at, | 0.01 | 3.83 | 3.79 | 0.65 | 5.16E-01 | 0.82 |
| ENSG00000248896.2 | CTD-2135J3.3 | 223907_s_at, | 0.01 | 6.00 | 5.95 | 0.65 | 5.16E-01 | 0.82 |
| ENSG00000243960.1 | RP11-552M11.4 | 201421_s_at, | -0.01 | 7.15 | 7.20 | -0.65 | 5.18E-01 | 0.82 |
| ENSG00000258730.1 | ITPK1-AS1 | 1563524_a_at, | -0.01 | 2.62 | 2.65 | -0.65 | 5.18E-01 | 0.82 |
| ENSG00000268912.1 | CTD-2619J13.17 | 235835_at, | 0.01 | 3.97 | 3.93 | 0.65 | 5.17E-01 | 0.82 |
| ENSG00000235621.8 | LINC00494 | 236854_at, | -0.02 | 3.47 | 3.51 | -0.65 | 5.19E-01 | 0.82 |
| ENSG00000237292.1 | RP11-540K16.1 | 1561045_a_at, | 0.01 | 3.11 | 3.08 | 0.65 | 5.20E-01 | 0.82 |
| ENSG00000279070.2 | RP11-287D1.2 | 1560946_at, | 0.01 | 3.02 | 3.00 | 0.64 | 5.21E-01 | 0.82 |
| ENSG00000270571.2 | RP11-355F16.1 | 243421_at, | 0.01 | 2.52 | 2.49 | 0.64 | 5.21E-01 | 0.82 |
| ENSG00000224957.5 | LINC01266 | 1562588_at, | -0.01 | 2.67 | 2.69 | -0.65 | 5.20E-01 | 0.82 |
| ENSG00000225194.2 | LINC00092 | 1562733_at, | 0.01 | 3.92 | 3.89 | 0.64 | 5.21E-01 | 0.82 |
| ENSG00000257027.1 | RP11-705C15.3 | 1560156_at, | -0.02 | 4.75 | 4.81 | -0.64 | 5.22E-01 | 0.82 |
| ENSG00000235688.2 | AC116614.1 | 241535_at, | 0.06 | 3.19 | 3.07 | 0.64 | 5.22E-01 | 0.82 |
| ENSG00000248510.2 | RP11-145G20.1 | 241479_at, | -0.02 | 2.51 | 2.55 | -0.64 | 5.23E-01 | 0.82 |
| ENSG00000223891.5 | OSER1-AS1 | 1569745_at,1569746_s_at,1558906_a_at, | 0.01 | 4.32 | 4.29 | 0.64 | 5.23E-01 | 0.82 |
| ENSG00000244332.1 | RP11-119K6.6 | 220085_at, | -0.04 | 4.91 | 5.04 | -0.64 | 5.25E-01 | 0.82 |
| ENSG00000254602.1 | AP000662.4 | 1566151_at, | 0.01 | 3.80 | 3.77 | 0.64 | 5.25E-01 | 0.82 |
| ENSG00000235958.5 | UBOX5-AS1 | 204598_at,219016_at, | 0.02 | 5.86 | 5.79 | 0.64 | 5.24E-01 | 0.82 |
| ENSG00000236714.1 | AC005592.1 | 1564083_at, | 0.02 | 3.01 | 2.97 | 0.64 | 5.25E-01 | 0.82 |
| ENSG00000261335.1 | RP11-318A15.2 | 240032_at, | 0.02 | 4.23 | 4.18 | 0.63 | 5.27E-01 | 0.83 |
| ENSG00000279396.1 | AC130469.1 | 234490_at, | 0.02 | 3.36 | 3.32 | 0.63 | 5.27E-01 | 0.83 |
| ENSG00000234474.2 | MIR3663HG | 1561328_at, | -0.01 | 2.55 | 2.57 | -0.63 | 5.28E-01 | 0.83 |
| ENSG00000232656.7 | IDI2-AS1 | 208881_x_at, | 0.02 | 8.08 | 7.99 | 0.63 | 5.30E-01 | 0.83 |
| ENSG00000261373.1 | VPS9D1-AS1 | 1558728_at, | 0.01 | 6.06 | 6.01 | 0.63 | 5.30E-01 | 0.83 |
| ENSG00000265263.1 | RP11-135L13.4 | 203463_s_at, | 0.01 | 5.75 | 5.69 | 0.63 | 5.30E-01 | 0.83 |
| ENSG00000273759.1 | RP4-563E14.1 | 238920_at, | 0.02 | 4.80 | 4.73 | 0.63 | 5.31E-01 | 0.83 |
| ENSG00000254488.1 | RP11-65G9.1 | 1555743_s_at,211614_at, | -0.01 | 5.39 | 5.42 | -0.63 | 5.31E-01 | 0.83 |
| ENSG00000275055.1 | CTC-471J1.11 | 229279_at, | 0.01 | 4.00 | 3.96 | 0.62 | 5.34E-01 | 0.83 |
| ENSG00000258376.2 | RP4-647C14.2 | 1569339_s_at, | -0.01 | 4.24 | 4.28 | -0.62 | 5.35E-01 | 0.83 |
| ENSG00000241469.7 | LINC00635 | 238283_at, | 0.01 | 2.98 | 2.96 | 0.62 | 5.36E-01 | 0.83 |
| ENSG00000263644.1 | RP11-269G24.3 | 224952_at, | 0.03 | 6.37 | 6.25 | 0.62 | 5.38E-01 | 0.83 |
| ENSG00000263812.5 | LINC00908 | 1558849_at, | -0.01 | 3.40 | 3.42 | -0.62 | 5.37E-01 | 0.83 |
| ENSG00000267698.1 | AC002116.7 | 212358_at,228500_at, | -0.01 | 5.40 | 5.44 | -0.62 | 5.38E-01 | 0.83 |
| ENSG00000254480.1 | RP11-23F23.2 | 231028_at, | 0.01 | 2.74 | 2.72 | 0.62 | 5.39E-01 | 0.83 |
| ENSG00000230864.1 | RP5-936J12.1 | 1562573_at,204475_at,1561222_at, | 0.06 | 6.46 | 6.17 | 0.62 | 5.39E-01 | 0.83 |
| ENSG00000280739.2 | EIF1B-AS1 | 1556248_at,1556249_a_at, | -0.01 | 5.12 | 5.17 | -0.61 | 5.44E-01 | 0.84 |
| ENSG00000224074.3 | LINC00691 | 1561059_a_at, | -0.01 | 2.79 | 2.80 | -0.61 | 5.45E-01 | 0.84 |
| ENSG00000224363.2 | RP11-289H16.1 | 232095_at, | -0.02 | 5.63 | 5.69 | -0.60 | 5.47E-01 | 0.84 |
| ENSG00000280161.1 | CTC-205M6.1 | 232690_at, | -0.01 | 5.02 | 5.06 | -0.60 | 5.49E-01 | 0.85 |
| ENSG00000188206.6 | HNRNPU-AS1 | 224820_at,225786_at, | -0.02 | 7.80 | 7.89 | -0.60 | 5.53E-01 | 0.85 |
| ENSG00000272983.1 | RP11-508N22.12 | 1562232_at, | 0.01 | 3.75 | 3.72 | 0.60 | 5.52E-01 | 0.85 |
| ENSG00000258232.2 | RP11-161H23.5 | 211750_x_at, | 0.01 | 11.76 | 11.68 | 0.60 | 5.52E-01 | 0.85 |
| ENSG00000257568.1 | RP11-863H1.1 | 1570298_at, | -0.01 | 2.60 | 2.61 | -0.60 | 5.53E-01 | 0.85 |
| ENSG00000261404.5 | AC009120.4 | 237381_at, | 0.01 | 2.80 | 2.78 | 0.60 | 5.50E-01 | 0.85 |
| ENSG00000227543.4 | SPAG5-AS1 | 1553292_s_at,231541_s_at, | 0.01 | 3.58 | 3.56 | 0.60 | 5.53E-01 | 0.85 |
| ENSG00000226747.6 | AC007966.1 | 1560288_at, | -0.01 | 2.47 | 2.49 | -0.60 | 5.52E-01 | 0.85 |
| ENSG00000253629.1 | KB-1107E3.1 | 211685_s_at, | 0.02 | 5.55 | 5.46 | 0.60 | 5.52E-01 | 0.85 |
| ENSG00000260996.1 | RP13-122B23.8 | 1570601_at,1570600_at, | 0.01 | 3.34 | 3.32 | 0.60 | 5.53E-01 | 0.85 |
| ENSG00000235989.3 | MORC2-AS1 | 230566_at,228449_at, | -0.01 | 5.44 | 5.48 | -0.59 | 5.54E-01 | 0.85 |
| ENSG00000277757.4 | RP11-285G1.15 | 1561482_at, | -0.01 | 2.66 | 2.69 | -0.59 | 5.57E-01 | 0.85 |
| ENSG00000256007.1 | ARAP1-AS1 | 212516_at, | 0.01 | 6.83 | 6.78 | 0.59 | 5.56E-01 | 0.85 |
| ENSG00000247363.2 | RP11-637A17.2 | 1557021_s_at, | -0.01 | 3.22 | 3.25 | -0.59 | 5.58E-01 | 0.85 |
| ENSG00000226438.1 | RP11-420K8.1 | 232412_at, | 0.05 | 3.82 | 3.68 | 0.59 | 5.57E-01 | 0.85 |
| ENSG00000273492.5 | AP000230.1 | 244088_at, | 0.01 | 4.42 | 4.39 | 0.59 | 5.57E-01 | 0.85 |
| ENSG00000256637.6 | RP11-76I14.1 | 1562800_at, | 0.01 | 2.62 | 2.60 | 0.58 | 5.60E-01 | 0.85 |
| ENSG00000181800.5 | CELF2-AS1 | 1560255_at, | -0.02 | 3.53 | 3.57 | -0.58 | 5.63E-01 | 0.85 |
| ENSG00000237361.2 | TUSC8 | 1569436_at, | 0.01 | 3.16 | 3.13 | 0.58 | 5.62E-01 | 0.85 |
| ENSG00000267934.1 | CTB-176F20.3 | 215344_at, | -0.02 | 5.04 | 5.12 | -0.58 | 5.62E-01 | 0.85 |
| ENSG00000235703.5 | LINC00894 | 1562839_at, | -0.01 | 2.67 | 2.69 | -0.58 | 5.63E-01 | 0.85 |
| ENSG00000246528.3 | RP11-159H10.3 | 1558819_at, | -0.02 | 3.04 | 3.08 | -0.58 | 5.64E-01 | 0.86 |
| ENSG00000249835.2 | VCAN-AS1 | 204620_s_at, | -0.02 | 8.55 | 8.68 | -0.58 | 5.65E-01 | 0.86 |
| ENSG00000236528.1 | RP1-125I3.2 | 242123_at, | 0.01 | 6.02 | 5.97 | 0.57 | 5.67E-01 | 0.86 |
| ENSG00000265073.1 | AC010761.6 | 1557170_at, | 0.02 | 7.40 | 7.32 | 0.58 | 5.67E-01 | 0.86 |
| ENSG00000234229.7 | LINC01505 | 1569817_at, | -0.01 | 2.83 | 2.86 | -0.57 | 5.67E-01 | 0.86 |
| ENSG00000231651.1 | DLG3-AS1 | 241839_at, | 0.01 | 4.21 | 4.18 | 0.57 | 5.67E-01 | 0.86 |
| ENSG00000233542.1 | RP11-547D24.1 | 1556401_a_at,1556400_at, | 0.01 | 3.38 | 3.36 | 0.57 | 5.69E-01 | 0.86 |
| ENSG00000234511.8 | C5orf58 | 237956_s_at,244578_at,205269_at,1569417_at, | -0.01 | 3.52 | 3.55 | -0.57 | 5.69E-01 | 0.86 |
| ENSG00000261026.1 | CTD-3247F14.2 | 1562247_at, | -0.01 | 3.32 | 3.36 | -0.57 | 5.70E-01 | 0.86 |
| ENSG00000251533.2 | LINC00605 | 1556505_at, | 0.01 | 3.39 | 3.36 | 0.57 | 5.71E-01 | 0.86 |
| ENSG00000255028.5 | RP11-708B6.2 | 1557181_s_at,1557180_at, | -0.01 | 2.40 | 2.41 | -0.56 | 5.76E-01 | 0.86 |
| ENSG00000275431.1 | CTB-186H2.3 | 1569732_at, | 0.01 | 3.82 | 3.79 | 0.57 | 5.73E-01 | 0.86 |
| ENSG00000280023.1 | LLNLR-276H7.1 | 207779_at, | 0.01 | 2.75 | 2.73 | 0.56 | 5.76E-01 | 0.86 |
| ENSG00000203386.6 | LINC01317 | 208725_at,240449_at,224096_at,1561906_at,224210_s_at,  1564482_at,216628_at,209298_s_at,237326_at,209297_at,  1564244_a_at,1563039_at, | -0.01 | 4.72 | 4.74 | -0.56 | 5.76E-01 | 0.86 |
| ENSG00000260542.1 | RP13-379O24.2 | 1562586_at, | 0.01 | 5.12 | 5.08 | 0.56 | 5.74E-01 | 0.86 |
| ENSG00000231367.5 | AC016995.3 | 1568248_x_at,1568249_at,230180_at,208718_at,207264_at,  204017_at,208719_s_at,208359_s_at, | -0.01 | 7.32 | 7.36 | -0.56 | 5.76E-01 | 0.86 |
| ENSG00000281160.1 | RP11-333B11.1 | 1570171_at, | 0.01 | 3.93 | 3.89 | 0.56 | 5.76E-01 | 0.86 |
| ENSG00000274421.1 | RP11-386J22.3 | 1562948_at, | -0.02 | 3.80 | 3.85 | -0.56 | 5.75E-01 | 0.86 |
| ENSG00000267026.5 | RP11-92C4.3 | 218885_s_at, | 0.04 | 4.18 | 4.07 | 0.56 | 5.74E-01 | 0.86 |
| ENSG00000176984.6 | AP000679.2 | 1564229_at, | 0.01 | 4.13 | 4.10 | 0.55 | 5.80E-01 | 0.87 |
| ENSG00000257747.1 | RP11-362A1.1 | 1562923_at, | 0.01 | 2.92 | 2.89 | 0.55 | 5.80E-01 | 0.87 |
| ENSG00000249786.7 | EAF1-AS1 | 206073_at, | 0.01 | 4.78 | 4.75 | 0.56 | 5.80E-01 | 0.87 |
| ENSG00000227135.1 | GCSAML-AS1 | 232452_at, | -0.01 | 2.55 | 2.57 | -0.55 | 5.87E-01 | 0.87 |
| ENSG00000269609.5 | RPARP-AS1 | 213964_x_at, | -0.01 | 2.62 | 2.64 | -0.55 | 5.84E-01 | 0.87 |
| ENSG00000157306.14 | RP11-66N24.4 | 214341_at,218540_at,207699_at, | -0.01 | 5.39 | 5.41 | -0.55 | 5.85E-01 | 0.87 |
| ENSG00000237899.1 | RP4-739H11.3 | 1560081_at, | -0.01 | 5.64 | 5.70 | -0.55 | 5.84E-01 | 0.87 |
| ENSG00000234327.7 | AC012146.7 | 236074_at, | -0.01 | 2.61 | 2.63 | -0.54 | 5.87E-01 | 0.87 |
| ENSG00000265943.1 | RP11-739L10.1 | 1561149_at, | -0.01 | 3.11 | 3.13 | -0.55 | 5.85E-01 | 0.87 |
| ENSG00000229839.6 | AC018462.2 | 239833_at, | -0.02 | 4.75 | 4.82 | -0.55 | 5.86E-01 | 0.87 |
| ENSG00000225490.1 | NA | 241803_s_at, | -0.02 | 2.96 | 3.01 | -0.55 | 5.85E-01 | 0.87 |
| ENSG00000260633.1 | RP11-375I20.6 | 234592_at, | 0.01 | 3.22 | 3.20 | 0.55 | 5.82E-01 | 0.87 |
| ENSG00000261051.1 | RP11-274H2.5 | 232451_at, | -0.02 | 2.97 | 3.00 | -0.55 | 5.83E-01 | 0.87 |
| ENSG00000250490.1 | FLJ33360 | 1563969_at, | -0.02 | 3.69 | 3.74 | -0.55 | 5.84E-01 | 0.87 |
| ENSG00000233937.6 | CTC-338M12.4 | 221897_at, | -0.02 | 4.08 | 4.13 | -0.54 | 5.87E-01 | 0.87 |
| ENSG00000271833.1 | RP11-356B19.11 | 238227_at, | -0.02 | 4.20 | 4.25 | -0.55 | 5.86E-01 | 0.87 |
| ENSG00000255248.6 | RP11-166D19.1 | 232113_at,225381_at,233364_s_at, | -0.03 | 5.59 | 5.69 | -0.54 | 5.88E-01 | 0.87 |
| ENSG00000264112.1 | RP11-159D12.2 | 226419_s_at,239384_at, | 0.02 | 5.46 | 5.40 | 0.54 | 5.89E-01 | 0.87 |
| ENSG00000197099.8 | RP11-573D15.8 | 206226_at,210929_s_at,204551_s_at, | 0.01 | 3.70 | 3.67 | 0.54 | 5.89E-01 | 0.87 |
| ENSG00000233427.1 | RP1-212P9.3 | 1559110_at,209322_s_at,40149_at", | 0.01 | 5.83 | 5.81 | 0.53 | 5.94E-01 | 0.87 |
| ENSG00000270380.1 | RP11-470L19.5 | 229124_at, | 0.01 | 4.57 | 4.54 | 0.54 | 5.92E-01 | 0.87 |
| ENSG00000227253.3 | RP11-166N17.1 | 225417_at, | -0.01 | 7.39 | 7.46 | -0.54 | 5.92E-01 | 0.87 |
| ENSG00000279814.1 | RP11-399K21.12 | 1559117_at, | -0.02 | 4.17 | 4.22 | -0.53 | 5.96E-01 | 0.87 |
| ENSG00000255929.5 | RP11-867G2.8 | 209892_at,209893_s_at, | -0.02 | 4.61 | 4.67 | -0.54 | 5.92E-01 | 0.87 |
| ENSG00000246273.6 | SBF2-AS1 | 1566830_at,1566831_at, | 0.01 | 3.26 | 3.23 | 0.53 | 5.95E-01 | 0.87 |
| ENSG00000225886.3 | RP11-288L9.4 | 244503_at,203736_s_at,237661_at, | 0.02 | 3.27 | 3.22 | 0.53 | 5.94E-01 | 0.87 |
| ENSG00000226891.6 | NA | 237629_at, | -0.01 | 3.43 | 3.46 | -0.53 | 5.96E-01 | 0.87 |
| ENSG00000233208.5 | LINC00642 | 1557656_at, | -0.01 | 5.58 | 5.61 | -0.54 | 5.93E-01 | 0.87 |
| ENSG00000279633.1 | RP11-442G21.2 | 1557882_at,1557883_a_at, | -0.01 | 2.54 | 2.55 | -0.53 | 5.95E-01 | 0.87 |
| ENSG00000177699.4 | RP11-16K12.1 | 215688_at, | 0.01 | 3.77 | 3.74 | 0.54 | 5.92E-01 | 0.87 |
| ENSG00000261173.1 | RP11-169E6.1 | 243359_at, | 0.01 | 2.95 | 2.93 | 0.53 | 5.94E-01 | 0.87 |
| ENSG00000272770.1 | RP11-74E22.5 | 200813_s_at, | -0.01 | 5.81 | 5.86 | -0.53 | 5.94E-01 | 0.87 |
| ENSG00000204282.4 | TNRC6C-AS1 | 239062_at, | -0.03 | 3.58 | 3.64 | -0.54 | 5.93E-01 | 0.87 |
| ENSG00000275139.1 | RP1-101D8.1 | 1566807_a_at,1566809_a_at, | -0.01 | 3.47 | 3.49 | -0.53 | 5.98E-01 | 0.87 |
| ENSG00000223797.5 | ENTPD3-AS1 | 1555118_at,1556558_s_at,206191_at, | -0.02 | 3.38 | 3.42 | -0.53 | 5.98E-01 | 0.87 |
| ENSG00000254664.1 | CTD-2560E9.3 | 220571_at,229687_s_at,229688_at,1559567_at, | 0.01 | 4.03 | 4.01 | 0.53 | 5.99E-01 | 0.87 |
| ENSG00000248550.3 | OTX2-AS1 | 1561319_at, | -0.01 | 2.71 | 2.73 | -0.53 | 6.00E-01 | 0.87 |
| ENSG00000213373.7 | LINC00671 | 1556737_at,1570189_at, | 0.01 | 3.36 | 3.34 | 0.53 | 6.00E-01 | 0.87 |
| ENSG00000231826.5 | AC016735.2 | 216004_s_at, | 0.01 | 3.27 | 3.25 | 0.53 | 6.00E-01 | 0.87 |
| ENSG00000270189.1 | RP11-258C19.7 | 228539_at,229869_at, | -0.02 | 4.89 | 4.95 | -0.53 | 5.99E-01 | 0.87 |
| ENSG00000170983.3 | LINC00208 | 1552955_at, | 0.01 | 3.98 | 3.95 | 0.52 | 6.01E-01 | 0.87 |
| ENSG00000254317.1 | RP11-473O4.5 | 217916_s_at,228987_at, | -0.01 | 6.55 | 6.61 | -0.52 | 6.02E-01 | 0.87 |
| ENSG00000272940.1 | CTA-384D8.33 | 240954_at, | 0.01 | 4.11 | 4.08 | 0.52 | 6.03E-01 | 0.87 |
| ENSG00000230415.1 | RP5-902P8.10 | 233881_s_at, | -0.01 | 5.01 | 5.04 | -0.52 | 6.03E-01 | 0.87 |
| ENSG00000278997.1 | RP11-131M11.2 | 240756_at, | -0.01 | 3.11 | 3.13 | -0.52 | 6.04E-01 | 0.87 |
| ENSG00000243415.2 | RP11-274H2.2 | 220787_at, | -0.01 | 2.51 | 2.52 | -0.52 | 6.05E-01 | 0.87 |
| ENSG00000236508.1 | ATP13A5-AS1 | 1561332_at, | 0.01 | 4.08 | 4.04 | 0.52 | 6.05E-01 | 0.87 |
| ENSG00000253972.5 | RP11-4K16.2 | 224650_at, | 0.02 | 8.81 | 8.67 | 0.52 | 6.05E-01 | 0.87 |
| ENSG00000262873.1 | CTD-2561B21.11 | 238217_at, | -0.01 | 3.21 | 3.23 | -0.52 | 6.07E-01 | 0.87 |
| ENSG00000269836.1 | CTD-3032J10.4 | 242742_at, | 0.01 | 5.81 | 5.76 | 0.52 | 6.07E-01 | 0.87 |
| ENSG00000232184.1 | RP11-370K11.1 | 240568_at, | 0.02 | 3.69 | 3.64 | 0.51 | 6.08E-01 | 0.88 |
| ENSG00000226067.6 | LINC00623 | 229429_x_at,242215_at,219022_at, | 0.01 | 6.30 | 6.24 | 0.51 | 6.09E-01 | 0.88 |
| ENSG00000235097.1 | LINC00330 | 1564263_at, | 0.01 | 3.84 | 3.81 | 0.51 | 6.09E-01 | 0.88 |
| ENSG00000260887.2 | CASC22 | 1556954_at, | 0.01 | 2.89 | 2.87 | 0.51 | 6.09E-01 | 0.88 |
| ENSG00000205791.3 | LOH12CR2 | 236311_at, | 0.02 | 4.22 | 4.17 | 0.51 | 6.11E-01 | 0.88 |
| ENSG00000215256.3 | DHRS4-AS1 | 1569672_at,224153_s_at, | 0.01 | 3.10 | 3.07 | 0.51 | 6.12E-01 | 0.88 |
| ENSG00000262877.4 | RP11-1055B8.4 | 236553_at, | 0.02 | 4.10 | 4.05 | 0.51 | 6.11E-01 | 0.88 |
| ENSG00000271714.1 | CTD-2377O17.1 | 1562920_at, | -0.01 | 3.50 | 3.53 | -0.51 | 6.12E-01 | 0.88 |
| ENSG00000230728.1 | RP11-240D10.4 | 1569969_a_at, | -0.01 | 2.40 | 2.41 | -0.51 | 6.14E-01 | 0.88 |
| ENSG00000267097.1 | SLC14A2-AS1 | 237612_at, | 0.01 | 3.76 | 3.73 | 0.51 | 6.13E-01 | 0.88 |
| ENSG00000233005.1 | AC067959.1 | 228291_s_at,231445_at,212965_at,200676_s_at,1555470_a_at,  240115_at,212271_at,209299_x_at,228788_at,1562962_at,  1555792_a_at,206064_s_at,1552263_at,200684_s_at,242106_at, | -0.01 | 5.25 | 5.27 | -0.51 | 6.14E-01 | 0.88 |
| ENSG00000205054.6 | LINC01121 | 228909_at,226995_at,1569032_at,1555546_a_at,209578_s_at,  1552819_at,207999_s_at,212421_at,217118_s_at,1560511_at, | 0.01 | 5.80 | 5.78 | 0.51 | 6.14E-01 | 0.88 |
| ENSG00000278133.1 | RP11-196G11.5 | 228710_at, | -0.02 | 6.68 | 6.75 | -0.50 | 6.17E-01 | 0.88 |
| ENSG00000254187.1 | CTB-78F1.1 | 1568878_at, | 0.01 | 3.30 | 3.27 | 0.50 | 6.17E-01 | 0.88 |
| ENSG00000253532.1 | CTD-2340D6.1 | 1570196_at, | -0.01 | 2.83 | 2.86 | -0.50 | 6.16E-01 | 0.88 |
| ENSG00000264985.1 | RP11-449L23.2 | 1560410_at, | -0.01 | 3.86 | 3.89 | -0.50 | 6.18E-01 | 0.88 |
| ENSG00000230876.6 | LINC00486 | 227547_at,201147_s_at,201150_s_at, | -0.02 | 7.42 | 7.53 | -0.50 | 6.19E-01 | 0.88 |
| ENSG00000251175.5 | RP11-45L9.1 | 220063_at, | -0.01 | 5.25 | 5.30 | -0.50 | 6.20E-01 | 0.88 |
| ENSG00000251364.6 | CTD-2516F10.2 | 1557177_at, | 0.01 | 2.94 | 2.92 | 0.49 | 6.23E-01 | 0.88 |
| ENSG00000255794.6 | RMST | 222325_at,229782_at,1562633_at, | -0.03 | 3.35 | 3.42 | -0.49 | 6.24E-01 | 0.88 |
| ENSG00000221819.5 | GAS8-AS1 | 207615_s_at, | 0.01 | 3.25 | 3.23 | 0.49 | 6.24E-01 | 0.88 |
| ENSG00000260913.1 | LINC01254 | 1561083_at, | 0.01 | 3.00 | 2.98 | 0.49 | 6.25E-01 | 0.88 |
| ENSG00000269037.1 | CTC-523E23.6 | 1556914_at, | -0.01 | 4.64 | 4.68 | -0.49 | 6.25E-01 | 0.88 |
| ENSG00000272156.1 | RP11-477N3.1 | 237627_at, | -0.01 | 3.64 | 3.66 | -0.49 | 6.25E-01 | 0.88 |
| ENSG00000226913.1 | BSN-AS2 | 1564131_a_at,240644_at, | 0.01 | 4.54 | 4.52 | 0.50 | 6.21E-01 | 0.88 |
| ENSG00000260303.1 | RP11-203B7.2 | 1562269_at, | -0.01 | 3.46 | 3.49 | -0.49 | 6.23E-01 | 0.88 |
| ENSG00000253656.1 | KB-1568E2.1 | 1555344_at, | -0.01 | 2.65 | 2.67 | -0.49 | 6.25E-01 | 0.88 |
| ENSG00000279935.1 | RP11-447M12.3 | 1561990_at, | 0.01 | 3.92 | 3.90 | 0.49 | 6.22E-01 | 0.88 |
| ENSG00000261212.1 | RP3-507I15.2 | 1560996_at, | 0.01 | 2.54 | 2.52 | 0.50 | 6.21E-01 | 0.88 |
| ENSG00000269887.1 | RP11-477H21.2 | 239210_at, | 0.03 | 3.98 | 3.89 | 0.48 | 6.33E-01 | 0.88 |
| ENSG00000232993.1 | RP11-334A14.5 | 226522_at,224014_at,239954_at, | -0.01 | 4.57 | 4.60 | -0.48 | 6.33E-01 | 0.88 |
| ENSG00000232170.5 | LINC00708 | 237529_at, | 0.01 | 3.04 | 3.02 | 0.48 | 6.33E-01 | 0.88 |
| ENSG00000237413.5 | MGC27382 | 238403_at,225061_at,1563897_at,237208_at,  227697_at,206360_s_at, | 0.01 | 4.96 | 4.93 | 0.48 | 6.32E-01 | 0.88 |
| ENSG00000279462.1 | RP11-604N13.1 | 1570281_at, | 0.01 | 4.16 | 4.13 | 0.48 | 6.32E-01 | 0.88 |
| ENSG00000258573.5 | RP11-14J7.6 | 1562581_at, | -0.01 | 3.99 | 4.02 | -0.48 | 6.31E-01 | 0.88 |
| ENSG00000267048.1 | RP11-566K11.7 | 220715_at, | 0.01 | 3.61 | 3.59 | 0.48 | 6.32E-01 | 0.88 |
| ENSG00000266958.1 | AC006126.3 | 1552889_a_at, | 0.01 | 7.18 | 7.15 | 0.49 | 6.28E-01 | 0.88 |
| ENSG00000281162.2 | LINC01127 | 1560679_at, | 0.01 | 3.00 | 2.97 | 0.48 | 6.31E-01 | 0.88 |
| ENSG00000224577.1 | LINC01117 | 240940_at, | -0.01 | 4.39 | 4.42 | -0.48 | 6.31E-01 | 0.88 |
| ENSG00000234423.1 | LINC01250 | 227118_s_at,204598_at, | 0.01 | 4.17 | 4.14 | 0.48 | 6.30E-01 | 0.88 |
| ENSG00000260066.1 | CTD-2587M23.1 | 1561261_at, | 0.01 | 3.01 | 2.98 | 0.48 | 6.33E-01 | 0.88 |
| ENSG00000253792.1 | CTC-436K13.5 | 244210_at, | 0.01 | 3.28 | 3.26 | 0.48 | 6.30E-01 | 0.88 |
| ENSG00000247516.4 | MIR4458HG | 236591_at, | 0.02 | 3.59 | 3.54 | 0.48 | 6.29E-01 | 0.88 |
| ENSG00000253733.3 | LZTS1-AS1 | 1559880_at, | 0.01 | 4.06 | 4.03 | 0.48 | 6.31E-01 | 0.88 |
| ENSG00000259905.5 | PWRN1 | 1556263_s_at, | -0.01 | 2.62 | 2.63 | -0.48 | 6.36E-01 | 0.88 |
| ENSG00000259666.2 | LINGO1-AS1 | 1562748_at, | -0.01 | 3.35 | 3.37 | -0.48 | 6.35E-01 | 0.88 |
| ENSG00000259992.1 | RP11-77K12.8 | 219384_s_at,209046_s_at, | -0.01 | 7.17 | 7.23 | -0.47 | 6.36E-01 | 0.88 |
| ENSG00000267160.1 | RP11-1072C15.4 | 1562484_at, | 0.02 | 2.74 | 2.71 | 0.47 | 6.37E-01 | 0.88 |
| ENSG00000178803.10 | ADORA2A-AS1 | 239513_at, | -0.01 | 3.51 | 3.53 | -0.47 | 6.38E-01 | 0.88 |
| ENSG00000249502.1 | AC006160.5 | 217933_s_at, | -0.02 | 8.65 | 8.75 | -0.48 | 6.35E-01 | 0.88 |
| ENSG00000247081.7 | BAALC-AS1 | 222780_s_at,218899_s_at, | 0.03 | 3.53 | 3.46 | 0.47 | 6.37E-01 | 0.88 |
| ENSG00000224216.1 | RP13-228J13.1 | 1560797_s_at, | 0.01 | 2.74 | 2.72 | 0.47 | 6.37E-01 | 0.88 |
| ENSG00000224592.5 | RP5-884C9.2 | 234485_at,231864_at, | 0.01 | 5.80 | 5.76 | 0.47 | 6.39E-01 | 0.89 |
| ENSG00000254409.1 | RP11-613D13.4 | 227099_s_at, | -0.02 | 8.40 | 8.49 | -0.47 | 6.39E-01 | 0.89 |
| ENSG00000260108.1 | RP11-140H17.1 | 227382_at, | 0.01 | 3.90 | 3.86 | 0.47 | 6.41E-01 | 0.89 |
| ENSG00000234899.9 | SOX9-AS1 | 202935_s_at,1564466_at, | 0.03 | 6.64 | 6.52 | 0.47 | 6.41E-01 | 0.89 |
| ENSG00000267757.4 | EML2-AS1 | 1569086_at, | 0.01 | 3.76 | 3.73 | 0.47 | 6.41E-01 | 0.89 |
| ENSG00000268833.1 | AC011513.4 | 203757_s_at,211657_at, | 0.04 | 7.02 | 6.81 | 0.47 | 6.40E-01 | 0.89 |
| ENSG00000236445.4 | LINC00608 | 239506_s_at, | -0.01 | 2.69 | 2.71 | -0.47 | 6.42E-01 | 0.89 |
| ENSG00000248079.2 | DPH6-AS1 | 233262_at, | -0.02 | 2.74 | 2.77 | -0.46 | 6.43E-01 | 0.89 |
| ENSG00000262222.1 | RP11-876N24.4 | 238376_at, | 0.01 | 2.80 | 2.78 | 0.46 | 6.45E-01 | 0.89 |
| ENSG00000234787.7 | LINC00458 | 1569287_at, | -0.01 | 2.72 | 2.74 | -0.46 | 6.45E-01 | 0.89 |
| ENSG00000280376.1 | RP11-93O14.3 | 1561146_at, | -0.01 | 2.88 | 2.90 | -0.46 | 6.46E-01 | 0.89 |
| ENSG00000224184.5 | MIR3681HG | 237880_at,233590_at,216422_at,243462_s_at,1569794_at, | -0.01 | 3.54 | 3.56 | -0.46 | 6.47E-01 | 0.89 |
| ENSG00000236065.2 | RP1-117O3.2 | 215480_at,1569407_at, | 0.01 | 2.98 | 2.97 | 0.46 | 6.50E-01 | 0.89 |
| ENSG00000177112.7 | MRVI1-AS1 | 226047_at, | 0.01 | 6.52 | 6.46 | 0.46 | 6.49E-01 | 0.89 |
| ENSG00000260616.6 | RP11-327F22.4 | 213295_at,60084_at,214272_at, | -0.01 | 4.61 | 4.64 | -0.46 | 6.48E-01 | 0.89 |
| ENSG00000231595.1 | AC005224.2 | 1569685_at, | 0.01 | 3.54 | 3.51 | 0.46 | 6.49E-01 | 0.89 |
| ENSG00000233912.1 | AC026202.3 | 217852_s_at,222442_s_at, | -0.01 | 8.00 | 8.07 | -0.46 | 6.48E-01 | 0.89 |
| ENSG00000255052.4 | FAM66D | 222184_at, | 0.01 | 4.26 | 4.22 | 0.46 | 6.49E-01 | 0.89 |
| ENSG00000232677.6 | LINC00665 | 242462_at,1557046_x_at,1557044_at, | 0.01 | 3.80 | 3.78 | 0.45 | 6.51E-01 | 0.89 |
| ENSG00000237101.1 | RP11-365O16.6 | 1561530_at, | -0.01 | 4.73 | 4.77 | -0.45 | 6.52E-01 | 0.89 |
| ENSG00000260517.2 | RP11-426C22.5 | 1559297_at,237556_at,230952_at, | 0.01 | 2.92 | 2.91 | 0.45 | 6.54E-01 | 0.89 |
| ENSG00000266049.1 | RP11-703M24.5 | 241621_at,241620_at, | 0.01 | 3.91 | 3.88 | 0.45 | 6.55E-01 | 0.89 |
| ENSG00000233729.1 | AC016909.1 | 1561460_at, | -0.01 | 3.48 | 3.50 | -0.45 | 6.56E-01 | 0.89 |
| ENSG00000228509.5 | AC006460.2 | 209272_at, | -0.01 | 7.39 | 7.46 | -0.45 | 6.56E-01 | 0.89 |
| ENSG00000253311.1 | AC011343.1 | 1563484_at, | -0.01 | 3.77 | 3.79 | -0.45 | 6.55E-01 | 0.89 |
| ENSG00000254980.1 | RP11-794P6.6 | 1564729_at, | 0.01 | 4.06 | 4.03 | 0.45 | 6.57E-01 | 0.89 |
| ENSG00000255390.1 | RP11-732A19.5 | 218892_at, | 0.01 | 4.30 | 4.27 | 0.44 | 6.58E-01 | 0.90 |
| ENSG00000228496.1 | AC106875.1 | 234250_at, | -0.01 | 3.17 | 3.19 | -0.44 | 6.59E-01 | 0.90 |
| ENSG00000246130.1 | RP11-875O11.2 | 234141_s_at, | -0.01 | 3.95 | 3.97 | -0.44 | 6.59E-01 | 0.90 |
| ENSG00000228843.2 | RP11-112J3.15 | 203169_at, | 0.01 | 6.32 | 6.28 | 0.44 | 6.62E-01 | 0.90 |
| ENSG00000261683.1 | LINC00838 | 1561984_at, | 0.01 | 3.17 | 3.15 | 0.44 | 6.64E-01 | 0.90 |
| ENSG00000261008.6 | LINC01572 | 241637_at, | 0.01 | 5.54 | 5.51 | 0.44 | 6.64E-01 | 0.90 |
| ENSG00000259439.2 | RP11-89K21.1 | 244744_at,237039_at,1555393_s_at,229040_at,1555627_s_at, | 0.01 | 4.25 | 4.22 | 0.43 | 6.66E-01 | 0.90 |
| ENSG00000253618.1 | GRPEL2-AS1 | 226881_at, | 0.01 | 5.66 | 5.61 | 0.43 | 6.65E-01 | 0.90 |
| ENSG00000241577.1 | RP11-523O18.7 | 1554331_a_at, | 0.01 | 3.62 | 3.60 | 0.43 | 6.67E-01 | 0.90 |
| ENSG00000267194.1 | RP1-193H18.2 | 229569_at, | 0.02 | 4.69 | 4.62 | 0.43 | 6.67E-01 | 0.90 |
| ENSG00000196366.3 | C9orf163 | 1553642_at, | -0.01 | 5.53 | 5.55 | -0.43 | 6.67E-01 | 0.90 |
| ENSG00000258661.1 | RP11-964E11.2 | 1554214_at, | 0.01 | 2.70 | 2.68 | 0.43 | 6.69E-01 | 0.90 |
| ENSG00000279732.1 | RP11-319G9.4 | 234838_at, | -0.02 | 4.19 | 4.23 | -0.43 | 6.68E-01 | 0.90 |
| ENSG00000260808.1 | CTD-2007L18.5 | 222467_s_at,217928_s_at, | -0.01 | 7.19 | 7.23 | -0.43 | 6.72E-01 | 0.91 |
| ENSG00000226715.1 | NA | 230364_at, | -0.03 | 5.19 | 5.30 | -0.42 | 6.72E-01 | 0.91 |
| ENSG00000236948.1 | RP11-154H17.1 | 223830_s_at,223599_at,210705_s_at, | -0.01 | 4.05 | 4.08 | -0.42 | 6.74E-01 | 0.91 |
| ENSG00000269938.1 | RP11-214K3.20 | 1556625_a_at,1556624_at, | -0.01 | 2.46 | 2.47 | -0.42 | 6.74E-01 | 0.91 |
| ENSG00000260922.1 | RP11-538I12.3 | 230040_at, | -0.02 | 4.53 | 4.58 | -0.42 | 6.73E-01 | 0.91 |
| ENSG00000230402.2 | LINC01349 | 238756_at, | -0.02 | 6.14 | 6.23 | -0.42 | 6.76E-01 | 0.91 |
| ENSG00000237552.1 | RP11-415A20.1 | 230546_at,239810_at, | 0.01 | 4.93 | 4.90 | 0.42 | 6.77E-01 | 0.91 |
| ENSG00000231993.1 | EP300-AS1 | 213579_s_at,1562921_at, | 0.03 | 4.44 | 4.34 | 0.42 | 6.77E-01 | 0.91 |
| ENSG00000231312.6 | AC007246.3 | 235630_at,206947_at,204593_s_at,204594_s_at,  200779_at,221516_s_at,1566973_at, | 0.00 | 6.63 | 6.61 | 0.42 | 6.79E-01 | 0.91 |
| ENSG00000228630.5 | HOTAIR | 239153_at, | -0.04 | 3.83 | 3.93 | -0.41 | 6.80E-01 | 0.91 |
| ENSG00000272123.1 | CTD-2366F13.2 | 237728_at, | -0.01 | 3.57 | 3.59 | -0.41 | 6.81E-01 | 0.91 |
| ENSG00000233147.1 | NA | 1567285_at,1567286_at,227106_at,204574_s_at, | 0.01 | 4.61 | 4.59 | 0.41 | 6.81E-01 | 0.91 |
| ENSG00000274265.4 | CH17-189H20.1 | 1558588_at,209700_x_at, | -0.01 | 3.26 | 3.28 | -0.40 | 6.88E-01 | 0.91 |
| ENSG00000235358.1 | RP11-399E6.1 | 211369_at, | -0.01 | 3.19 | 3.21 | -0.41 | 6.86E-01 | 0.91 |
| ENSG00000262482.1 | LA16c-321D4.2 | 223950_s_at, | 0.01 | 6.79 | 6.76 | 0.41 | 6.83E-01 | 0.91 |
| ENSG00000246777.1 | RP11-61A14.4 | 217443_at, | -0.01 | 2.65 | 2.66 | -0.40 | 6.87E-01 | 0.91 |
| ENSG00000266120.1 | RP11-354P11.2 | 202179_at, | -0.02 | 5.16 | 5.22 | -0.41 | 6.84E-01 | 0.91 |
| ENSG00000260025.1 | RP11-490M8.1 | 236656_s_at, | 0.02 | 5.71 | 5.62 | 0.41 | 6.86E-01 | 0.91 |
| ENSG00000236049.1 | AC104777.2 | 1562463_at, | 0.01 | 3.71 | 3.70 | 0.40 | 6.88E-01 | 0.91 |
| ENSG00000224063.5 | AC007319.1 | 214378_at,213258_at, | -0.02 | 3.88 | 3.93 | -0.40 | 6.87E-01 | 0.91 |
| ENSG00000226994.7 | AC012593.1 | 234312_s_at,201415_at,226470_at,215795_at,1557527_at,  210365_at,232707_at, | 0.01 | 5.40 | 5.38 | 0.41 | 6.85E-01 | 0.91 |
| ENSG00000244625.5 | MIATNB | 232340_at, | -0.01 | 4.43 | 4.46 | -0.41 | 6.84E-01 | 0.91 |
| ENSG00000231459.1 | LINC00032 | 1559291_at, | 0.01 | 3.92 | 3.89 | 0.41 | 6.86E-01 | 0.91 |
| ENSG00000234405.1 | LL0XNC01-250H12.3 | 239284_at, | -0.01 | 4.38 | 4.41 | -0.41 | 6.85E-01 | 0.91 |
| ENSG00000254814.1 | RP11-535A19.1 | 224327_s_at, | 0.02 | 6.52 | 6.45 | 0.40 | 6.88E-01 | 0.91 |
| ENSG00000258168.5 | RP11-588H23.3 | 1560105_at,244343_at, | -0.01 | 3.61 | 3.63 | -0.40 | 6.91E-01 | 0.92 |
| ENSG00000245149.3 | RNF139-AS1 | 227745_at, | -0.02 | 4.44 | 4.50 | -0.40 | 6.91E-01 | 0.92 |
| ENSG00000225028.1 | RP11-330M19.1 | 1561440_at, | 0.01 | 3.63 | 3.60 | 0.39 | 6.98E-01 | 0.92 |
| ENSG00000236810.5 | TCEB3-AS1 | 213604_at, | -0.01 | 7.16 | 7.20 | -0.39 | 6.99E-01 | 0.92 |
| ENSG00000232995.7 | RGS5 | 230678_at, | -0.01 | 3.52 | 3.56 | -0.39 | 6.95E-01 | 0.92 |
| ENSG00000228463.8 | NA | 212504_at,215698_at,226367_at,235151_at,230226_s_at,  206889_at,1554643_at,206107_at, | -0.01 | 4.55 | 4.57 | -0.39 | 6.97E-01 | 0.92 |
| ENSG00000255580.1 | AP000462.2 | 220666_at, | 0.01 | 2.60 | 2.58 | 0.38 | 7.04E-01 | 0.92 |
| ENSG00000256751.5 | PLBD1-AS1 | 206312_at, | 0.01 | 2.99 | 2.98 | 0.39 | 6.97E-01 | 0.92 |
| ENSG00000272368.2 | RP4-605O3.4 | 1556783_a_at,239491_at, | -0.01 | 2.76 | 2.77 | -0.39 | 7.00E-01 | 0.92 |
| ENSG00000249628.2 | LINC00942 | 1558308_at, | 0.01 | 2.84 | 2.82 | 0.39 | 6.98E-01 | 0.92 |
| ENSG00000258538.5 | RP11-753D20.3 | 1560325_at, | -0.01 | 2.52 | 2.53 | -0.38 | 7.05E-01 | 0.92 |
| ENSG00000270240.2 | AC015849.2 | 1405_i_at", | 0.03 | 6.80 | 6.66 | 0.39 | 6.99E-01 | 0.92 |
| ENSG00000265678.1 | RP11-1376P16.2 | 214818_at, | -0.01 | 5.19 | 5.21 | -0.40 | 6.93E-01 | 0.92 |
| ENSG00000267583.5 | RP11-322E11.5 | 1559716_at, | -0.01 | 4.08 | 4.12 | -0.39 | 7.00E-01 | 0.92 |
| ENSG00000266283.1 | RP11-627G18.1 | 210002_at, | 0.02 | 5.63 | 5.54 | 0.38 | 7.04E-01 | 0.92 |
| ENSG00000269749.1 | AC005614.5 | 215570_s_at,238907_at, | 0.01 | 2.67 | 2.66 | 0.38 | 7.03E-01 | 0.92 |
| ENSG00000279948.1 | CTD-2233K9.1 | 1565882_at, | -0.01 | 3.64 | 3.66 | -0.38 | 7.02E-01 | 0.92 |
| ENSG00000232046.6 | AC007392.3 | 233249_at, | 0.01 | 3.98 | 3.95 | 0.38 | 7.03E-01 | 0.92 |
| ENSG00000231453.1 | LINC01305 | 1557570_a_at, | -0.02 | 3.04 | 3.08 | -0.39 | 6.96E-01 | 0.92 |
| ENSG00000260977.1 | RP11-333I13.1 | 230227_at, | 0.01 | 3.02 | 3.00 | 0.38 | 7.05E-01 | 0.92 |
| ENSG00000234663.5 | AC104820.2 | 1566462_at,1566463_at, | -0.01 | 2.70 | 2.71 | -0.39 | 6.98E-01 | 0.92 |
| ENSG00000223536.5 | AC008069.1 | 1561965_at, | 0.01 | 2.71 | 2.69 | 0.38 | 7.04E-01 | 0.92 |
| ENSG00000244459.2 | RP11-1398P2.1 | 1558890_at, | -0.01 | 4.84 | 4.86 | -0.38 | 7.03E-01 | 0.92 |
| ENSG00000280927.1 | CTBP1-AS | 213980_s_at,213979_s_at,203392_s_at, | -0.01 | 8.46 | 8.50 | -0.40 | 6.93E-01 | 0.92 |
| ENSG00000251676.1 | RP11-614F17.2 | 237770_at, | 0.01 | 2.74 | 2.72 | 0.38 | 7.02E-01 | 0.92 |
| ENSG00000227908.3 | FLJ31104 | 1557466_at, | 0.01 | 3.35 | 3.33 | 0.38 | 7.03E-01 | 0.92 |
| ENSG00000260841.1 | NA | 1556750_at, | -0.01 | 2.86 | 2.88 | -0.39 | 6.95E-01 | 0.92 |
| ENSG00000236901.4 | MIR600HG | 223522_at,221422_s_at, | 0.01 | 4.88 | 4.85 | 0.38 | 7.05E-01 | 0.92 |
| ENSG00000231542.1 | TAB3-AS1 | 1558518_at, | -0.01 | 3.73 | 3.75 | -0.39 | 6.97E-01 | 0.92 |
| ENSG00000276476.2 | LINC00540 | 1561367_a_at, | -0.01 | 3.27 | 3.29 | -0.38 | 7.06E-01 | 0.92 |
| ENSG00000232825.1 | RP5-896L10.1 | 1563904_at,231062_at,214304_x_at,212730_at, | 0.02 | 5.43 | 5.37 | 0.38 | 7.07E-01 | 0.92 |
| ENSG00000260564.1 | RP11-403N16.3 | 1561078_at, | 0.01 | 3.09 | 3.07 | 0.38 | 7.07E-01 | 0.92 |
| ENSG00000227811.2 | FAM212B-AS1 | 236036_at, | -0.01 | 4.06 | 4.08 | -0.37 | 7.11E-01 | 0.92 |
| ENSG00000254528.7 | RP11-728F11.4 | 240323_at, | -0.01 | 3.77 | 3.79 | -0.37 | 7.09E-01 | 0.92 |
| ENSG00000228061.5 | Z83001.1 | 241099_at,1570335_at, | -0.01 | 2.98 | 3.00 | -0.37 | 7.15E-01 | 0.92 |
| ENSG00000258525.1 | RP11-829H16.3 | 1554242_a_at, | 0.01 | 2.76 | 2.74 | 0.37 | 7.15E-01 | 0.92 |
| ENSG00000261822.1 | RP11-265N6.2 | 229208_at, | 0.01 | 3.41 | 3.39 | 0.37 | 7.09E-01 | 0.92 |
| ENSG00000226754.1 | RP5-1024G6.5 | 1560562_a_at, | 0.01 | 3.53 | 3.50 | 0.37 | 7.11E-01 | 0.92 |
| ENSG00000279166.1 | RP11-107E5.3 | 215713_at, | -0.01 | 2.61 | 2.63 | -0.37 | 7.14E-01 | 0.92 |
| ENSG00000227617.8 | CERS6-AS1 | 212446_s_at, | -0.01 | 7.63 | 7.71 | -0.37 | 7.13E-01 | 0.92 |
| ENSG00000233896.1 | RP4-684O24.5 | 206803_at, | 0.01 | 3.31 | 3.30 | 0.37 | 7.11E-01 | 0.92 |
| ENSG00000273387.1 | RP3-412A9.16 | 1554650_a_at,1554649_at, | 0.01 | 4.04 | 4.03 | 0.37 | 7.11E-01 | 0.92 |
| ENSG00000272977.1 | CTA-390C10.10 | 1557077_a_at, | 0.01 | 4.27 | 4.25 | 0.37 | 7.13E-01 | 0.92 |
| ENSG00000224514.2 | LINC00620 | 1563043_at, | -0.01 | 3.03 | 3.05 | -0.37 | 7.14E-01 | 0.92 |
| ENSG00000241696.1 | RP11-420J11.2 | 1558497_a_at,1558496_at, | -0.01 | 2.80 | 2.82 | -0.38 | 7.08E-01 | 0.92 |
| ENSG00000245928.2 | RP11-630D6.5 | 203915_at, | -0.03 | 7.53 | 7.69 | -0.38 | 7.08E-01 | 0.92 |
| ENSG00000253279.5 | FAM183CP | 1569887_a_at, | 0.00 | 4.41 | 4.40 | 0.37 | 7.13E-01 | 0.92 |
| ENSG00000261409.1 | RP6-24A23.7 | 1560652_at, | 0.01 | 2.78 | 2.76 | 0.37 | 7.12E-01 | 0.92 |
| ENSG00000236963.5 | NA | 209448_at,234563_at,1554514_at, | 0.01 | 5.17 | 5.14 | 0.36 | 7.18E-01 | 0.92 |
| ENSG00000228853.1 | NEGR1-IT1 | 241263_at,219027_s_at,208385_at,226203_at, | 0.00 | 5.51 | 5.53 | -0.36 | 7.20E-01 | 0.92 |
| ENSG00000267134.1 | RP11-146N18.1 | 1561455_at,1561090_at, | -0.01 | 3.34 | 3.37 | -0.36 | 7.19E-01 | 0.92 |
| ENSG00000268595.1 | CTD-3187F8.2 | 1553887_at, | -0.01 | 2.96 | 2.98 | -0.36 | 7.18E-01 | 0.92 |
| ENSG00000234880.1 | LINC00163 | 1562034_at, | 0.01 | 4.15 | 4.13 | 0.36 | 7.20E-01 | 0.92 |
| ENSG00000230736.2 | RP1-149A16.3 | 234385_at, | 0.01 | 2.77 | 2.76 | 0.36 | 7.18E-01 | 0.92 |
| ENSG00000231177.4 | LINC00852 | 223725_at, | -0.01 | 3.23 | 3.24 | -0.36 | 7.18E-01 | 0.92 |
| ENSG00000242512.8 | LINC01206 | 231482_at, | 0.01 | 3.73 | 3.71 | 0.36 | 7.18E-01 | 0.92 |
| ENSG00000277855.1 | RP11-154H23.4 | 1566893_at, | -0.01 | 2.62 | 2.63 | -0.36 | 7.18E-01 | 0.92 |
| ENSG00000171889.3 | MIR31HG | 1553574_at,240670_at, | 0.01 | 3.26 | 3.24 | 0.36 | 7.19E-01 | 0.92 |
| ENSG00000259877.2 | RP11-46C24.7 | 1554520_at, | 0.01 | 3.79 | 3.77 | 0.36 | 7.21E-01 | 0.92 |
| ENSG00000270066.3 | SCARNA2 | 237137_at, | -0.01 | 5.65 | 5.70 | -0.36 | 7.23E-01 | 0.92 |
| ENSG00000250786.1 | SNHG18 | 227655_at, | -0.01 | 4.39 | 4.43 | -0.36 | 7.22E-01 | 0.92 |
| ENSG00000253197.5 | CTD-3239E11.2 | 225603_s_at,225599_s_at,224158_s_at,1562066_at, | 0.01 | 4.51 | 4.48 | 0.36 | 7.23E-01 | 0.92 |
| ENSG00000235927.4 | NEXN-AS1 | 1560746_at, | -0.01 | 2.40 | 2.41 | -0.35 | 7.24E-01 | 0.92 |
| ENSG00000229699.1 | RP1-140J1.1 | 1561345_at, | -0.01 | 3.12 | 3.14 | -0.35 | 7.27E-01 | 0.92 |
| ENSG00000229431.1 | RP1-92O14.6 | 201376_s_at, | -0.01 | 7.11 | 7.16 | -0.35 | 7.27E-01 | 0.92 |
| ENSG00000232377.1 | AC016910.1 | 240548_at,219770_at,239390_at, | -0.01 | 4.45 | 4.47 | -0.35 | 7.27E-01 | 0.92 |
| ENSG00000241956.9 | CTC-340A15.2 | 1560491_at, | 0.01 | 2.63 | 2.61 | 0.35 | 7.27E-01 | 0.92 |
| ENSG00000253379.5 | RP11-1102P16.1 | 1569736_at, | 0.01 | 3.07 | 3.05 | 0.35 | 7.27E-01 | 0.92 |
| ENSG00000259723.1 | RP5-823G15.5 | 244521_at,235616_at, | -0.02 | 5.02 | 5.08 | -0.35 | 7.28E-01 | 0.92 |
| ENSG00000237187.8 | NR2F1-AS1 | 1556695_a_at,1556696_s_at, | -0.01 | 3.00 | 3.03 | -0.35 | 7.28E-01 | 0.92 |
| ENSG00000236200.5 | KDM4A-AS1 | 203205_at, | -0.01 | 5.59 | 5.61 | -0.35 | 7.30E-01 | 0.92 |
| ENSG00000225313.5 | RP11-415J8.3 | 240738_at,216811_at,216812_at,242686_at, | -0.01 | 3.33 | 3.35 | -0.34 | 7.32E-01 | 0.92 |
| ENSG00000224613.6 | RP11-153F1.1 | 1554367_at,212424_at, | 0.00 | 6.82 | 6.84 | -0.35 | 7.30E-01 | 0.92 |
| ENSG00000270108.1 | RP11-73M18.6 | 239793_at, | -0.01 | 3.15 | 3.17 | -0.34 | 7.32E-01 | 0.92 |
| ENSG00000260871.1 | CTD-2373J6.1 | 1556392_a_at, | 0.01 | 2.59 | 2.58 | 0.34 | 7.31E-01 | 0.92 |
| ENSG00000250049.5 | RP11-348J24.2 | 241566_at, | -0.02 | 3.04 | 3.08 | -0.34 | 7.31E-01 | 0.92 |
| ENSG00000273112.1 | RP11-25K21.6 | 117_at, | 0.01 | 5.07 | 5.04 | 0.34 | 7.34E-01 | 0.92 |
| ENSG00000248441.6 | LINC01197 | 1562901_at, | 0.01 | 2.83 | 2.82 | 0.34 | 7.33E-01 | 0.92 |
| ENSG00000263753.6 | LINC00667 | 215283_at, | -0.01 | 5.70 | 5.73 | -0.34 | 7.33E-01 | 0.92 |
| ENSG00000248049.6 | UBA6-AS1 | 204013_s_at, | -0.01 | 3.14 | 3.17 | -0.34 | 7.34E-01 | 0.92 |
| ENSG00000280222.1 | RP11-174G17.3 | 234063_at,234012_at, | -0.01 | 4.08 | 4.10 | -0.34 | 7.35E-01 | 0.92 |
| ENSG00000264116.4 | RP11-321M21.3 | 1556288_at, | 0.01 | 2.58 | 2.57 | 0.34 | 7.35E-01 | 0.92 |
| ENSG00000269038.1 | AP001462.6 | 1563426_a_at, | 0.01 | 5.38 | 5.33 | 0.33 | 7.39E-01 | 0.92 |
| ENSG00000245105.2 | A2M-AS1 | 1564139_at,1558450_at, | 0.01 | 4.12 | 4.10 | 0.33 | 7.39E-01 | 0.92 |
| ENSG00000258378.1 | RP11-517O13.1 | 218316_at, | 0.01 | 6.40 | 6.37 | 0.33 | 7.39E-01 | 0.92 |
| ENSG00000265148.5 | BZRAP1-AS1 | 218704_at,228826_at,239588_s_at,244226_s_at,231038_s_at, | -0.01 | 4.05 | 4.07 | -0.33 | 7.39E-01 | 0.92 |
| ENSG00000235335.2 | AC016723.4 | 222969_at,220829_s_at, | -0.01 | 3.04 | 3.05 | -0.34 | 7.37E-01 | 0.92 |
| ENSG00000236858.1 | CTA-992D9.6 | 1562030_at, | -0.01 | 3.85 | 3.86 | -0.34 | 7.37E-01 | 0.92 |
| ENSG00000270194.1 | RP11-259K5.2 | 1553775_at, | 0.01 | 4.05 | 4.04 | 0.33 | 7.39E-01 | 0.92 |
| ENSG00000261773.1 | WI2-89031B12.1 | 1570297_at, | -0.01 | 3.84 | 3.86 | -0.34 | 7.36E-01 | 0.92 |
| ENSG00000261971.6 | MMP25-AS1 | 228085_at, | 0.01 | 3.40 | 3.38 | 0.33 | 7.40E-01 | 0.92 |
| ENSG00000230250.1 | NA | 241521_at,208521_at,1569428_at, | -0.01 | 3.76 | 3.78 | -0.33 | 7.43E-01 | 0.93 |
| ENSG00000267282.1 | CTB-129P6.4 | 1566870_at,232078_at,225418_at, | -0.01 | 5.20 | 5.23 | -0.33 | 7.44E-01 | 0.93 |
| ENSG00000259357.2 | RP11-316M1.12 | 222212_s_at, | -0.01 | 9.07 | 9.13 | -0.32 | 7.46E-01 | 0.93 |
| ENSG00000274020.1 | LINC01138 | 1562957_at,1556457_s_at,237173_at, | -0.01 | 2.55 | 2.56 | -0.32 | 7.46E-01 | 0.93 |
| ENSG00000259240.1 | RP11-108K3.1 | 239459_s_at,1554296_at,234549_at, | 0.01 | 3.74 | 3.72 | 0.32 | 7.47E-01 | 0.93 |
| ENSG00000259700.3 | RP11-485O10.3 | 237129_at, | 0.01 | 3.14 | 3.12 | 0.33 | 7.45E-01 | 0.93 |
| ENSG00000224730.1 | AC009892.10 | 229937_x_at, | -0.02 | 4.85 | 4.91 | -0.32 | 7.47E-01 | 0.93 |
| ENSG00000249085.1 | CTD-2631K10.1 | 1570633_at,1570632_at, | -0.01 | 2.53 | 2.54 | -0.32 | 7.46E-01 | 0.93 |
| ENSG00000226167.1 | AP4B1-AS1 | 231962_at,241522_at, | 0.01 | 3.00 | 2.99 | 0.32 | 7.49E-01 | 0.93 |
| ENSG00000251562.7 | MALAT1 | 224558_s_at,1558678_s_at,223578_x_at,224568_x_at,  227510_x_at,228582_x_at,224559_at,223940_x_at,  223577_x_at, | -0.01 | 9.18 | 9.22 | -0.32 | 7.53E-01 | 0.93 |
| ENSG00000268635.2 | RP11-739B23.1 | 1565771_at, | -0.01 | 4.40 | 4.42 | -0.31 | 7.54E-01 | 0.93 |
| ENSG00000254907.1 | RP11-484D2.2 | 214960_at,229692_at,233078_at,201687_s_at, | -0.01 | 5.56 | 5.60 | -0.32 | 7.52E-01 | 0.93 |
| ENSG00000254458.1 | RP11-867G23.13 | 219025_at, | -0.01 | 7.60 | 7.64 | -0.32 | 7.50E-01 | 0.93 |
| ENSG00000256196.1 | RP11-881M11.4 | 201361_at, | -0.01 | 7.27 | 7.31 | -0.32 | 7.49E-01 | 0.93 |
| ENSG00000244926.6 | ALKBH3-AS1 | 1557564_at,1557565_a_at, | 0.01 | 2.99 | 2.98 | 0.32 | 7.51E-01 | 0.93 |
| ENSG00000246863.2 | RP11-325N19.3 | 1566081_at,215981_at, | 0.01 | 2.89 | 2.88 | 0.32 | 7.51E-01 | 0.93 |
| ENSG00000236782.5 | NA | 202533_s_at, | 0.01 | 3.96 | 3.93 | 0.31 | 7.55E-01 | 0.93 |
| ENSG00000213904.8 | LIPE-AS1 | 206576_s_at,206676_at,209498_at, | 0.01 | 4.53 | 4.49 | 0.31 | 7.54E-01 | 0.93 |
| ENSG00000225420.1 | AC104134.2 | 218696_at, | -0.01 | 6.97 | 7.02 | -0.32 | 7.49E-01 | 0.93 |
| ENSG00000256628.3 | ZBTB11-AS1 | 232259_s_at, | 0.01 | 5.33 | 5.30 | 0.31 | 7.55E-01 | 0.93 |
| ENSG00000273125.1 | RP11-115H18.1 | 1557017_at, | 0.00 | 2.35 | 2.36 | -0.32 | 7.49E-01 | 0.93 |
| ENSG00000245526.8 | LINC00461 | 238850_at,230902_at,230272_at,1561392_at, | -0.01 | 3.16 | 3.18 | -0.31 | 7.55E-01 | 0.93 |
| ENSG00000237159.5 | CNTFR-AS1 | 1556771_a_at, | -0.01 | 3.30 | 3.31 | -0.32 | 7.53E-01 | 0.93 |
| ENSG00000259849.1 | VENTXP1 | 216726_at, | -0.01 | 3.35 | 3.37 | -0.32 | 7.50E-01 | 0.93 |
| ENSG00000183562.3 | CTC-343N3.1 | 220514_at, | 0.01 | 3.68 | 3.66 | 0.31 | 7.58E-01 | 0.93 |
| ENSG00000261240.1 | RP11-304L19.4 | 241090_at, | -0.01 | 3.32 | 3.34 | -0.31 | 7.56E-01 | 0.93 |
| ENSG00000263388.1 | CTC-297N7.10 | 223221_at, | 0.01 | 5.08 | 5.04 | 0.31 | 7.57E-01 | 0.93 |
| ENSG00000267476.1 | RP11-126O1.4 | 208309_s_at,238157_at, | -0.01 | 3.97 | 3.99 | -0.31 | 7.58E-01 | 0.93 |
| ENSG00000214145.6 | LINC00887 | 1564485_at, | -0.01 | 3.02 | 3.04 | -0.31 | 7.58E-01 | 0.93 |
| ENSG00000231160.9 | KLF3-AS1 | 1570414_x_at, | -0.01 | 3.44 | 3.46 | -0.31 | 7.56E-01 | 0.93 |
| ENSG00000257524.5 | RP11-203J24.9 | 222571_at, | 0.01 | 5.78 | 5.76 | 0.31 | 7.59E-01 | 0.93 |
| ENSG00000247699.2 | CTB-127C13.1 | 1566126_at, | -0.01 | 2.80 | 2.82 | -0.31 | 7.59E-01 | 0.93 |
| ENSG00000255314.1 | NA | 1563086_at, | 0.00 | 4.36 | 4.37 | -0.30 | 7.64E-01 | 0.93 |
| ENSG00000230426.2 | ERVMER61-1 | 1561331_at, | 0.00 | 2.39 | 2.40 | -0.30 | 7.64E-01 | 0.93 |
| ENSG00000235875.3 | ARHGEF7-AS2 | 1557439_at, | -0.01 | 2.60 | 2.61 | -0.30 | 7.65E-01 | 0.93 |
| ENSG00000245482.2 | RP11-847H18.2 | 1552306_at,1552304_at, | -0.01 | 3.08 | 3.10 | -0.30 | 7.67E-01 | 0.94 |
| ENSG00000235904.1 | RBMS3-AS3 | 237329_at, | -0.01 | 2.60 | 2.61 | -0.30 | 7.67E-01 | 0.94 |
| ENSG00000277511.1 | CTD-2095E4.5 | 230314_at, | -0.01 | 3.70 | 3.72 | -0.30 | 7.68E-01 | 0.94 |
| ENSG00000232633.4 | CTD-2201G3.1 | 211694_at, | 0.01 | 3.40 | 3.39 | 0.30 | 7.68E-01 | 0.94 |
| ENSG00000231918.1 | AC007682.1 | 229186_s_at,242759_at,236501_at,212062_at,233706_at,  219536_s_at,224542_s_at,229661_at, | 0.00 | 4.78 | 4.76 | 0.29 | 7.69E-01 | 0.94 |
| ENSG00000214324.5 | C3orf56 | 1562762_at, | -0.01 | 2.92 | 2.94 | -0.29 | 7.73E-01 | 0.94 |
| ENSG00000241336.1 | LINC01487 | 1557498_a_at, | -0.01 | 2.71 | 2.72 | -0.29 | 7.73E-01 | 0.94 |
| ENSG00000251045.1 | CTC-321K16.4 | 1562617_at, | 0.01 | 3.50 | 3.49 | 0.29 | 7.73E-01 | 0.94 |
| ENSG00000259940.2 | CTD-3203P2.1 | 237229_at, | 0.01 | 3.37 | 3.35 | 0.29 | 7.74E-01 | 0.94 |
| ENSG00000236436.1 | AC012361.1 | 1562860_at, | 0.00 | 2.48 | 2.49 | -0.29 | 7.76E-01 | 0.94 |
| ENSG00000228395.1 | RP11-216B9.6 | 225371_at,206921_at, | -0.01 | 4.31 | 4.33 | -0.28 | 7.77E-01 | 0.94 |
| ENSG00000273828.1 | RP11-394O2.3 | 1554857_at,221528_s_at, | 0.01 | 5.28 | 5.25 | 0.28 | 7.80E-01 | 0.94 |
| ENSG00000253510.1 | RP5-991O23.1 | 1561256_at, | 0.01 | 3.23 | 3.21 | 0.28 | 7.80E-01 | 0.94 |
| ENSG00000243818.4 | RP11-372E1.4 | 1556387_at, | -0.01 | 2.86 | 2.87 | -0.28 | 7.81E-01 | 0.95 |
| ENSG00000280832.1 | ST3GAL4-AS1 | 1555974_a_at,1555973_at, | -0.01 | 3.66 | 3.69 | -0.28 | 7.82E-01 | 0.95 |
| ENSG00000235872.2 | RP11-335O4.3 | 202971_s_at, | -0.01 | 5.22 | 5.25 | -0.28 | 7.83E-01 | 0.95 |
| ENSG00000255145.2 | STX17-AS1 | 1558689_a_at, | 0.01 | 3.07 | 3.06 | 0.28 | 7.84E-01 | 0.95 |
| ENSG00000281041.2 | NA | 228679_at,229196_at,212611_at,239368_at,226841_at,  210712_at,231432_at, | -0.01 | 4.36 | 4.37 | -0.27 | 7.88E-01 | 0.95 |
| ENSG00000272438.1 | RP11-54O7.16 | 211992_at, | -0.01 | 7.87 | 7.90 | -0.27 | 7.85E-01 | 0.95 |
| ENSG00000274698.1 | RP11-71L14.4 | 244096_at, | 0.00 | 2.49 | 2.48 | 0.27 | 7.87E-01 | 0.95 |
| ENSG00000223960.6 | AC009948.5 | 228714_at,228620_at,237105_at, | -0.01 | 3.88 | 3.90 | -0.27 | 7.86E-01 | 0.95 |
| ENSG00000205930.8 | C21orf62-AS1 | 1559755_at,223923_at, | 0.00 | 4.79 | 4.78 | 0.27 | 7.87E-01 | 0.95 |
| ENSG00000241570.8 | PAQR9-AS1 | 229338_at,1558322_a_at, | 0.00 | 2.90 | 2.91 | -0.27 | 7.87E-01 | 0.95 |
| ENSG00000274956.2 | UG0898H09 | 1558388_a_at,1558387_at, | 0.00 | 2.49 | 2.48 | 0.27 | 7.88E-01 | 0.95 |
| ENSG00000235106.8 | LINC00094 | 203825_at,203245_s_at,212547_at,213788_s_at, | -0.01 | 7.07 | 7.10 | -0.27 | 7.88E-01 | 0.95 |
| ENSG00000277215.1 | SPANXA2-OT1 | 1564491_at, | -0.01 | 2.79 | 2.80 | -0.27 | 7.86E-01 | 0.95 |
| ENSG00000260360.1 | RP11-533E19.5 | 1568832_a_at, | -0.01 | 2.85 | 2.86 | -0.26 | 7.94E-01 | 0.95 |
| ENSG00000259776.1 | RP11-544D21.2 | 1561679_at, | 0.01 | 3.18 | 3.16 | 0.27 | 7.90E-01 | 0.95 |
| ENSG00000255537.1 | AP000708.1 | 1555002_at, | 0.00 | 2.51 | 2.52 | -0.26 | 7.93E-01 | 0.95 |
| ENSG00000276272.1 | RP11-121C6.5 | 1569579_at, | 0.01 | 5.38 | 5.36 | 0.26 | 7.94E-01 | 0.95 |
| ENSG00000258701.1 | LINC00638 | 1553780_at, | 0.00 | 5.57 | 5.55 | 0.26 | 7.96E-01 | 0.95 |
| ENSG00000261669.1 | CTD-2515A14.1 | 233836_at, | -0.01 | 5.13 | 5.15 | -0.26 | 7.95E-01 | 0.95 |
| ENSG00000228384.4 | AC007040.6 | 1559675_at, | -0.01 | 3.34 | 3.35 | -0.27 | 7.91E-01 | 0.95 |
| ENSG00000232084.5 | LINC01104 | 1563679_at, | 0.01 | 3.22 | 3.20 | 0.26 | 7.92E-01 | 0.95 |
| ENSG00000259974.2 | LINC00261 | 228004_at, | 0.01 | 3.53 | 3.52 | 0.26 | 7.93E-01 | 0.95 |
| ENSG00000224832.1 | AP000469.2 | 1562634_at, | -0.01 | 2.55 | 2.56 | -0.26 | 7.95E-01 | 0.95 |
| ENSG00000226567.1 | LINC00606 | 1556447_at, | 0.00 | 2.48 | 2.48 | 0.27 | 7.91E-01 | 0.95 |
| ENSG00000279130.1 | RP11-278J6.4 | 241492_at, | -0.01 | 2.96 | 2.97 | -0.27 | 7.91E-01 | 0.95 |
| ENSG00000225230.1 | AC008937.3 | 240840_s_at, | 0.00 | 4.19 | 4.18 | 0.26 | 7.94E-01 | 0.95 |
| ENSG00000246366.6 | LACTB2-AS1 | 241197_at,1559264_at,218701_at, | -0.01 | 3.39 | 3.40 | -0.26 | 7.95E-01 | 0.95 |
| ENSG00000246662.6 | LINC00535 | 238244_at, | 0.01 | 3.43 | 3.41 | 0.26 | 7.96E-01 | 0.95 |
| ENSG00000266289.1 | RP11-1C8.6 | 1561739_at, | 0.01 | 3.41 | 3.40 | 0.26 | 7.97E-01 | 0.95 |
| ENSG00000268575.1 | RP1-283E3.8 | 227886_at, | 0.00 | 5.32 | 5.30 | 0.26 | 7.98E-01 | 0.95 |
| ENSG00000251018.2 | HMMR-AS1 | 207165_at, | 0.01 | 5.81 | 5.76 | 0.26 | 7.98E-01 | 0.95 |
| ENSG00000267128.1 | RP11-449J21.5 | 230776_at, | 0.01 | 3.20 | 3.18 | 0.25 | 7.99E-01 | 0.95 |
| ENSG00000182873.5 | RP11-181G12.2 | 220846_s_at, | 0.00 | 4.64 | 4.65 | -0.25 | 8.03E-01 | 0.95 |
| ENSG00000229404.5 | LINC00858 | 1563821_at, | 0.00 | 2.83 | 2.82 | 0.25 | 8.03E-01 | 0.95 |
| ENSG00000245311.2 | ARNTL2-AS1 | 223586_at, | 0.01 | 4.21 | 4.17 | 0.25 | 8.01E-01 | 0.95 |
| ENSG00000206573.8 | THUMPD3-AS1 | 225741_at,1555858_at,1555860_x_at, | -0.01 | 4.96 | 4.99 | -0.25 | 8.03E-01 | 0.95 |
| ENSG00000254578.1 | CTD-2517M22.16 | 223421_at, | 0.01 | 7.49 | 7.46 | 0.25 | 8.00E-01 | 0.95 |
| ENSG00000261334.1 | RP11-65J3.14 | 1564264_at, | 0.00 | 3.99 | 4.00 | -0.25 | 8.02E-01 | 0.95 |
| ENSG00000215560.2 | TTTY5 | 224040_at, | 0.00 | 3.68 | 3.67 | 0.25 | 8.02E-01 | 0.95 |
| ENSG00000257831.1 | RP11-596D21.1 | 226510_at, | -0.01 | 5.43 | 5.47 | -0.25 | 8.04E-01 | 0.95 |
| ENSG00000246379.6 | RP11-461O7.1 | 1560707_at, | 0.01 | 3.05 | 3.04 | 0.25 | 8.04E-01 | 0.95 |
| ENSG00000261076.1 | RP11-179B15.6 | 1569953_at, | -0.01 | 3.06 | 3.08 | -0.25 | 8.07E-01 | 0.95 |
| ENSG00000259720.1 | RP11-348B17.1 | 240386_at, | -0.01 | 2.69 | 2.70 | -0.25 | 8.07E-01 | 0.95 |
| ENSG00000267278.5 | MAP3K14-AS1 | 205192_at, | -0.01 | 5.08 | 5.11 | -0.25 | 8.07E-01 | 0.95 |
| ENSG00000251024.1 | RP11-203B7.1 | 241657_at,241655_at, | 0.00 | 2.87 | 2.87 | 0.25 | 8.06E-01 | 0.95 |
| ENSG00000237036.4 | ZEB1-AS1 | 229090_at, | -0.01 | 3.73 | 3.76 | -0.24 | 8.08E-01 | 0.95 |
| ENSG00000182912.6 | TSPEAR-AS2 | 1554954_at, | 0.00 | 3.56 | 3.54 | 0.24 | 8.09E-01 | 0.95 |
| ENSG00000259251.2 | RP11-643M14.1 | 1561424_at, | 0.01 | 3.07 | 3.05 | 0.24 | 8.09E-01 | 0.95 |
| ENSG00000234962.5 | LINC00700 | 1564209_at, | -0.01 | 2.83 | 2.84 | -0.24 | 8.11E-01 | 0.95 |
| ENSG00000261697.5 | RP11-178L8.5 | 1560241_at, | 0.00 | 3.27 | 3.25 | 0.24 | 8.11E-01 | 0.95 |
| ENSG00000237954.7 | RP11-14O19.2 | 207436_x_at, | 0.00 | 7.20 | 7.18 | 0.24 | 8.12E-01 | 0.95 |
| ENSG00000253658.5 | LINC01592 | 1561881_at, | 0.01 | 3.14 | 3.13 | 0.24 | 8.12E-01 | 0.95 |
| ENSG00000224347.6 | SCEL-AS1 | 1554920_at, | 0.00 | 2.29 | 2.29 | -0.24 | 8.14E-01 | 0.95 |
| ENSG00000223563.1 | AP001601.2 | 229357_at,219935_at, | -0.01 | 5.28 | 5.33 | -0.24 | 8.13E-01 | 0.95 |
| ENSG00000258499.1 | RP11-862G15.2 | 1562885_at, | 0.00 | 4.24 | 4.23 | 0.23 | 8.16E-01 | 0.95 |
| ENSG00000245694.8 | CRNDE | 238021_s_at, | 0.01 | 9.67 | 9.62 | 0.23 | 8.16E-01 | 0.95 |
| ENSG00000219665.8 | CTD-2006C1.2 | 1562069_at, | 0.00 | 2.93 | 2.92 | 0.23 | 8.15E-01 | 0.95 |
| ENSG00000247157.6 | LINC01252 | 1557826_at, | -0.01 | 3.92 | 3.95 | -0.23 | 8.17E-01 | 0.96 |
| ENSG00000257550.1 | RP11-793H13.3 | 232732_at, | 0.00 | 4.41 | 4.40 | 0.23 | 8.19E-01 | 0.96 |
| ENSG00000248525.2 | CTD-2001E22.1 | 221324_at, | 0.00 | 4.21 | 4.23 | -0.23 | 8.19E-01 | 0.96 |
| ENSG00000274718.1 | RP11-346C4.3 | 1556940_at, | 0.00 | 2.68 | 2.67 | 0.23 | 8.20E-01 | 0.96 |
| ENSG00000279021.1 | RP11-391L3.4 | 1555645_at, | 0.00 | 5.07 | 5.06 | 0.23 | 8.20E-01 | 0.96 |
| ENSG00000278192.1 | RP5-1056H1.2 | 1554281_at, | 0.00 | 2.40 | 2.39 | 0.23 | 8.21E-01 | 0.96 |
| ENSG00000261572.1 | RP11-384L8.1 | 241563_at, | 0.00 | 4.88 | 4.90 | -0.23 | 8.22E-01 | 0.96 |
| ENSG00000260930.1 | LINC01416 | 1561339_at, | 0.01 | 2.96 | 2.95 | 0.22 | 8.23E-01 | 0.96 |
| ENSG00000226235.1 | LEMD1-AS1 | 1561280_at, | 0.00 | 2.44 | 2.44 | -0.22 | 8.24E-01 | 0.96 |
| ENSG00000254671.2 | STT3A-AS1 | 216396_s_at, | -0.01 | 7.63 | 7.66 | -0.22 | 8.25E-01 | 0.96 |
| ENSG00000237054.9 | PRMT5-AS1 | 1564520_s_at, | 0.01 | 7.40 | 7.36 | 0.22 | 8.24E-01 | 0.96 |
| ENSG00000223546.6 | LINC00630 | 1569674_at, | 0.00 | 2.50 | 2.51 | -0.22 | 8.25E-01 | 0.96 |
| ENSG00000244342.5 | LINC00698 | 234667_at,1557369_a_at, | 0.01 | 3.69 | 3.68 | 0.22 | 8.26E-01 | 0.96 |
| ENSG00000279154.1 | RP11-345K9.2 | 216732_at, | 0.00 | 3.34 | 3.33 | 0.22 | 8.27E-01 | 0.96 |
| ENSG00000204025.6 | TRPC5OS | 231138_at, | -0.01 | 2.62 | 2.63 | -0.22 | 8.27E-01 | 0.96 |
| ENSG00000224459.1 | RP11-169K16.4 | 232245_at,208520_at, | 0.00 | 3.74 | 3.73 | 0.22 | 8.29E-01 | 0.96 |
| ENSG00000239636.1 | RP4-728D4.2 | 231323_at,224647_at,224649_x_at, | 0.00 | 4.83 | 4.84 | -0.22 | 8.28E-01 | 0.96 |
| ENSG00000203288.3 | RP11-98D18.9 | 1568853_at, | 0.00 | 5.13 | 5.12 | 0.22 | 8.28E-01 | 0.96 |
| ENSG00000255644.1 | RP11-59N23.1 | 205304_s_at, | 0.01 | 5.34 | 5.30 | 0.22 | 8.30E-01 | 0.96 |
| ENSG00000267749.1 | CTC-265F19.1 | 1566643_a_at,1566642_at, | 0.00 | 3.54 | 3.55 | -0.21 | 8.31E-01 | 0.96 |
| ENSG00000253607.1 | RP11-557C18.3 | 225488_at,222543_at,218172_s_at, | 0.00 | 6.83 | 6.86 | -0.21 | 8.30E-01 | 0.96 |
| ENSG00000234665.8 | RP11-262H14.3 | 237695_at, | 0.00 | 5.10 | 5.09 | 0.21 | 8.30E-01 | 0.96 |
| ENSG00000238164.6 | RP3-395M20.8 | 233582_at, | 0.00 | 4.00 | 3.99 | 0.20 | 8.38E-01 | 0.96 |
| ENSG00000260990.1 | RP3-518E13.2 | 206990_at,213841_at, | -0.01 | 3.58 | 3.60 | -0.21 | 8.38E-01 | 0.96 |
| ENSG00000272078.1 | RP4-734G22.3 | 220015_at, | 0.00 | 7.60 | 7.59 | 0.19 | 8.50E-01 | 0.96 |
| ENSG00000238272.1 | RP3-436N22.3 | 236421_at, | 0.00 | 2.69 | 2.68 | 0.20 | 8.43E-01 | 0.96 |
| ENSG00000279029.1 | RP11-144I2.1 | 224089_at, | -0.01 | 2.91 | 2.92 | -0.20 | 8.41E-01 | 0.96 |
| ENSG00000273262.1 | RP11-18I14.11 | 236713_at, | 0.00 | 4.68 | 4.67 | 0.19 | 8.48E-01 | 0.96 |
| ENSG00000236467.7 | NA | 1569763_at,1561962_at, | 0.00 | 3.43 | 3.42 | 0.20 | 8.44E-01 | 0.96 |
| ENSG00000273108.1 | RP11-416N2.4 | 230767_at, | -0.01 | 3.51 | 3.53 | -0.19 | 8.50E-01 | 0.96 |
| ENSG00000250230.2 | RP11-855O10.2 | 1562029_at, | 0.00 | 3.29 | 3.28 | 0.20 | 8.41E-01 | 0.96 |
| ENSG00000279233.1 | RP11-158L12.4 | 236722_at, | 0.00 | 5.70 | 5.68 | 0.21 | 8.37E-01 | 0.96 |
| ENSG00000257137.5 | C12orf80 | 1560276_at, | 0.00 | 3.25 | 3.26 | -0.20 | 8.41E-01 | 0.96 |
| ENSG00000229578.2 | LINC00358 | 1562949_at, | -0.01 | 2.60 | 2.61 | -0.19 | 8.49E-01 | 0.96 |
| ENSG00000258504.2 | RP11-638I2.6 | 225306_s_at,225305_at, | -0.01 | 7.29 | 7.32 | -0.20 | 8.43E-01 | 0.96 |
| ENSG00000258384.1 | AC068831.6 | 221708_s_at, | 0.00 | 7.48 | 7.46 | 0.21 | 8.33E-01 | 0.96 |
| ENSG00000259006.1 | RP11-566K11.4 | 205458_at, | 0.00 | 5.64 | 5.62 | 0.19 | 8.47E-01 | 0.96 |
| ENSG00000261685.2 | RP11-401P9.4 | 229613_at, | -0.01 | 4.30 | 4.32 | -0.19 | 8.49E-01 | 0.96 |
| ENSG00000233101.10 | HOXB-AS3 | 208414_s_at,231767_at,236893_at,236892_s_at, | -0.01 | 4.48 | 4.51 | -0.20 | 8.39E-01 | 0.96 |
| ENSG00000263293.2 | THCAT158 | 216261_at, | 0.00 | 3.58 | 3.57 | 0.21 | 8.34E-01 | 0.96 |
| ENSG00000237328.1 | RAI1-AS1 | 235862_at, | 0.00 | 5.66 | 5.67 | -0.19 | 8.50E-01 | 0.96 |
| ENSG00000256806.3 | NA | 229071_at, | -0.01 | 3.56 | 3.58 | -0.19 | 8.50E-01 | 0.96 |
| ENSG00000265533.1 | RP11-638L3.1 | 241656_at,241654_at, | 0.00 | 2.90 | 2.91 | -0.20 | 8.42E-01 | 0.96 |
| ENSG00000267322.2 | SNHG22 | 240006_at,225299_at, | 0.00 | 4.66 | 4.65 | 0.19 | 8.48E-01 | 0.96 |
| ENSG00000267858.5 | MZF1-AS1 | 222097_at,1556690_s_at,204138_s_at,210336_x_at,222098_s_at, | 0.00 | 5.12 | 5.13 | -0.19 | 8.51E-01 | 0.96 |
| ENSG00000237844.1 | AC092684.1 | 216618_at,216463_at, | 0.00 | 2.82 | 2.82 | -0.20 | 8.39E-01 | 0.96 |
| ENSG00000234028.3 | AC062029.1 | 241463_at, | 0.00 | 3.18 | 3.18 | -0.20 | 8.46E-01 | 0.96 |
| ENSG00000260059.1 | RP11-231E19.1 | 1561279_at, | 0.00 | 2.75 | 2.75 | -0.20 | 8.42E-01 | 0.96 |
| ENSG00000228203.6 | RNF144A-AS1 | 1559145_at, | 0.00 | 3.39 | 3.40 | -0.19 | 8.51E-01 | 0.96 |
| ENSG00000234684.6 | SDCBP2-AS1 | 236669_at,210187_at,1559622_at,200709_at, | 0.00 | 5.05 | 5.04 | 0.21 | 8.34E-01 | 0.96 |
| ENSG00000223843.4 | EFCAB6-AS1 | 233345_at, | 0.00 | 4.06 | 4.04 | 0.20 | 8.39E-01 | 0.96 |
| ENSG00000230513.1 | THAP7-AS1 | 1552698_at, | 0.00 | 4.60 | 4.61 | -0.19 | 8.49E-01 | 0.96 |
| ENSG00000235257.8 | ITGA9-AS1 | 1563635_at,241008_at, | 0.01 | 4.08 | 4.07 | 0.19 | 8.46E-01 | 0.96 |
| ENSG00000268324.2 | LRRC2-AS1 | 236731_at, | 0.00 | 3.12 | 3.13 | -0.21 | 8.33E-01 | 0.96 |
| ENSG00000251230.5 | MIR3945HG | 1559777_at, | 0.00 | 3.09 | 3.08 | 0.20 | 8.39E-01 | 0.96 |
| ENSG00000248771.5 | LINC01207 | 1564451_at, | -0.01 | 3.17 | 3.18 | -0.19 | 8.47E-01 | 0.96 |
| ENSG00000260786.1 | RP11-112L7.1 | 1561244_at, | 0.00 | 2.64 | 2.65 | -0.19 | 8.49E-01 | 0.96 |
| ENSG00000250309.2 | CTC-345K18.2 | 1562002_at,1562004_x_at, | 0.00 | 6.13 | 6.14 | -0.20 | 8.44E-01 | 0.96 |
| ENSG00000261693.1 | RP13-467H17.1 | 1561671_at, | 0.00 | 4.23 | 4.22 | 0.19 | 8.48E-01 | 0.96 |
| ENSG00000245857.2 | GS1-24F4.2 | 1559678_s_at, | 0.00 | 2.68 | 2.68 | 0.21 | 8.34E-01 | 0.96 |
| ENSG00000229140.8 | CCDC26 | 1553849_at, | -0.01 | 2.78 | 2.80 | -0.20 | 8.39E-01 | 0.96 |
| ENSG00000266947.1 | RP11-799D4.4 | 1557427_at, | 0.00 | 2.90 | 2.91 | -0.18 | 8.54E-01 | 0.96 |
| ENSG00000269793.5 | ZIM2-AS1 | 220653_at, | -0.01 | 4.24 | 4.26 | -0.18 | 8.54E-01 | 0.96 |
| ENSG00000261065.1 | RP11-74C13.4 | 219268_at, | -0.01 | 5.32 | 5.34 | -0.17 | 8.66E-01 | 0.97 |
| ENSG00000226419.6 | NA | 1568799_at,217570_x_at,1560893_at,236216_at,1562699_at,  220354_at,1559427_at, | 0.00 | 4.03 | 4.04 | -0.17 | 8.66E-01 | 0.97 |
| ENSG00000237233.2 | TMEM26-AS1 | 244412_at, | 0.00 | 2.59 | 2.58 | 0.17 | 8.68E-01 | 0.97 |
| ENSG00000272301.1 | RP11-111M22.4 | 229243_at, | 0.01 | 3.85 | 3.83 | 0.17 | 8.64E-01 | 0.97 |
| ENSG00000175728.4 | C11orf44 | 1553417_at, | 0.00 | 3.28 | 3.27 | 0.18 | 8.60E-01 | 0.97 |
| ENSG00000257596.1 | RP11-968A15.2 | 230752_at, | 0.00 | 3.00 | 2.99 | 0.17 | 8.67E-01 | 0.97 |
| ENSG00000225206.5 | NA | 211954_s_at,211953_s_at,244405_s_at, | 0.01 | 6.98 | 6.95 | 0.18 | 8.59E-01 | 0.97 |
| ENSG00000260495.1 | RP11-55K13.1 | 232886_at, | 0.00 | 3.14 | 3.13 | 0.17 | 8.65E-01 | 0.97 |
| ENSG00000270871.1 | AC015849.19 | 227884_at,227891_s_at, | 0.00 | 5.82 | 5.84 | -0.17 | 8.65E-01 | 0.97 |
| ENSG00000269653.1 | CTB-102L5.7 | 210922_at, | 0.00 | 4.29 | 4.28 | 0.17 | 8.69E-01 | 0.97 |
| ENSG00000279205.1 | RP11-632P5.1 | 1561480_a_at,1561479_at, | 0.00 | 3.17 | 3.17 | 0.17 | 8.65E-01 | 0.97 |
| ENSG00000225156.2 | AC012354.6 | 236695_at,231838_at, | 0.01 | 5.07 | 5.04 | 0.17 | 8.62E-01 | 0.97 |
| ENSG00000241728.5 | AP001062.8 | 203995_at,214309_s_at,203996_s_at, | 0.00 | 5.27 | 5.28 | -0.18 | 8.60E-01 | 0.97 |
| ENSG00000184385.2 | UMODL1-AS1 | 1553282_at, | 0.00 | 3.24 | 3.24 | -0.17 | 8.67E-01 | 0.97 |
| ENSG00000236499.2 | LINC00896 | 1553658_at, | 0.00 | 3.47 | 3.46 | 0.17 | 8.69E-01 | 0.97 |
| ENSG00000249417.1 | RP11-438D8.2 | 239475_at, | 0.00 | 3.69 | 3.68 | 0.17 | 8.66E-01 | 0.97 |
| ENSG00000273247.5 | RP11-83A24.2 | 1556382_a_at,1556381_at, | 0.00 | 3.91 | 3.90 | 0.17 | 8.68E-01 | 0.97 |
| ENSG00000229565.1 | AC108056.1 | 238251_at, | 0.00 | 2.87 | 2.88 | -0.18 | 8.61E-01 | 0.97 |
| ENSG00000224218.1 | RP11-359D14.3 | 228400_at, | 0.01 | 7.11 | 7.07 | 0.17 | 8.64E-01 | 0.97 |
| ENSG00000254102.1 | RP11-21C4.1 | 1563927_a_at, | 0.00 | 2.55 | 2.56 | -0.17 | 8.65E-01 | 0.97 |
| ENSG00000254777.5 | AC022182.1 | 244576_at, | 0.00 | 4.09 | 4.10 | -0.17 | 8.67E-01 | 0.97 |
| ENSG00000234129.7 | RP11-120D5.1 | 1563477_at, | 0.00 | 3.13 | 3.12 | 0.17 | 8.67E-01 | 0.97 |
| ENSG00000227947.1 | RP11-543D5.1 | 202738_s_at, | 0.01 | 6.33 | 6.30 | 0.16 | 8.70E-01 | 0.97 |
| ENSG00000258871.1 | RP3-514A23.2 | 211448_s_at,210270_at, | 0.00 | 4.67 | 4.68 | -0.16 | 8.71E-01 | 0.97 |
| ENSG00000277147.4 | LINC00869 | 1564204_at, | -0.01 | 3.65 | 3.67 | -0.13 | 8.93E-01 | 0.97 |
| ENSG00000231871.5 | IPO9-AS1 | 233567_at,224774_s_at, | 0.00 | 5.19 | 5.18 | 0.14 | 8.88E-01 | 0.97 |
| ENSG00000270171.1 | RP11-338N10.1 | 234693_at,234677_at, | 0.00 | 3.96 | 3.96 | 0.15 | 8.84E-01 | 0.97 |
| ENSG00000229656.6 | RP11-462L8.1 | 244864_at, | 0.00 | 4.05 | 4.04 | 0.14 | 8.90E-01 | 0.97 |
| ENSG00000204365.5 | C10orf126 | 1553915_at, | 0.00 | 2.35 | 2.35 | 0.14 | 8.92E-01 | 0.97 |
| ENSG00000255384.1 | RP11-770J1.4 | 211775_x_at, | 0.00 | 2.54 | 2.55 | -0.16 | 8.75E-01 | 0.97 |
| ENSG00000257261.5 | RP11-96H19.1 | 224214_at,1559817_at, | 0.00 | 3.39 | 3.39 | 0.15 | 8.78E-01 | 0.97 |
| ENSG00000196243.5 | LINC00615 | 1553497_at, | 0.00 | 2.69 | 2.69 | 0.15 | 8.78E-01 | 0.97 |
| ENSG00000278916.1 | CEP83-AS1 | 237021_at, | 0.00 | 4.13 | 4.13 | -0.15 | 8.85E-01 | 0.97 |
| ENSG00000256803.1 | RP11-133L14.5 | 231600_at, | 0.00 | 2.47 | 2.48 | -0.14 | 8.86E-01 | 0.97 |
| ENSG00000257176.2 | RP11-996F15.2 | 1557410_at, | 0.00 | 3.59 | 3.59 | -0.15 | 8.85E-01 | 0.97 |
| ENSG00000270015.1 | RP11-540B6.6 | 232016_at, | 0.00 | 4.13 | 4.13 | -0.14 | 8.89E-01 | 0.97 |
| ENSG00000259627.1 | RP11-244F12.2 | 210986_s_at, | 0.00 | 10.93 | 10.94 | -0.15 | 8.82E-01 | 0.97 |
| ENSG00000259330.1 | INAFM2 | 225567_at, | -0.01 | 4.15 | 4.17 | -0.14 | 8.92E-01 | 0.97 |
| ENSG00000276075.1 | CTD-2012K14.8 | 230548_at, | 0.00 | 3.95 | 3.94 | 0.16 | 8.76E-01 | 0.97 |
| ENSG00000238045.9 | AC009133.12 | 218300_at,227192_at, | 0.00 | 6.41 | 6.39 | 0.14 | 8.88E-01 | 0.97 |
| ENSG00000262999.1 | CTD-3088G3.6 | 1569767_at, | 0.00 | 5.10 | 5.10 | -0.16 | 8.75E-01 | 0.97 |
| ENSG00000280069.1 | CTD-2349P21.3 | 222214_at,234522_at, | 0.00 | 4.21 | 4.22 | -0.13 | 8.93E-01 | 0.97 |
| ENSG00000264859.5 | DSG2-AS1 | 1556834_at, | -0.01 | 2.68 | 2.69 | -0.15 | 8.78E-01 | 0.97 |
| ENSG00000277837.1 | RP11-714M23.2 | 237742_at, | 0.01 | 3.63 | 3.61 | 0.15 | 8.82E-01 | 0.97 |
| ENSG00000234546.2 | NA | 1562337_at, | 0.01 | 2.99 | 2.98 | 0.14 | 8.91E-01 | 0.97 |
| ENSG00000259436.1 | CTC-378H22.2 | 230041_at, | 0.00 | 5.13 | 5.14 | -0.15 | 8.81E-01 | 0.97 |
| ENSG00000241409.1 | AC064852.4 | 244582_at, | 0.00 | 2.84 | 2.84 | -0.15 | 8.80E-01 | 0.97 |
| ENSG00000231294.1 | AC006994.2 | 206069_s_at, | 0.00 | 3.43 | 3.42 | 0.14 | 8.89E-01 | 0.97 |
| ENSG00000279873.2 | LINC01126 | 1555124_at, | 0.00 | 3.77 | 3.76 | 0.14 | 8.89E-01 | 0.97 |
| ENSG00000231079.7 | AC105402.4 | 1562710_at, | 0.00 | 2.75 | 2.75 | -0.14 | 8.85E-01 | 0.97 |
| ENSG00000227107.1 | AC096574.5 | 238221_at, | 0.00 | 3.27 | 3.28 | -0.14 | 8.87E-01 | 0.97 |
| ENSG00000269202.1 | NA | 234340_at, | 0.00 | 2.87 | 2.88 | -0.15 | 8.80E-01 | 0.97 |
| ENSG00000226648.1 | PLCG1-AS1 | 1558354_s_at, | 0.00 | 2.68 | 2.69 | -0.14 | 8.85E-01 | 0.97 |
| ENSG00000233903.2 | Z83851.4 | 244754_at, | 0.00 | 4.42 | 4.41 | 0.13 | 8.93E-01 | 0.97 |
| ENSG00000225742.5 | RP11-513G11.4 | 240100_at,1557495_at, | 0.00 | 3.32 | 3.31 | 0.15 | 8.79E-01 | 0.97 |
| ENSG00000241679.2 | RP11-80H8.4 | 243478_at,239956_at, | 0.00 | 2.69 | 2.69 | -0.16 | 8.77E-01 | 0.97 |
| ENSG00000231249.1 | ITPR1-AS1 | 239764_at, | 0.01 | 3.36 | 3.34 | 0.14 | 8.85E-01 | 0.97 |
| ENSG00000250646.1 | RP11-530I17.1 | 203934_at, | 0.00 | 5.92 | 5.94 | -0.15 | 8.80E-01 | 0.97 |
| ENSG00000250544.1 | CTC-493L21.1 | 1557424_at, | 0.00 | 2.41 | 2.41 | 0.14 | 8.89E-01 | 0.97 |
| ENSG00000233828.3 | CTC-242N15.1 | 215187_at,234531_at,236765_at,220828_s_at,234528_at, | 0.00 | 3.56 | 3.57 | -0.16 | 8.74E-01 | 0.97 |
| ENSG00000245317.2 | CTC-241N9.1 | 1559534_at, | 0.00 | 2.67 | 2.67 | -0.14 | 8.86E-01 | 0.97 |
| ENSG00000204758.7 | CTC-308K20.1 | 233491_at, | 0.00 | 3.11 | 3.12 | -0.15 | 8.83E-01 | 0.97 |
| ENSG00000183154.1 | RP11-863K10.7 | 1561386_at, | 0.00 | 4.36 | 4.36 | 0.15 | 8.78E-01 | 0.97 |
| ENSG00000204860.4 | FAM201A | 1557014_a_at,1557541_at, | 0.00 | 2.92 | 2.93 | -0.14 | 8.87E-01 | 0.97 |
| ENSG00000225361.3 | PPP1R26-AS1 | 1562447_a_at, | 0.00 | 4.42 | 4.43 | -0.14 | 8.90E-01 | 0.97 |
| ENSG00000226334.1 | RP11-217B7.2 | 215876_at, | 0.00 | 3.51 | 3.52 | -0.14 | 8.85E-01 | 0.97 |
| ENSG00000246541.2 | RP11-363G15.2 | 1561217_at, | 0.00 | 3.01 | 3.02 | -0.13 | 8.94E-01 | 0.97 |
| ENSG00000254662.1 | RP11-872D17.4 | 200957_s_at, | 0.00 | 7.35 | 7.36 | -0.13 | 8.99E-01 | 0.97 |
| ENSG00000264630.5 | PRKCA-AS1 | 1555939_at, | 0.00 | 2.31 | 2.31 | -0.13 | 8.97E-01 | 0.97 |
| ENSG00000270540.1 | RP11-785G17.1 | 1561485_at, | 0.00 | 3.24 | 3.24 | -0.13 | 8.98E-01 | 0.97 |
| ENSG00000227418.6 | PCGEM1 | 234529_at, | 0.00 | 2.58 | 2.59 | -0.13 | 8.96E-01 | 0.97 |
| ENSG00000233723.7 | LINC01122 | 1557472_a_at,1562326_at,1556900_at,202549_at,230690_at,  235548_at,228173_at,217851_s_at,242975_s_at,242816_at,  208601_s_at,221500_s_at,225923_at,230535_s_at, | 0.00 | 4.80 | 4.80 | -0.13 | 8.96E-01 | 0.97 |
| ENSG00000226995.7 | LINC00658 | 231458_at, | 0.00 | 3.12 | 3.11 | 0.13 | 8.99E-01 | 0.97 |
| ENSG00000239523.5 | MYLK-AS1 | 1563466_at,224823_at, | 0.00 | 5.89 | 5.88 | 0.13 | 9.00E-01 | 0.97 |
| ENSG00000249476.1 | CTD-2587M2.1 | 1563489_at, | 0.00 | 3.61 | 3.61 | 0.13 | 8.99E-01 | 0.97 |
| ENSG00000253519.1 | AC106801.1 | 234641_at, | 0.00 | 2.46 | 2.47 | -0.13 | 8.96E-01 | 0.97 |
| ENSG00000282164.1 | PEG13 | 229108_at, | 0.00 | 4.79 | 4.78 | 0.13 | 8.99E-01 | 0.97 |
| ENSG00000279881.1 | RP11-513O13.1 | 1561988_at, | 0.00 | 3.75 | 3.74 | 0.13 | 9.00E-01 | 0.97 |
| ENSG00000248318.1 | RP11-713M15.1 | 1560449_at, | 0.00 | 3.38 | 3.38 | 0.12 | 9.01E-01 | 0.97 |
| ENSG00000238186.1 | RP11-656D10.6 | 219567_s_at, | 0.00 | 2.71 | 2.70 | 0.12 | 9.02E-01 | 0.97 |
| ENSG00000280195.1 | XX-FW83563B9.5 | 1569188_s_at, | 0.00 | 4.69 | 4.68 | 0.12 | 9.03E-01 | 0.97 |
| ENSG00000223774.5 | RP11-307B6.3 | 211562_s_at, | 0.00 | 3.40 | 3.40 | -0.12 | 9.04E-01 | 0.97 |
| ENSG00000237281.1 | CATIP-AS2 | 233177_s_at, | 0.01 | 5.62 | 5.60 | 0.12 | 9.05E-01 | 0.97 |
| ENSG00000255572.1 | RP11-273B20.3 | 231456_at, | 0.00 | 2.97 | 2.98 | -0.12 | 9.08E-01 | 0.97 |
| ENSG00000233755.1 | RP4-799D16.1 | 220879_at,221001_at,234386_s_at,229107_at,1564373_a_at,  226677_at, | 0.00 | 4.77 | 4.78 | -0.12 | 9.09E-01 | 0.97 |
| ENSG00000266872.1 | RP11-19P22.8 | 235252_at, | 0.00 | 6.52 | 6.54 | -0.12 | 9.08E-01 | 0.97 |
| ENSG00000263711.5 | RP11-169F17.1 | 1569264_at,216709_at,216703_at, | 0.00 | 3.00 | 3.00 | -0.11 | 9.09E-01 | 0.97 |
| ENSG00000259793.1 | RP11-400N9.1 | 1564056_at, | 0.00 | 3.40 | 3.39 | 0.12 | 9.07E-01 | 0.97 |
| ENSG00000236859.6 | NIFK-AS1 | 224713_at, | 0.00 | 6.39 | 6.38 | 0.12 | 9.07E-01 | 0.97 |
| ENSG00000269936.3 | RP11-394O4.5 | 227183_at, | 0.00 | 5.77 | 5.79 | -0.11 | 9.10E-01 | 0.97 |
| ENSG00000258634.3 | RP4-773N10.4 | 228944_at, | 0.00 | 4.26 | 4.27 | -0.11 | 9.11E-01 | 0.97 |
| ENSG00000261002.5 | RP11-546B15.1 | 212204_at,212202_s_at, | 0.00 | 8.38 | 8.36 | 0.11 | 9.11E-01 | 0.97 |
| ENSG00000260111.1 | RP11-529K1.4 | 229336_at, | 0.00 | 5.01 | 5.02 | -0.11 | 9.12E-01 | 0.97 |
| ENSG00000262151.1 | RP11-876N24.2 | 241520_x_at, | 0.00 | 6.02 | 6.01 | 0.11 | 9.12E-01 | 0.97 |
| ENSG00000261296.1 | RP11-299H22.6 | 237490_at, | 0.00 | 2.60 | 2.60 | 0.11 | 9.13E-01 | 0.97 |
| ENSG00000253302.1 | STAU2-AS1 | 227179_at, | 0.00 | 6.09 | 6.07 | 0.11 | 9.14E-01 | 0.98 |
| ENSG00000228127.1 | NA | 1561516_at, | 0.00 | 2.62 | 2.61 | 0.11 | 9.16E-01 | 0.98 |
| ENSG00000260167.1 | RP11-146F11.5 | 227294_at,227445_at, | 0.00 | 5.40 | 5.39 | 0.11 | 9.15E-01 | 0.98 |
| ENSG00000279041.1 | CTD-2373N4.3 | 231097_at, | 0.00 | 4.47 | 4.47 | 0.11 | 9.16E-01 | 0.98 |
| ENSG00000275672.1 | GATM-AS1 | 1566861_at,1566860_at, | 0.00 | 2.76 | 2.76 | -0.10 | 9.18E-01 | 0.98 |
| ENSG00000228274.3 | RP3-508I15.9 | 203450_at, | 0.00 | 8.14 | 8.13 | 0.10 | 9.18E-01 | 0.98 |
| ENSG00000250958.1 | LINC00492 | 1564281_at,1564282_a_at,1569923_s_at, | 0.00 | 3.99 | 3.99 | -0.10 | 9.17E-01 | 0.98 |
| ENSG00000229729.6 | RP11-159G9.5 | 1556744_a_at, | 0.00 | 5.80 | 5.81 | -0.10 | 9.21E-01 | 0.98 |
| ENSG00000229407.5 | RP11-12M5.3 | 209353_s_at, | 0.00 | 3.20 | 3.20 | -0.09 | 9.25E-01 | 0.98 |
| ENSG00000223764.2 | RP11-54O7.3 | 1555980_a_at,1555979_at, | 0.00 | 4.17 | 4.16 | 0.09 | 9.25E-01 | 0.98 |
| ENSG00000260852.1 | FBXL19-AS1 | 1553586_at, | 0.00 | 4.29 | 4.28 | 0.10 | 9.24E-01 | 0.98 |
| ENSG00000231532.5 | LINC01249 | 216342_x_at, | 0.00 | 11.36 | 11.37 | -0.10 | 9.24E-01 | 0.98 |
| ENSG00000258451.1 | RP11-903H12.3 | 205141_at, | 0.00 | 5.84 | 5.86 | -0.09 | 9.26E-01 | 0.98 |
| ENSG00000228794.8 | LINC01128 | 241965_at,219136_s_at, | 0.00 | 5.97 | 5.98 | -0.09 | 9.27E-01 | 0.98 |
| ENSG00000281731.2 | RP11-384K6.8 | 1560495_at, | 0.00 | 2.72 | 2.73 | -0.09 | 9.28E-01 | 0.98 |
| ENSG00000230817.5 | LINC01362 | 228281_at,209525_at,1558102_at,1552310_at,209524_at,  1558103_a_at,216693_x_at,215055_at,201759_at,216128_at, | 0.00 | 5.32 | 5.32 | 0.09 | 9.29E-01 | 0.98 |
| ENSG00000267107.6 | PCAT19 | 230132_at, | 0.00 | 4.54 | 4.54 | 0.09 | 9.30E-01 | 0.98 |
| ENSG00000229587.2 | RP11-197P3.5 | 1564690_at, | 0.00 | 2.48 | 2.47 | 0.09 | 9.30E-01 | 0.98 |
| ENSG00000260971.3 | RP11-504A18.1 | 1564807_at, | 0.00 | 2.55 | 2.54 | 0.09 | 9.31E-01 | 0.98 |
| ENSG00000261097.1 | LINC00563 | 215738_at, | 0.00 | 3.28 | 3.29 | -0.09 | 9.32E-01 | 0.98 |
| ENSG00000231671.1 | LINC01307 | 211262_at, | 0.00 | 3.06 | 3.05 | 0.09 | 9.32E-01 | 0.98 |
| ENSG00000265888.1 | DSCAS | 1556333_at,207324_s_at, | 0.00 | 2.77 | 2.78 | -0.08 | 9.34E-01 | 0.98 |
| ENSG00000239265.5 | CLRN1-AS1 | 1555035_a_at,1560432_at,241170_at, | 0.00 | 3.01 | 3.02 | -0.08 | 9.34E-01 | 0.98 |
| ENSG00000226330.1 | RP11-739N20.2 | 224692_at,204484_at, | 0.00 | 7.65 | 7.66 | -0.08 | 9.39E-01 | 0.99 |
| ENSG00000234807.5 | LINC01135 | 230527_at,213429_at,226908_at,217828_at, | 0.00 | 5.44 | 5.43 | 0.07 | 9.44E-01 | 0.99 |
| ENSG00000260460.1 | RP11-284F21.8 | 1561431_at, | 0.00 | 2.66 | 2.65 | 0.08 | 9.35E-01 | 0.99 |
| ENSG00000229294.1 | RP5-1044H5.1 | 1555547_at,1566482_at, | 0.00 | 2.46 | 2.46 | 0.06 | 9.55E-01 | 0.99 |
| ENSG00000235143.1 | RP1-65J11.5 | 1564401_at, | 0.00 | 3.79 | 3.78 | 0.06 | 9.52E-01 | 0.99 |
| ENSG00000224382.1 | LINC00703 | 1558660_at, | 0.00 | 3.36 | 3.36 | 0.06 | 9.56E-01 | 0.99 |
| ENSG00000225484.6 | NUTM2B-AS1 | 234695_x_at,1569538_at, | 0.00 | 4.63 | 4.63 | 0.07 | 9.44E-01 | 0.99 |
| ENSG00000280693.2 | SH3PXD2A-AS1 | 1557389_at, | 0.00 | 3.25 | 3.25 | -0.05 | 9.57E-01 | 0.99 |
| ENSG00000228826.2 | RP11-344P13.4 | 240913_at, | 0.00 | 3.42 | 3.43 | -0.06 | 9.51E-01 | 0.99 |
| ENSG00000223482.7 | NUTM2A-AS1 | 1558794_at, | 0.00 | 3.09 | 3.10 | -0.06 | 9.54E-01 | 0.99 |
| ENSG00000234942.2 | GRID1-AS1 | 1557874_at,1555268_a_at, | 0.00 | 3.63 | 3.63 | -0.05 | 9.59E-01 | 0.99 |
| ENSG00000237094.11 | RP4-669L17.10 | 221005_s_at,216798_at,219664_s_at,228613_at,226905_at, | 0.00 | 6.80 | 6.80 | 0.07 | 9.44E-01 | 0.99 |
| ENSG00000280143.1 | AP000892.6 | 203118_at, | 0.00 | 7.08 | 7.07 | 0.06 | 9.55E-01 | 0.99 |
| ENSG00000204241.7 | RP11-713P17.3 | 238872_at, | 0.00 | 3.35 | 3.35 | 0.05 | 9.57E-01 | 0.99 |
| ENSG00000249196.6 | RP11-669N7.2 | 1561757_a_at, | 0.00 | 3.03 | 3.02 | 0.06 | 9.55E-01 | 0.99 |
| ENSG00000258077.2 | RP11-114H23.1 | 1566499_at, | 0.00 | 3.15 | 3.15 | 0.07 | 9.42E-01 | 0.99 |
| ENSG00000272542.1 | RP11-255P5.2 | 233332_at, | 0.00 | 2.83 | 2.83 | -0.07 | 9.46E-01 | 0.99 |
| ENSG00000224743.6 | TEX26-AS1 | 227058_at,1565765_x_at, | 0.00 | 4.97 | 4.97 | 0.05 | 9.60E-01 | 0.99 |
| ENSG00000258742.5 | RP11-862G15.1 | 1562671_s_at,1562670_at, | 0.00 | 2.69 | 2.68 | 0.05 | 9.60E-01 | 0.99 |
| ENSG00000258399.6 | MEG8 | 241260_at,237945_at, | 0.00 | 2.97 | 2.98 | -0.06 | 9.50E-01 | 0.99 |
| ENSG00000280211.1 | RP11-2C24.3 | 206845_s_at,217642_at, | 0.00 | 5.80 | 5.80 | 0.05 | 9.58E-01 | 0.99 |
| ENSG00000260772.1 | RP11-311C24.1 | 224984_at, | 0.00 | 7.61 | 7.62 | -0.06 | 9.51E-01 | 0.99 |
| ENSG00000262117.5 | BCAR4 | 230854_at, | 0.01 | 3.38 | 3.36 | 0.07 | 9.45E-01 | 0.99 |
| ENSG00000260647.1 | RP1-178F10.1 | 1556784_at, | 0.00 | 3.68 | 3.68 | 0.07 | 9.46E-01 | 0.99 |
| ENSG00000266498.1 | RP11-45M22.5 | 224132_at, | 0.00 | 4.76 | 4.75 | 0.06 | 9.50E-01 | 0.99 |
| ENSG00000263494.1 | AC004702.2 | 228900_at,224105_x_at, | 0.00 | 5.02 | 5.02 | 0.08 | 9.39E-01 | 0.99 |
| ENSG00000264672.5 | SEPT4-AS1 | 210348_at, | 0.00 | 3.18 | 3.18 | 0.06 | 9.51E-01 | 0.99 |
| ENSG00000264464.1 | RP11-110H1.8 | 1562557_at, | 0.00 | 3.63 | 3.63 | 0.08 | 9.39E-01 | 0.99 |
| ENSG00000263724.1 | DLGAP1-AS3 | 1560522_at, | 0.00 | 2.82 | 2.82 | 0.07 | 9.41E-01 | 0.99 |
| ENSG00000268650.3 | AC068499.10 | 231478_at,206791_s_at, | 0.00 | 4.07 | 4.07 | -0.07 | 9.41E-01 | 0.99 |
| ENSG00000234773.6 | CTD-2666L21.1 | 233712_at, | 0.00 | 4.38 | 4.38 | -0.06 | 9.52E-01 | 0.99 |
| ENSG00000243819.4 | RN7SL832P | 1557584_at, | 0.00 | 5.10 | 5.10 | -0.06 | 9.50E-01 | 0.99 |
| ENSG00000236283.3 | AC013463.2 | 210432_s_at, | 0.00 | 2.95 | 2.94 | 0.06 | 9.50E-01 | 0.99 |
| ENSG00000228563.1 | AL133247.2 | 1558561_at, | 0.00 | 3.74 | 3.74 | -0.07 | 9.43E-01 | 0.99 |
| ENSG00000233048.1 | RP5-1069C8.2 | 1561678_at, | 0.00 | 3.03 | 3.02 | 0.06 | 9.55E-01 | 0.99 |
| ENSG00000255568.3 | BRWD1-AS2 | 1553069_at, | 0.00 | 2.89 | 2.89 | -0.05 | 9.59E-01 | 0.99 |
| ENSG00000240875.5 | LINC00886 | 244348_at, | 0.00 | 4.69 | 4.68 | 0.06 | 9.51E-01 | 0.99 |
| ENSG00000260244.1 | RP11-588K22.2 | 239580_at,227235_at, | 0.00 | 5.52 | 5.53 | -0.07 | 9.46E-01 | 0.99 |
| ENSG00000248692.5 | ADGRL3-AS1 | 236264_at, | 0.00 | 2.83 | 2.83 | -0.07 | 9.45E-01 | 0.99 |
| ENSG00000232021.6 | LEF1-AS1 | 243363_at,221557_s_at, | 0.00 | 3.62 | 3.62 | -0.06 | 9.55E-01 | 0.99 |
| ENSG00000196810.4 | CTBP1-AS2 | 237642_at, | 0.00 | 5.13 | 5.14 | -0.08 | 9.38E-01 | 0.99 |
| ENSG00000188242.4 | PP7080 | 225457_s_at,225458_at, | 0.00 | 5.33 | 5.32 | 0.07 | 9.42E-01 | 0.99 |
| ENSG00000251675.1 | CTC-458I2.2 | 212812_at, | 0.00 | 7.40 | 7.39 | 0.06 | 9.55E-01 | 0.99 |
| ENSG00000248309.5 | MEF2C-AS1 | 233522_at, | 0.00 | 3.85 | 3.84 | 0.08 | 9.40E-01 | 0.99 |
| ENSG00000250155.1 | CTD-2353F22.1 | 202800_at, | 0.00 | 6.15 | 6.14 | 0.07 | 9.48E-01 | 0.99 |
| ENSG00000279821.1 | RP11-1334A24.5 | 215534_at, | 0.00 | 3.64 | 3.64 | -0.07 | 9.44E-01 | 0.99 |
| ENSG00000255020.1 | AF131216.5 | 1559871_s_at, | 0.00 | 3.49 | 3.49 | -0.07 | 9.44E-01 | 0.99 |
| ENSG00000253116.1 | RP11-648L3.2 | 220898_at, | 0.00 | 4.52 | 4.50 | 0.07 | 9.47E-01 | 0.99 |
| ENSG00000261696.1 | RP11-354P17.15 | 1562880_at, | 0.00 | 2.52 | 2.52 | 0.05 | 9.58E-01 | 0.99 |
| ENSG00000261326.2 | LINC01355 | 228387_at,244490_at, | 0.00 | 4.73 | 4.73 | 0.05 | 9.62E-01 | 0.99 |
| ENSG00000234597.1 | AC010096.1 | 1561719_at, | 0.00 | 2.77 | 2.77 | 0.05 | 9.63E-01 | 0.99 |
| ENSG00000281376.1 | ABALON | 215037_s_at, | 0.00 | 3.57 | 3.56 | 0.05 | 9.63E-01 | 0.99 |
| ENSG00000224848.1 | RP11-535M15.1 | 237850_at, | 0.00 | 2.49 | 2.49 | -0.05 | 9.64E-01 | 0.99 |
| ENSG00000224597.9 | SVIL-AS1 | 230736_at,202565_s_at,238882_at,230737_s_at, | 0.00 | 5.36 | 5.36 | 0.04 | 9.64E-01 | 0.99 |
| ENSG00000228222.1 | AC074363.1 | 1562686_at,1570098_at,1562687_x_at, | 0.00 | 2.82 | 2.82 | -0.04 | 9.66E-01 | 0.99 |
| ENSG00000278611.1 | CTC-543D15.8 | 1561595_x_at, | 0.00 | 3.67 | 3.67 | -0.04 | 9.67E-01 | 0.99 |
| ENSG00000230523.1 | RP3-437I16.1 | 1562589_at,239108_at,240172_at, | 0.00 | 3.89 | 3.88 | 0.03 | 9.75E-01 | 0.99 |
| ENSG00000250135.1 | RP4-622L5.2 | 212101_at,226976_at,212103_at, | 0.00 | 6.95 | 6.94 | 0.03 | 9.75E-01 | 0.99 |
| ENSG00000234222.6 | RP11-315I20.1 | 225793_at,235036_at,1554602_at, | 0.00 | 4.82 | 4.82 | 0.03 | 9.75E-01 | 0.99 |
| ENSG00000255448.1 | RP1-59M18.2 | 237678_at, | 0.00 | 4.11 | 4.11 | -0.04 | 9.70E-01 | 0.99 |
| ENSG00000183643.4 | C15orf32 | 1553482_at, | 0.00 | 2.65 | 2.65 | 0.04 | 9.69E-01 | 0.99 |
| ENSG00000260465.1 | RP11-63M22.2 | 224614_at,224616_at, | 0.00 | 7.84 | 7.85 | -0.03 | 9.75E-01 | 0.99 |
| ENSG00000266208.1 | CTD-2267D19.3 | 230025_at, | 0.00 | 3.98 | 3.98 | -0.03 | 9.73E-01 | 0.99 |
| ENSG00000269082.1 | CTD-2620I22.1 | 206557_at, | 0.00 | 2.94 | 2.94 | 0.04 | 9.70E-01 | 0.99 |
| ENSG00000235997.2 | AC109642.1 | 1559605_a_at, | 0.00 | 3.33 | 3.33 | -0.03 | 9.74E-01 | 0.99 |
| ENSG00000226674.8 | TEX41 | 240492_at,237520_x_at, | 0.00 | 2.73 | 2.73 | 0.04 | 9.69E-01 | 0.99 |
| ENSG00000227308.2 | AC009502.4 | 1564336_at, | 0.00 | 3.95 | 3.95 | -0.04 | 9.70E-01 | 0.99 |
| ENSG00000228643.1 | AC079779.4 | 225091_at, | 0.00 | 6.63 | 6.63 | -0.03 | 9.73E-01 | 0.99 |
| ENSG00000242553.1 | AP001432.14 | 203635_at, | 0.00 | 5.92 | 5.92 | -0.04 | 9.72E-01 | 0.99 |
| ENSG00000197182.12 | MIRLET7BHG | 241464_s_at,232480_at,238144_s_at, | 0.00 | 4.74 | 4.74 | 0.03 | 9.74E-01 | 0.99 |
| ENSG00000235834.1 | RP1-60N8.1 | 1562822_at, | 0.00 | 3.17 | 3.17 | 0.04 | 9.72E-01 | 0.99 |
| ENSG00000234998.1 | RP11-439A17.10 | 1555967_at,227395_at, | 0.00 | 6.77 | 6.77 | 0.03 | 9.80E-01 | 0.99 |
| ENSG00000258119.1 | RP11-804F13.1 | 1560582_a_at, | 0.00 | 3.02 | 3.02 | -0.02 | 9.84E-01 | 0.99 |
| ENSG00000259954.1 | IL21R-AS1 | 237753_at,1559443_s_at,221658_s_at,219971_at, | 0.00 | 4.49 | 4.49 | -0.02 | 9.82E-01 | 0.99 |
| ENSG00000262703.1 | RP11-485G7.6 | 1563019_at, | 0.00 | 3.45 | 3.45 | 0.03 | 9.79E-01 | 0.99 |
| ENSG00000260293.2 | RP11-715J22.6 | 241411_at, | 0.00 | 4.84 | 4.84 | 0.02 | 9.82E-01 | 0.99 |
| ENSG00000225084.1 | AL450226.2 | 231459_at, | 0.00 | 3.80 | 3.80 | 0.02 | 9.81E-01 | 0.99 |
| ENSG00000274767.1 | AC131056.3 | 234616_at, | 0.00 | 2.69 | 2.69 | -0.03 | 9.78E-01 | 0.99 |
| ENSG00000261879.5 | RP11-333E1.1 | 1554666_at, | 0.00 | 4.18 | 4.18 | -0.02 | 9.85E-01 | 0.99 |
| ENSG00000269072.1 | CTD-3187F8.14 | 207224_s_at, | 0.00 | 3.74 | 3.74 | -0.02 | 9.80E-01 | 0.99 |
| ENSG00000222035.3 | AC079354.3 | 1556536_at, | 0.00 | 3.61 | 3.61 | 0.02 | 9.83E-01 | 0.99 |
| ENSG00000280397.1 | RP11-42F12.1 | 210703_at, | 0.00 | 2.57 | 2.57 | 0.02 | 9.83E-01 | 0.99 |
| ENSG00000248872.1 | RP11-344G13.1 | 1561228_at, | 0.00 | 3.69 | 3.69 | 0.03 | 9.77E-01 | 0.99 |
| ENSG00000248339.1 | RP11-717H13.1 | 234773_x_at, | 0.00 | 3.82 | 3.82 | -0.02 | 9.85E-01 | 0.99 |
| ENSG00000228737.2 | AC008781.7 | 216326_s_at, | 0.00 | 7.18 | 7.18 | -0.02 | 9.80E-01 | 0.99 |
| ENSG00000249669.7 | MIR143HG | 1558828_s_at, | 0.00 | 5.84 | 5.84 | -0.03 | 9.79E-01 | 0.99 |
| ENSG00000271828.1 | CTD-2310F14.1 | 1559652_at, | 0.00 | 2.39 | 2.39 | -0.02 | 9.85E-01 | 0.99 |
| ENSG00000254109.5 | RBPMS-AS1 | 240929_at, | 0.00 | 5.05 | 5.05 | -0.02 | 9.82E-01 | 0.99 |
| ENSG00000260995.1 | RP11-165H23.1 | 1561322_at, | 0.00 | 2.62 | 2.62 | -0.02 | 9.84E-01 | 0.99 |
| ENSG00000269821.1 | KCNQ1OT1 | 243435_at, | 0.00 | 6.50 | 6.50 | 0.02 | 9.88E-01 | 0.99 |
| ENSG00000279535.1 | AC113188.2 | 238673_at, | 0.00 | 6.47 | 6.47 | 0.02 | 9.88E-01 | 0.99 |
| ENSG00000261863.1 | RP11-141J13.5 | 1560513_at, | 0.00 | 4.18 | 4.18 | 0.01 | 9.89E-01 | 0.99 |
| ENSG00000257653.1 | RP11-579D7.2 | 209195_s_at, | 0.00 | 5.80 | 5.81 | -0.01 | 9.90E-01 | 0.99 |
| ENSG00000242808.7 | SOX2-OT | 214178_s_at,231898_x_at, | 0.00 | 3.37 | 3.37 | -0.01 | 9.92E-01 | 0.99 |
| ENSG00000237938.5 | RP11-288I21.1 | 1558670_at,228134_at,239307_at,218414_s_at,208543_at, | 0.00 | 4.90 | 4.90 | 0.01 | 9.93E-01 | 1.00 |
| ENSG00000267028.1 | TCF4-AS1 | 244480_at, | 0.00 | 3.19 | 3.19 | 0.01 | 9.94E-01 | 1.00 |
| ENSG00000196167.9 | COLCA1 | 235892_at, | 0.00 | 2.69 | 2.69 | -0.01 | 9.95E-01 | 1.00 |
| ENSG00000260855.1 | RP11-439E19.10 | 1569193_at, | 0.00 | 2.88 | 2.88 | -0.01 | 9.96E-01 | 1.00 |
| ENSG00000226252.1 | RP1-18D14.7 | 1561651_s_at,1561211_at, | 0.00 | 4.50 | 4.50 | 0.00 | 9.98E-01 | 1.00 |
| ENSG00000225953.2 | SATB2-AS1 | 1560010_a_at,235147_at, | 0.00 | 3.08 | 3.08 | 0.00 | 9.99E-01 | 1.00 |


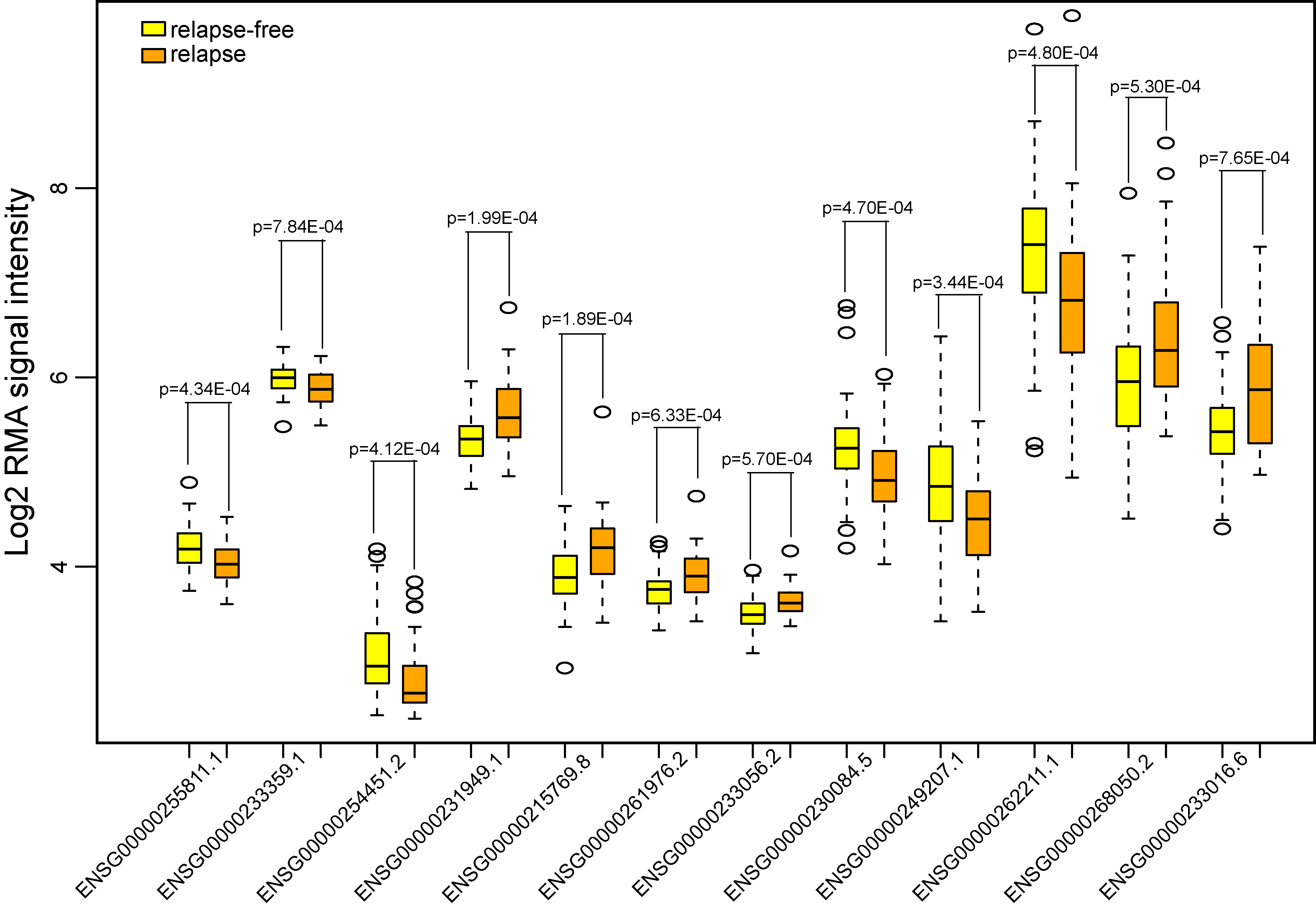


**Supplementary Figure S1.** Differentially expressed lncRNAs between the two patient groups who did and did not develop recurrence.

**Supplementary Table S3.** Significantly enriched functional clusters of GO terms and KEGG pathways

| GO terms and KEGG pathways | NO. of genes | P-value | Fold Enrichment |
| --- | --- | --- | --- |
| **PCGs positively correlated with lncRNA biomarkers** | | | |
| Cluster 1 (Enrichment Score: 11.01) | | | |
| GO:0045449~regulation of transcription | 290 | 1.85E-09 | 1.37 |
| GO:0006350~transcription | 242 | 3.90E-09 | 1.41 |
| GO:0006355~regulation of transcription, DNA-dependent | 192 | 1.75E-05 | 1.33 |
| GO:0051252~regulation of RNA metabolic process | 195 | 2.14E-05 | 1.32 |
| hsa03040:Spliceosome | 22 | 1.77E-04 | 2.45 |
| **PCGs negatively correlated with lncRNA biomarkers** | | | |
| Cluster 1 (Enrichment Score: 7.51) | | | |
| GO:0000278~mitotic cell cycle | 82 | 6.12E-13 | 2.31 |
| GO:0007049~cell cycle | 135 | 4.28E-12 | 1.82 |
| GO:0022402~cell cycle process | 101 | 6.95E-10 | 1.87 |
| GO:0022403~cell cycle phase | 76 | 3.80E-08 | 1.92 |
| GO:0048285~organelle fission | 49 | 1.37E-07 | 2.23 |
| GO:0000280~nuclear division | 47 | 2.69E-07 | 2.23 |
| GO:0007067~mitosis | 47 | 2.69E-07 | 2.23 |
| GO:0000087~M phase of mitotic cell cycle | 47 | 4.64E-07 | 2.19 |
| GO:0000279~M phase | 58 | 6.83E-06 | 1.84 |
| GO:0051301~cell division | 48 | 3.32E-04 | 1.70 |
| Cluster 2 (Enrichment Score: 3.81) | | | |
| GO:0046907~intracellular transport | 103 | 4.87E-07 | 1.64 |
| GO:0034613~cellular protein localization | 67 | 1.77E-05 | 1.70 |
| GO:0070727~cellular macromolecule localization | 67 | 2.28E-05 | 1.69 |
| GO:0006886~intracellular protein transport | 60 | 8.17E-05 | 1.67 |
| GO:0008104~protein localization | 119 | 9.03E-05 | 1.41 |
| GO:0006605~protein targeting | 38 | 3.08E-04 | 1.84 |
| GO:0015031~protein transport | 102 | 4.09E-04 | 1.40 |
| GO:0045184~establishment of protein localization | 102 | 5.63E-04 | 1.38 |
| GO:0033365~protein localization in organelle | 26 | 0.002927 | 1.86 |
| GO:0017038~protein import | 21 | 0.024185 | 1.67 |
| Cluster 3 (Enrichment Score: 3.81) | | | |
| GO:0051438~regulation of ubiquitin-protein ligase activity | 22 | 8.95E-06 | 2.94 |
| GO:0051248~negative regulation of protein metabolic process | 38 | 1.46E-05 | 2.12 |
| GO:0032269~negative regulation of cellular protein metabolic process | 37 | 1.48E-05 | 2.15 |
| GO:0051340~regulation of ligase activity | 22 | 1.69E-05 | 2.84 |
| GO:0051443~positive regulation of ubiquitin-protein ligase activity | 20 | 2.09E-05 | 2.98 |
| GO:0051439~regulation of ubiquitin-protein ligase activity during mitotic cell cycle | 20 | 2.60E-05 | 2.94 |
| GO:0032268~regulation of cellular protein metabolic process | 74 | 2.84E-05 | 1.63 |
| GO:0031398~positive regulation of protein ubiquitination | 22 | 3.06E-05 | 2.73 |
| GO:0051351~positive regulation of ligase activity | 20 | 3.96E-05 | 2.86 |
| GO:0051437~positive regulation of ubiquitin-protein ligase activity during mitotic cell cycle | 19 | 4.93E-05 | 2.92 |
| GO:0031396~regulation of protein ubiquitination | 24 | 5.45E-05 | 2.51 |
| GO:0051436~negative regulation of ubiquitin-protein ligase activity during mitotic cell cycle | 18 | 9.34E-05 | 2.89 |
| GO:0031400~negative regulation of protein modification process | 26 | 1.28E-04 | 2.28 |
| GO:0051352~negative regulation of ligase activity | 18 | 1.40E-04 | 2.80 |
| GO:0051444~negative regulation of ubiquitin-protein ligase activity | 18 | 1.40E-04 | 2.80 |
| GO:0043086~negative regulation of catalytic activity | 46 | 2.84E-04 | 1.73 |
| GO:0044092~negative regulation of molecular function | 53 | 2.92E-04 | 1.66 |
| GO:0031145~anaphase-promoting complex-dependent proteasomal ubiquitin-dependent protein catabolic process | 17 | 3.18E-04 | 2.73 |
| GO:0031399~regulation of protein modification process | 48 | 3.32E-04 | 1.70 |
| GO:0031397~negative regulation of protein ubiquitination | 18 | 5.02E-04 | 2.54 |
| GO:0043161~proteasomal ubiquitin-dependent protein catabolic process | 22 | 5.71E-04 | 2.25 |
| GO:0010498~proteasomal protein catabolic process | 22 | 5.71E-04 | 2.25 |
| GO:0031401~positive regulation of protein modification process | 31 | 0.003 | 1.73 |
| GO:0006511~ubiquitin-dependent protein catabolic process | 37 | 0.005 | 1.60 |
| GO:0032270~positive regulation of cellular protein metabolic process | 34 | 0.014 | 1.52 |
| GO:0051247~positive regulation of protein metabolic process | 35 | 0.016 | 1.50 |
| Cluster 4 (Enrichment Score: 3.53) | | | |
| GO:0051325~interphase | 25 | 4.96E-05 | 2.46 |
| GO:0051329~interphase of mitotic cell cycle | 24 | 8.87E-05 | 2.43 |
| GO:0000082~G1/S transition of mitotic cell cycle | 13 | 0.006 | 2.42 |
| Cluster 5 (Enrichment Score: 3.53) | | | |
| GO:0044265~cellular macromolecule catabolic process | 86 | 1.37E-04 | 1.50 |
| GO:0044257~cellular protein catabolic process | 100 | 1.59E-04 | 1.44 |
| GO:0009057~macromolecule catabolic process | 86 | 1.61E-04 | 1.49 |
| GO:0030163~protein catabolic process | 104 | 4.32E-04 | 1.39 |
| GO:0019941~modification-dependent protein catabolic process | 86 | 4.51E-04 | 1.44 |
| GO:0043632~modification-dependent macromolecule catabolic process | 79 | 8.97E-04 | 1.44 |
| GO:0006508~proteolysis | 79 | 8.97E-04 | 1.44 |
| GO:0006511~ubiquitin-dependent protein catabolic process | 127 | 0.005 | 1.26 |
| hsa04621:NOD-like receptor signaling pathway | 16 | 0.001 | 2.45 |
| hsa00601:Glycosphingolipid biosynthesis | 9 | 0.003 | 3.42 |
| hsa05120:Epithelial cell signaling in Helicobacter pylori infection | 16 | 0.004 | 2.24 |
| hsa04144:Endocytosis | 32 | 0.005 | 1.65 |
| hsa04210:Apoptosis | 18 | 0.008 | 1.974 |
| hsa04110:Cell cycle | 23 | 0.010 | 1.75 |
